# Assessing the impact of COVID-19 border restrictions on dengue transmission in Yunnan Province, China: an observational epidemiological and phylogenetic analysis

**DOI:** 10.1016/j.lanwpc.2021.100259

**Published:** 2021-08-20

**Authors:** Naizhe Li, Yun Feng, Bram Vrancken, Yuyang Chen, Lu Dong, Qiqi Yang, Moritz U.G. Kraemer, Oliver G. Pybus, Hailin Zhang, Oliver J. Brady, Huaiyu Tian

**Affiliations:** 1 State Key Laboratory of Remote Sensing Science, Center for Global Change and Public Health, College of Global Change and Earth System Science, Beijing Normal University, Beijing, China; 2 College of Life Sciences, Beijing Normal University, Beijing, China; 3 Yunnan Institute of Endemic Diseases Control and Prevention, Yunnan Provincial Key Laboratory for Zoonosis Control and Prevention, Dali, China; 4 Department of Microbiology and Immunology, Rega Institute, Laboratory of Evolutionary and Computational Virology, KU Leuven, Leuven, Belgium; 5 Department of Ecology and Evolutionary Biology, Princeton University, Princeton, NJ, USA; 6 Department of Zoology, University of Oxford, Oxford, UK; 7 Harvard Medical School, Harvard University, Boston, MA, USA; 8 Boston Children's Hospital, Boston, MA, USA; 9 Department of Pathobiology and Population Science, The Royal Veterinary College, London, UK; 10 Centre for the Mathematical Modelling of Infectious Diseases, London School of Hygiene & Tropical Medicine, London, UK; 11 Department of Infectious Disease Epidemiology, Faculty of Epidemiology and Population Health, London School of Hygiene & Tropical Medicine, London, UK

**Keywords:** COVID-19 border restrictions, dengue expansion, mosquito-borne viruses, lockdown

## Abstract

Background: In response to the COVID-19 pandemic, China implemented strict restrictions on cross-border travel to prevent disease importation. Yunnan, a Chinese province that borders dengue-endemic countries in Southeast Asia, experienced unprecedented reduction in dengue, from 6840 recorded cases in 2019 to 260 in 2020.

Methods: Using a combination of epidemiological and virus genomic data, collected from 2013 to 2020 in Yunnan and neighbouring countries, we conduct a series of analyses to characterise the role of virus importation in driving dengue dynamics in Yunnan and assess the association between recent international travel restrictions and the decline in dengue reported in Yunnan in 2020.

Findings: We find strong evidence that dengue incidence between 2013-2019 in Yunnan was closely linked with international importation of cases. A 0-2 month lag in incidence not explained by seasonal differences, absence of local transmission in the winter, effective reproductive numbers < 1 (as estimated independently using genetic data) and diverse cosmopolitan dengue virus phylogenies all suggest dengue is non-endemic in Yunnan. Using a multivariate statistical model we show that the substantial decline in dengue incidence observed in Yunnan in 2020 but not in neighbouring countries is closely associated with the timing of international travel restrictions, even after accounting for other environmental drivers of dengue incidence.

Interpretation: We conclude that Yunnan is a regional sink for DENV lineage movement and that border restrictions may have substantially reduced dengue burden in 2020, potentially averting thousands of cases. Targeted testing and surveillance of travelers returning from high-risk areas could help to inform public health strategies to minimise or even eliminate dengue outbreaks in non-endemic settings like southern China.

Funding: Funding for this study was provided by National Key Research and Development Program of China, Beijing Science and Technology Planning Project (Z201100005420010); Beijing Natural Science Foundation (JQ18025); Beijing Advanced Innovation Program for Land Surface Science; National Natural Science Foundation of China (82073616); Young Elite Scientist Sponsorship Program by CAST (YESS) (2018QNRC001); H.T., O.P.G. and M.U.G.K. acknowledge support from the Oxford Martin School. O.J.B was supported by a Wellcome Trust Sir Henry Wellcome Fellowship (206471/Z/17/Z).

Chinese translation of the abstract (Appendix 2)

**Research in context**

**Evidence before this study**

We searched PubMed from up to Feb 16, 2021, without date limits or language restrictions, using the search terms “(COVID-19 OR coronavirus OR sars-cov-2) AND (lockdown OR interventions OR restriction) AND (dengue* OR DENV*)”. In addition, we searched Health Information Platform for the Americas for data cases reported in Pan American countries. We found that the impact of COVID-19 lockdowns on dengue transmission was unclear. Pakistan, Peru, Singapore, Thailand, Ecuador, Paraguay and Bolivia reported larger outbreaks in 2020. However, Bhutan, Sri Lanka and parts of Brazil and Colombia have reported lower than average dengue case counts. The studies mainly focused on dengue endemic countries and not on areas where dengue transmission is likely initiated by importation.

**Added value of this study**

We investigated the transmission history of DENV in Yunnan, an area that neighbours dengue-endemic countries, using detailed genomic and epidemiological data. Our results identified Yunnan as a net importer of dengue lineages and found evidence that COVID-19 border restrictions might have substantially reduced dengue incidence in non-endemic areas. This provides the first evidence to suggest that COVID-19 related restrictions can reduce dengue transmission in non-endemic areas.

**Implications of all the available evidence**

The impacts of travel restrictions, due to the COVID-19 pandemic, on dengue need to be characterised to inform short- and long-term plans for dengue control. Our findings show that border restrictions can mitigate dengue transmission in non-endemic areas and identify the importance of international human mobility for dengue spread.

Alt-text: Unlabelled box

## Introduction

Dengue is responsible for 10,000 deaths annually^1^ and is responsible for the largest burden of all the arboviral diseases, with an estimated 100 million symptomatic infections per year globally.^2^ The number of dengue cases reported to WHO has increased over 8 fold over the last two decades, from 0.5 million in 2000, to > 2.4 million in 2010, and to 4.2 million in 2019^3^ and it has been estimated that over 6.1 billion people might be at risk of dengue by 2080.^4^ Dengue virus (DENV) is a mosquito-borne virus and a member of the Flaviviridae family.^5^^,^^6^ Vector control and community engagement remain the main strategies to reduce dengue burden. Although the first vaccine against dengue has been licensed, its use is limited because pre-screening for serostatus is required and only those identified as seropositive individuals can receive the vaccine.^7^ Furthermore, increasing international movement, and thus introduction of DENV via viremic humans, has facilitated geographic expansion. Even small numbers of imported dengue cases, i.e. DENV introductions, can trigger locally acquired infections and even local outbreaks.^8^

In response to the COVID-19 pandemic, a series of nonpharmaceutical interventions (NPIs) have been used to control the disease spread globally.^9^ To avoid imported cases, China implemented strict lockdowns and immigration controls on the border ports since early 2020. Lockdowns and travel restrictions have led to substantial reductions in the burden of infections that are transmitted from human to human, such as influenza^10^ and measles,^11^ however the impact of COVID-19 lockdowns on dengue transmission remains unknown.^12^ Worse than average dengue incidence has so far been reported from various countries.[13], [14], [15] In contrast, China reported a substantial reduction of dengue cases in 2020 (a total of 810 cases), compared to previous years (22201 in 2019, 5117 in 2018 and 5897 in 2017).^16^ Yunnan Province, located at the fringe of the global distribution of dengue, across Tropic of Cancer and inside the 10°C winter isotherm,^17^ borders Myanmar and Laos, dengue-endemic counties in Southeast Asia, and has recently experienced its largest-recorded dengue outbreak in decades in 2019. While lockdowns in Yunnan ended in early March, international travel restrictions remained throughout 2020, presenting a unique opportunity to quantify the role of trans-border human movement in shaping dengue dynamics in southern China.

Despite international movement restrictions in 2020, some countries have reported worse than average dengue incidence. It has been hypothesised that such increases could be due to lockdowns disrupting routine vector control programs.^12^ More time spent at home may also increase the transmission of vector-borne diseases in settings where household-transmission predominates.^18^ At a city or national scale, human movement plays an important role in the spread of dengue.^19^ NPIs can cut down the transmission links between different areas. These protective impacts of movement restrictions will be most pronounced in non-endemic areas, such as Yunnan and studying the impact of border restrictions in such areas can give new insights into the role of international movement in the spread of dengue. In particular they can help distinguish areas that are sources (net exporters) or sinks (net importers) of DENV transmission. Targeting surveillance and quarantine measures in DENV sinks and, in particular towards travelers arriving from DENV sources could result in a large reduction in transmission.

In this study, we combine detailed genomic and epidemiological data from both imported and locally acquired dengue cases during 2013–2020 to assess the effect of border-restrictions on dengue spread. We reconstruct the dispersal history and transmission pattern of DENV in this area using phylogeographic inference. Specifically, we aim to investigate how dependent dengue dynamics in Yunnan are on international importation of DENV via infected travelers from dengue-endemic tropical countries. Our findings aim to provide new insights for dengue prevention and control.

## Methods

### Source of case data

Notified dengue cases in Yunnan Province between January 2013 and December 2020 were obtained from the Yunnan Center for Disease Control and Prevention (CDC). In China, dengue is a class B notifiable infectious disease and all human cases must be reported to the Chinese CDC by law. Dengue cases were confirmed according to clinical diagnosis and laboratory confirmation according to diagnostic criteria (WS 216-2008, WS216-2018) from the Ministry of Health of the People's Republic of China. An imported case of dengue fever is defined as a patient who had traveled to another country, in which dengue fever is endemic, with a bite history within 15 days prior to the onset of illness. All cases were distinguished as imported or locally acquired dengue cases. Monthly reported cases in Ruili and Jinghong, two border cities of Yunnan, were also collected from Yunnan CDC and confirmed according to the same criteria. The reported cases in Viet Nam, Laos, Thailand, and Myanmar were obtained from the WHO Regional Office for the Western Pacific's Institutional Repository for Information Sharing (WPRO IRIS, http://apps.who.int/iris/), Ministry of Public Health in Thailand (MoPH, http://www.boe.moph.go.th/boedb/surdata/index.php) and European Centre for Disease Prevention and Control (ECDC, https://www.ecdc.europa.eu/en/dengue-monthly). If a week spanned across months, we apportioned cases based on the proportion of days of the overlapping week in each month. The demographic data were collected from United Nations Demographic and Social Statistics (https://unstats.un.org/unsd/demographic-social/) and Yunnan Bureau of Statistics (http://stats.yn.gov.cn/).

### Source of sequence data

A total of 243 DENV E gene sequences were obtained using serotype identification primers from patients with dengue fever in Yunnan during 2013-2017, as previously published elsewhere.[20], [21], [22] DENV (DENV-1 to DENV-4) envelope (E) gene sequences with known collection dates and locations of sampling in Asia were also collected from GenBank. The sequences dataset comprised published and available sequences, as of November 2020. The remaining strains comprised a total of 15,368 sequences sampled between 1956 and 2020, from 20 distinct countries or geographic regions. The recombinant and high similarity (>99%) strains were removed and in order to control for possible bias from uneven sampling, we randomly subsampled the complete sequence datasets by location and sampling time. At most 10 sequences were sampled per country and per year in order to create a more equitable spatio-temporal sampling distribution. We reconstructed ML trees using Fasttree^23^ and the clusters containing Yunnan strains were kept. The final datasets for each serotype were prepared separately and the final dataset contained 970 DENV-1 sequences, 725 DENV-2 sequences, 704 DENV-3 sequences, and 591 DENV-4 sequences. We defined 28 regions to which the taxa were allocated, including 15 countries and 13 provinces in China. Sequences were grouped by serotype and aligned separately using MAFFT.^24^ Recombination was inspected using the methods implemented in RDP4^25^ and SimPlot.^26^

**Climatic data and Vector Suitability Scores** Climatic suitability scores were estimated by collating data on *Aedes* mosquitoes, temperature, relative humidity and precipitation. We used mean values of Ruili and Jinghong to represent Yunnan. The data from capital cities were collected to represent each country. We used monthly *Aedes* aegypti suitability maps at a 5km×5km spatial resolution, and then aggregated these high-resolution maps at city level in Yunnan.^27^^,^^28^ Meteorological data were obtained from the World Weather Online (https://www.worldweatheronline.com/).

### Statistical analysis

A generalised liner model (GLM) with quasi-Poisson distribution was established to quantify the relationship between border restrictions (exposure) and dengue cases in Yunnan (outcome). Climate factors (average temperature, relative humidity, and total precipitation) with 0-3 month lags were included as potential alternative variables and a binary variable to represent border restrictions (the exposure) was included in the model. Two fixed effect variables, *year* and *month*, were also included to control long-term trend and seasonality (additional unobserved confounding). Annual ppopulation (on the log scale) was included as an offset in the model to account for changing population sizes. The variables were selected using a forward stepwise regression method and the best model was chose based on the Quasi Akaike Information Criterion (QAIC). The selected regression model was as follows:

$$\begin{array}{cc} & Y_{t}∼\mathrm{Quas}\mathrm{ipoi}\mathrm{sson}\left(\mathrm{dengue}\;{\mathrm{case}}_{t}\right)\\ & \log\left(\mathrm{dengue}\;{\mathrm{case}}_{t}\right)=\alpha +\beta_{1}\mathrm{Te}m_{t-1}+\beta_{2}\mathrm{Hu}m_{t-1}+\beta_{3}BR_{t}\\ & +\mathrm{Month}+\mathrm{Year}+\text{offset}\left(l\text{og}\left(\mathrm{pop}\right)\right) \end{array}$$

*dengue case_t_* represents number of reported cases in month *t*. The *α* is the intercept. The *β_j_*’s are the regression coefficients. *Tem_t-1_* represents the average temperature in Yunnan in month *t-1. Hum_t-1_* is the relative humidity in Yunnan in month *t-1. BR_t_* is used to identify whether the border restriction is conducted in month *t. BR* was set to 1 between March and the end of the time series.^29^ We do not consider the effects of other NPIs such as stay at home orders or social distancing because human mobility data (Baidu migration flows database, http://qianxi.baidu.com/ ) suggest that human movement had already rebounded to pre-pandemic levels by mid-March, long before the usual June-November dengue season in Yunnan (Figure S10). The regression was performed using the R version 4.1.0.

In addition to the regression analysis we also assessed the regional correlation in dengue incidence between Yunnan and its neighbouring countries (each country tested independently) using Pearson's correlation coefficient on incidence values at 0-3 month lags. This was assessed separately for data 2013-2019 and for 2020 to assess the impact of border restrictions on regional synchrony in dengue incidence. This lagged correlation analysis was also repeated for climate variables.

### Birth-death skyline analyses

To analyse the transmission dynamics of DENV in Yunnan, the birth-death skyline model implemented within BEAST2 was used.^30^ We used sequences from Yunnan and the total number of sequences analysed here was 138 for DENV-1, 107 for DENV-2, 125 for DENV-3, and 30 for DENV-4. A log-normal prior with a mean value (M) of 0.0 and a variance (S) of 1 was specified for the effective reproductive number (R). For the recovery rate, a log-normal prior with M = 3 and S = 0.5 (95% confidence interval 8.82-45.7, corresponding to an infectious period of between 8.0 and 41.3 days) was used. The sampling probability was estimated using a uniform (beta (1.0, 1.0)) prior. The origin of the epidemic was estimated by means of a log-normal prior with M = 3 and S = 0.5. The MCMC analyses were run for 50 million generations, sampling every 5,000 steps. Convergence was assessed based on ESS values (ESS >200). Uncertainty in the estimates was indicated by 95% highest posterior density (95% HPD) intervals.

### Molecular clock and phylogeographic analyses

For each serotype subsampled data set, we first evaluated the strength of the temporal signal using TempEst.^31^ This revealed a strong and positive correlation between genetic divergence and sampling time for each data set (*r* = 0.75 for DENV-1; *r* = 0.84 for DENV-2; *r* = 0.87 for DENV-3; *r* = 0.76 for DENV-4). Time-stamped phylogenies were estimated using the Bayesian MCMC approach implemented in BEAST v1.10.4.^32^ For each serotype, three independent Markov chain Monte Carlo (MCMC) chains were computed for 500 million steps, sampling every 50,000 steps after the removal of 10% burn-in. An uncorrelated lognormal relaxed molecular clock model^33^ and a coalescent-based tree topology prior with Gaussian Markov random field (GMRF) based time-aware smoothing were used.^34^ The tree prior describes how the population size is expected to change over time. Using a skyride model, we assumed a smooth process on population size changes. The substitution process describes changes in the rate of molecular evolution and was modelled as a GTR+I+Γ process. A non-informative continuous-time Markov chain (CTMC) reference prior^35^ was specified on the mean clock rate. The BEAGLE library was used to accelerate computation.^36^ Analyses were combined after discarding the first 10% of the chain as burn-in, and convergence and mixing properties were evaluated with Tracer v1.7.^37^ A total of 10,000 trees was generated from the MCMC chain and used as an empirical tree distribution for the subsequent phylogeographic reconstructions.

To reconstruct the spatial dynamics of DENV-1–4, we conducted phylogeographic analyses to infer ancestral branch locations using the Bayesian asymmetric discrete trait evolution model, ^38^ as implemented in BEAST v1.10.4.^32^ For each serotype, support for non-zero rates of exchange between all pairs of locations was evaluated using Bayesian stochastic search variable selection (BSSVS). Maximum clade credibility (MCC) trees were summarised using TreeAnnotator and visualised using SPREAD3 from the posterior distribution of trees.^39^ Estimates of the posterior expected number of migration events between all pairs of locations (Markov jumps) were computed through stochastic mapping techniques.^34^^,^^40^

### Role of the funding source

The funders had no role in study design, data collection and analysis, the decision to publish, or in preparation of the manuscript.

## Results

### Declining dengue incidence in Yunnan in 2020 is associated with border restrictions

**Fig. 1:**
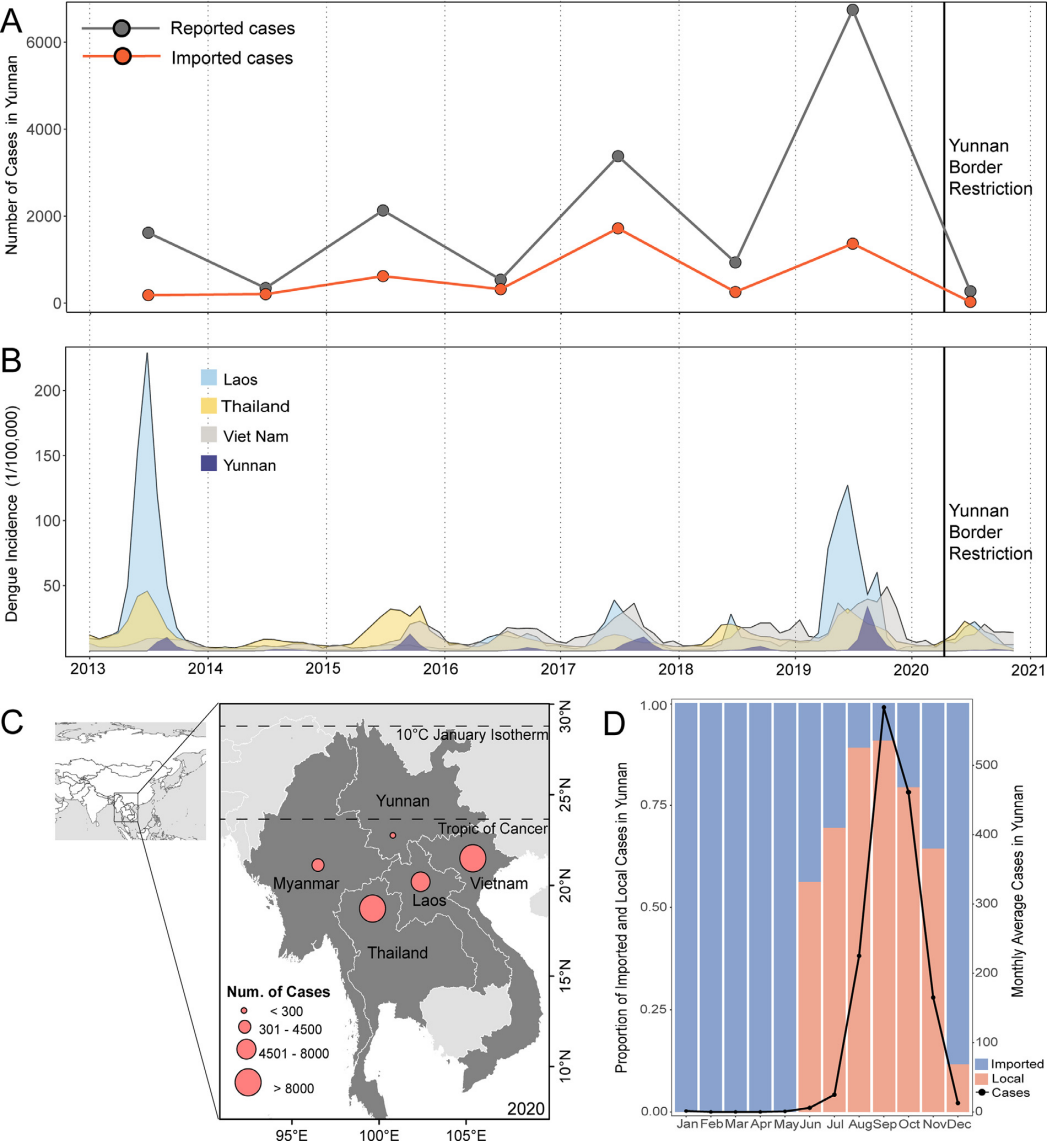
Border restriction and dengue transmission dynamics in areas in China-Myanmar-Laos border, 2013-2020. (A) Annual number of total reported cases and imported cases in Yunnan, China. The vertical line is the start of Yunnan border restriction. Black line, total reported cases; orange line, imported cases. (B) Monthly dengue incidence in Yunnan and near by countries. (C) China-Myanmar-Laos border. Point size represents the number of dengue cases in 2020. Dotted line is the Tropic of Cancer, 23.5 degrees north of the equator, and 10°C January isotherm, respectively. (D) Proportion of seasonal imported (blue), locally acquired (orange) and monthly average of dengue cases (black line) in Yunnan, 2013–2020. Noted: Epidemiological data of Myanmar used in panel C was collected as of July, 2020. Data used in panel D was collected from Ruili and Jinghong.

During 2013–2019, more than 15000 dengue cases were reported in Yunnan with a peak in 2019 (Fig. 1A). The year 2020 has seen a substantial reduction relative to this historical trend with a decline from 6840 reported dengue cases in 2019 to 260 in 2020, whereas there was no obvious reduction relative to the historical average in the bordering countries (Fig. 1B). Compared with the average annual incidence during 2013-2019, dengue incidence in 2020 reduced by 88.4% in Yunnan, 54.8% in Laos, 27.2% in Thailand and 11.6% in Viet Nam. The number of reported cases in bordered countries were notably higher than Yunnan in 2020 (Fig. 1C). Compared to 2016, the last year with a dengue outbreak in neighbouring countries of comparable size to 2020(Table S3), the number of cases in Yunnan had reduced by 50.5% (260 *vs.* 525).

To assess whether the 2020 reduction in Yunnan could be explained by dengue inter or intra-annual trends or abnormal weather conditions, we fit a generalised linear model that includes temperature, rainfall and relative humidity and temporal effects in addition to border restrictions. The “border restriction” variable was retained in model selection procedures and a significant negative association with dengue incidence in Yunnan was found (coefficient = -2.96, *P* < 0.01, Table S6). The model predicted 4774 reported cases if border restrictions were not conducted compared to 249 in the presence of border restrictions (risk ratio of 19.2, observed cases: 260).

To further investigate the usual dengue season synchrony between Yunnan and neighbouring countries we examine data in two border cities of Yunnan, Ruili and Jinghong where monthly dengue case data were available. Considering Ruili and Jinghong were the most affected cities and reported 86% of locally acquired cases and 73% of total dengue cases during 2013-2020 in Yunnan, we assumed the data were representative of wider dengue dynamics in Yunnan. We found that between 2013 and 2019 monthly dengue incidence in Yunnan is correlated with dengue incidence in border countries with a 0-2 month lag (Pearson *r* = 0.59, *P* < 0.01, 2-month lag with Laos; Pearson *r* = 0.57, *P* < 0.01, 2-month lag with Thailand; Pearson *r* = 0.54, *P* < 0.01, 0-month lag with Viet Nam). To see if this lag was solely due to lagged environmental drivers of DENV transmission, we analysed the correlation of the climate factors between Yunnan and border countries. We found 1-month lagged peak correlation in humidity but no lagged correlation in temperature and precipitation (Fig S3, Table S7). This means that the 0-2 month lag between elevated dengue incidence in Yunnan and its neighbouring countries is unlikely to be explained by lags in climate alone and more likely involves delayed introduction then spread of the epidemic within Yunnan. In the year 2020 this regional synchrony was not present with no evidence of significant correlation with dengue dynamics in Thailand or Laos and only moderate correlation with Viet Nam (Pearson *r* = 0.58, *P* < 0.05). We were unable to assess the correlation between dengue cases in Yunnan with those in Myanmar in 2020 as data was not available form this country at the time of analysis.

Combined, these analyses show that Yunnan has seen a much bigger decline and disruption of dengue dynamics in 2020 than its regional neighbours and that this decline is significantly associated with the timing of border restrictions.

## Characterizing the role of dengue importation in shaping dengue dynamics in Yunnan 2013-2019

To further understand why border restrictions had such a large impact on dengue dynamics in Yunnan we also analyse epidemiological and genomic data from the years 2013-2019 to show how case importation and seasonality play a critical role in dengue dynamics in Yunnan.

Historically, there has been a close correlation between the annual number of imported cases and the total number of cases in Yunnan (Fig. 1A, Pearson *r* = 0.88, *P* < 0.01). Overall, the number of locally acquired cases was far higher than imported cases, with a proportion of 69.2%. Imported cases were reported all year round but outbreaks of local transmission were restricted to summer periods (June-December, Fig. 1D). Combined these epidemiological data sources suggest that transmission does not persist through the winter, but is re-introduced seasonally and that the size of that re-introduction, along with environmental factors that affect *Aedes* mosquito population dynamics, likely determine the size of the epidemic.

**Fig. 2:**
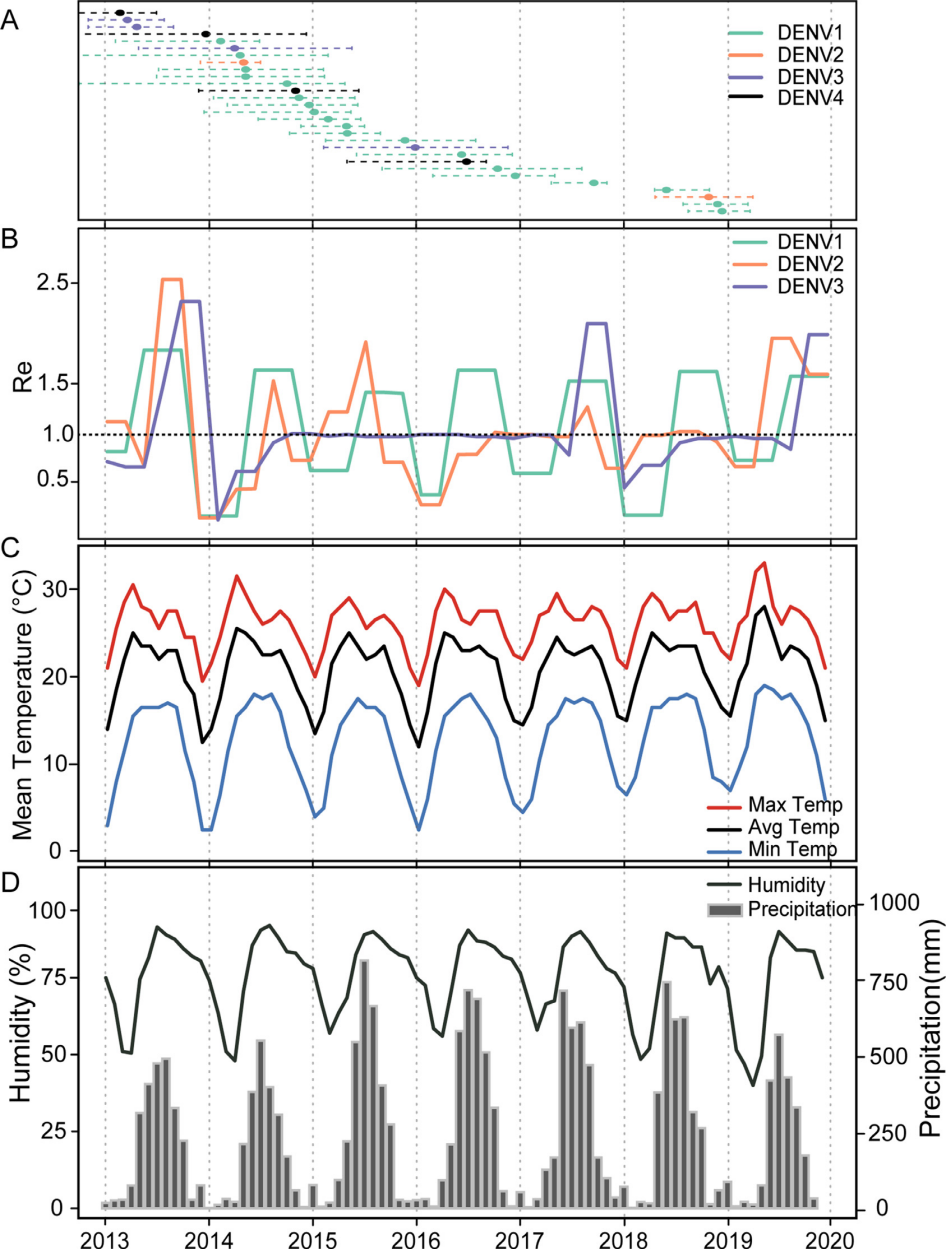
Genomic epidemiology of DENV and climatic variables in Yunnan. (A) The inferred time of the most recent common ancestor (TMRCA) of DENV-1 (green), DENV-2 (orange), DENV-3 (purple) and DENV-4 (black) introduction to Yunnan. The points represent the mean date and the dotted lines show the 95% highest posterior density. (B) Time-varying effective reproductive numbers (Re), estimated using a birth–death skyline approach. Solid line represents the median posterior estimate of Re for each DENV type. Insufficient DENV-4 sequences were available to include in this analysis. (C) Mean temperature of Ruili and Jinghong. (D) Mean relative humidity and precipitation of Ruili and Jinghong.

We used Bayesian birth-death skyline analysis (BDSKY)^41^ to estimate the effective reproductive number (R_e_) from the DENV sequences in Yunnan. R_e_ is defined as the average number of secondary infections caused by a single infectious person over the course of their infection (R_e_ > 1 and R_e_ < 1 represents epidemic growth and decline, respectively). Although DENV introduction events occurred year-round (Fig. 2A), we observed a clear seasonal pattern of epidemic growth (Fig. 2B), R_e_ > 1 between June and November, which coincided with the timing of local transmission in the epidemiological data (Fig. 1D). While at least one serotype had estimates of R_e_ > 1 within this summer transmission window each year, not every serotype was capable of epidemic growth every year (e.g. only DENV 1 in 2016 and 2018). This may suggest that despite relatively low transmission levels relative to bordering countries, DENV selection due to population immunity may still be occurring in Yunnan. Estimated R_e_ values for Yunnan also showed close correlation with temperature, humidity, precipitation (Fig. 2C, 2D) and mosquito suitability (Fig S4, Table S8). These correlations were robust to uncertainty in effective reproduction number estimation findings (Fig. S5, Table S9). This provides further evidence that dengue transmission is unsustainable over the winter months in Yunnan and that these unsuitable periods are due to unfavorable weather conditions which is consistent with our theoretical and observational understanding of how climate affects global dengue dynamics^27^^,^[42], [43], [44]

**Fig. 3:**
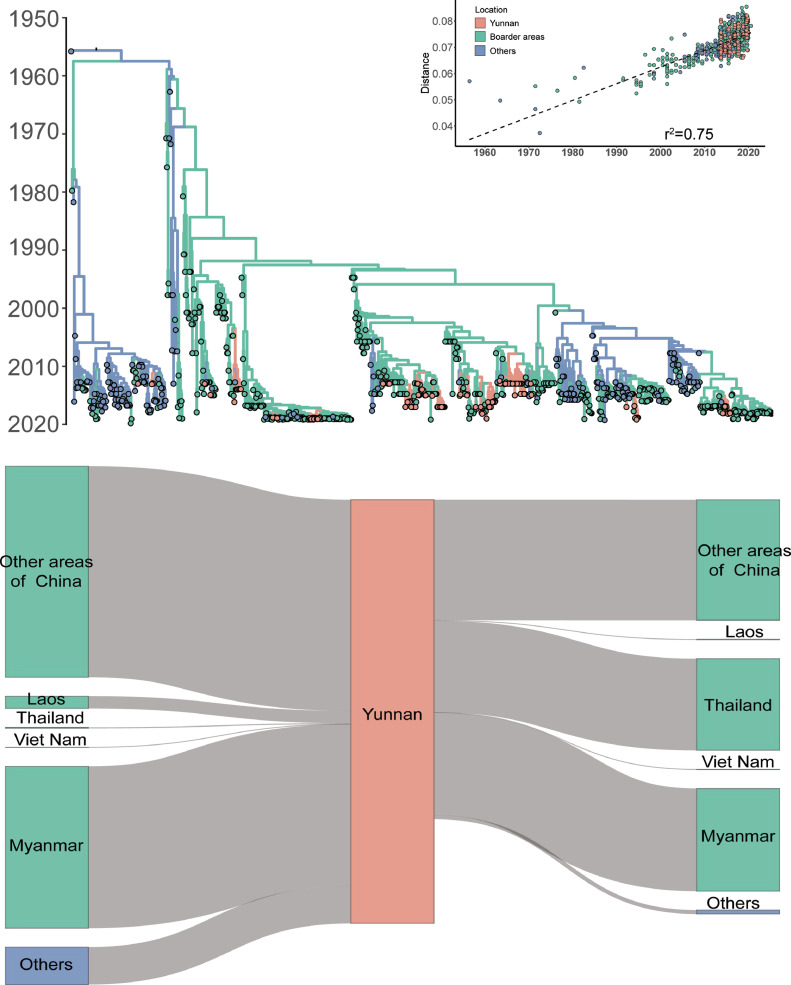
Sources of dengue virus introduction at China-Myanmar-Laos border. The upper panel shows the maximum clade credibility trees of the E gene of DENV-1 (n=970). Branches and tips are colored according to Yunnan (orange), the border areas of Yunnan (green) and other areas (purple). The inset shows a root-to-tip regression of genetic divergence against dates of sample collection. The lower panel shows the estimated flows (number of expected transitions summed by DENV serotype) into (left to central) and from (central to right) Yunnan.

We used a Bayesian phylogeographic approach to reconstruct the origins and spread of DENV in Yunnan and bordering countries prior to 2020 to understand what role Yunnan normally plays in regional dengue dynamics. We find that the viruses in Yunnan were widely distributed across the phylogenetic tree and most of them clustered with viruses isolated from nearby areas (Fig. 3, Fig. S6-8). Rare continuous evolution of DENV in Yunnan over time indicated that Yunnan remained a non-endemic area and that local infections are more likely to result from imported cases.

Our results further indicate a high frequency of virus lineage migration among Yunnan and border countries (Fig. 3). Laos and Myanmar were major foreign sources of DENV in Yunnan. For DENV-1 and DENV-2, the virus is estimated to be mainly imported from Myanmar and other areas of China. Despite having lower incidence than bordering countries and lagged epidemics, Yunnan was still a significant exporter of DENV lineages. Significant links from Yunnan to Myanmar, Thailand and other areas of China were found. Notably, DENV-2 showed more exportation than importation events, which was unique among the 4 serotypes Fig. S9). No evidence of DENV-3 migration was found between Myanmar and Yunnan, but neighbouring Laos was identified as an important foreign source of DENV-3 for Yunnan. For DENV-4, on the other hand, Myanmar was the almost exclusive source. However, due to data availability and undersampling of DENV genomes, we cannot rule out other source areas. These results suggest that international spread plays an important role in regional dengue dynamics and that even non-endemic areas repeatedly export DENVs that initiate transmission in other endemic and non-endemic areas.

## Discussion

Using a combination of epidemiological and phylogenetic data we show that DENV transmission in Yunnan is only made possible by frequent re-introduction. By showing that (i) there are no locally-acquired cases during the winter, (ii) local epidemics consistently are preceded by those in neighbouring areas despite similar climates, (iii) that the size of local epidemic correlates with the number of imported cases, (iv) R_e_ (as estimated by independent genomic data) is consistently below 1 throughout the winter and (v) intermixing of locally acquired DENV sequences among those from neighbouring countries in the phylogenies, we provide the most comprehensive collection of evidence to date that Yunnan is a sink of DENV transmission. These findings combined with the close correlation between border restrictions and the unexpectedly low levels of dengue in Yunnan in 2020 lead us to conclude that international travel restrictions reduce dengue risk in non-endemic areas such as Yunnan.

Despite Yunnan experiencing only lower-incidence, seasonal transmission driven by imported cases, it is still able to export a significant number of DENV lineages, both domestically and internationally (Table S10). Furthermore, variations in serotype-specific R_e_ may suggest that immune-driven selection of DENVs may still be occurring even in this low transmission intensity environment. This suggests that non-endemic dengue sinks may still play an important role in international DENV spread dynamics.

The current international travel restrictions present a unique opportunity to measure the contribution of international travel to dengue source and sink dynamics and understanding the global expansion of dengue. Our results illustrate that border restrictions were strongly associated with dengue mitigation in non-endemic areas and likely caused the reduction of dengue incidence in Yunnan. Due to the environmental continuity and close cultural, historical and linguistic ties, cross-border movement and trade between Yunnan and neighbouring countries is common.^45^ Previous studies have hypothesised that dengue expansion is driven by human movement[46], [47], [48] and that cross-border population movement creates great challenges for preventing the international spread of infectious diseases.[49], [50], [51], [52] While travel restrictions impose significant economic and societal impacts and are unlikely to be a proportionate tool to prevent the spread of dengue, these results do suggest a potential greater role for dengue surveillance and quarantine policies for travelers arriving from endemic countries during large outbreaks or high risk dengue seasons. Further work is needed to understand the broader implications of such policies on long-term DENV dynamics as well as other diseases whilewhi simultaneously considering their adverse societal impacts.

It has been suggested that climate plays a strong role in limiting the global distribution of dengue and that climate change may facilitate future dengue expansion.^4^ Located within 10°C January isotherm and neighbouring the Tropic of Cancer, Yunnan is at risk of transitioning to an endemic region as aresult of itioning global warming. But our analysis shows that Yunnan still remains a non-endemic dengue setting at the edge of the distribution of dengue in Asia. The R_e_ and epidemiological surveillance show that local transmission in Yunnan consistently occurs year-to-year, but only in warmer months and that dengue expansion in Yunnan is more closely associated with movement of infected humans. This suggests that future projections of the global distribution of dengue need to take into account human movement, particularly in environments that are only suitable seasonally.

Our study has several limitations. First, ideally more information on international human movement among the population and among dengue cases would be available. Even aggregate data on human transboundary movements, or the number of passengers arriving from specific destinations over time, would help disentangle the relative role of different countries in DENV spread.

Second, while we attempted to adjust for alternative variables when statistically testing the association between border restrictions and dengue incidence, immunity and underreporting could potentially introduce unmeasured confounding. Like in many other countries worldwide, the year 2019 saw exceptionally high dengue incidence in Yunnan (Fig. 1B) and immunity may have contributed to the observed decline in 2020 and hence led us to overestimate the effects of border closures. However, many countries in South East Asia also observed large dengue outbreaks in 2019 yet did not see declines in 2020 dengue incidence to the extent of that observed in Yunnan. There may also be concerns that COVID-19 related disruption may have led to underreporting of dengue cases which was not included in our analysis. While this remains to be seen, it would only affect our findings of an association if underreporting was substantially worse in Yunnan compared to neighbouring countries. Given COVID-19 lockdown restrictions in China were among the briefest in the region and only occurred outside the usual dengue season, it would be surprising if impacts on dengue surveillance in Yunnan in 2020 were greater than in neighbouring countries.

Third, because virus samples can only be extracted from a small handful of DENV infections, the extent of viral migration is an underestimation of the true frequency. This is likely more pronounced for more recent time periods due to the low case number in 2020 and data reporting lags. While underestimation of the importation frequency can be countered by prioritizing the sequencing of cases that are likely imported based on travel history, this strategy can not compensate for undersampling the virus diversity in the source regions.^53^ As transmission chains are inferred based on sequence data, undersampling also makes it difficult to conclusively distinguish between local and repeatedly introduced transmission chains. Continued monitoring of dengue infection trends in Yunnan in the period following the end of (cross-border) mobility restrictions will be important to monitor as absence of transmission in 2020 will lead to lower levels of immunity and possibly higher outbreak risk once international travel resumes.

## Declaration of Competing Interest

The authors declare no competing interests.

## Appendix Supplementary materials

Image, application 1

Image, application 2

Image, application 3
