# Supplementary material for: Assessing the impact of COVID-19 border restrictions on dengue transmission in Yunnan Province, China: an observational epidemiological and phylogenetic analysis
Source: Lancet Reg Health West Pac. 2021 Aug 20;14:100259. doi: 10.1016/j.lanwpc.2021.100259 (PMC8387751; doi:10.1016/j.lanwpc.2021.100259)
Supplement: Supplementary file 1 [file mmc1.docx]

Supplementary Information for

**Assessing the impact of COVID-19 border restrictions on dengue transmission in Yunnan Province, China: an observational epidemiological and phylogenetic analysis**

Naizhe Li†, Yun Feng†, Bram Vrancken†, Yuyang Chen, Lu Dong, Qiqi Yang, Moritz U. G. Kraemer, Oliver G. Pybus, Hailin Zhang*, Oliver J. Brady*, Huaiyu Tian*

Corresponding author:

Huaiyu Tian

Email: tianhuaiyu@gmail.com

Oliver J. Brady

Email: Oliver.Brady@lshtm.ac.uk

Hailin Zhang

Email: zhanghl715@163.com

This PDF file includes:

Figures S1 to S11

Tables S1 to S11


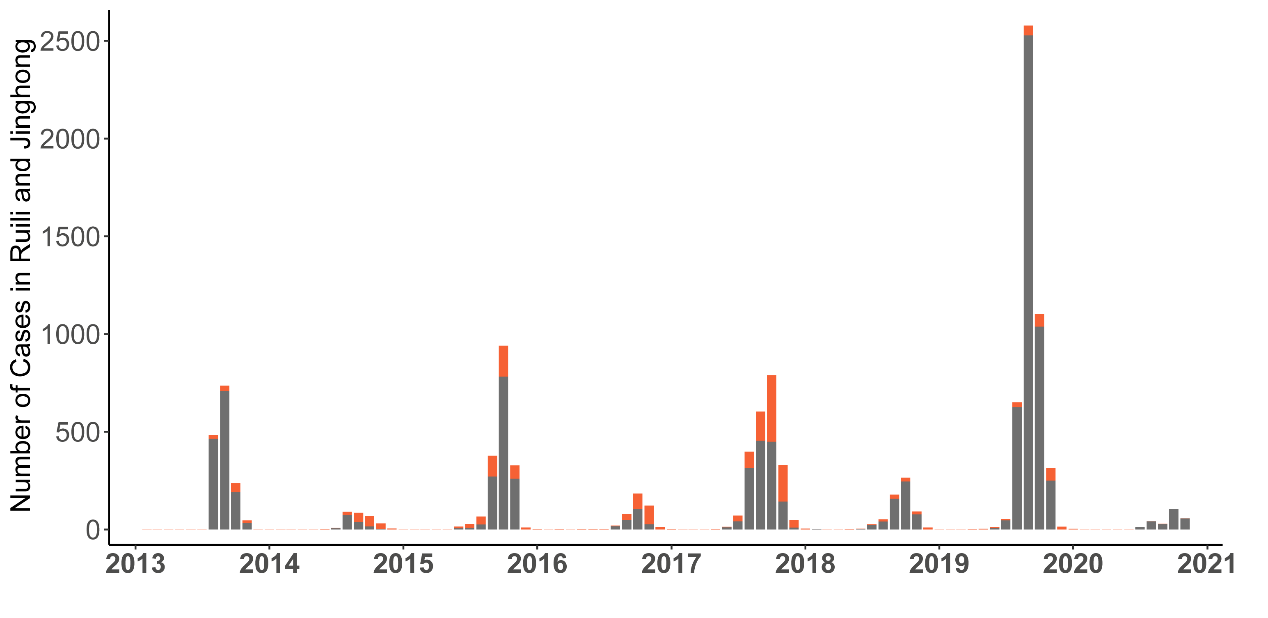


**Fig. S1 Monthly dengue cases in Ruili and Jinghong.** Locally acquired dengue cases were colored in grey and import cases were orange.


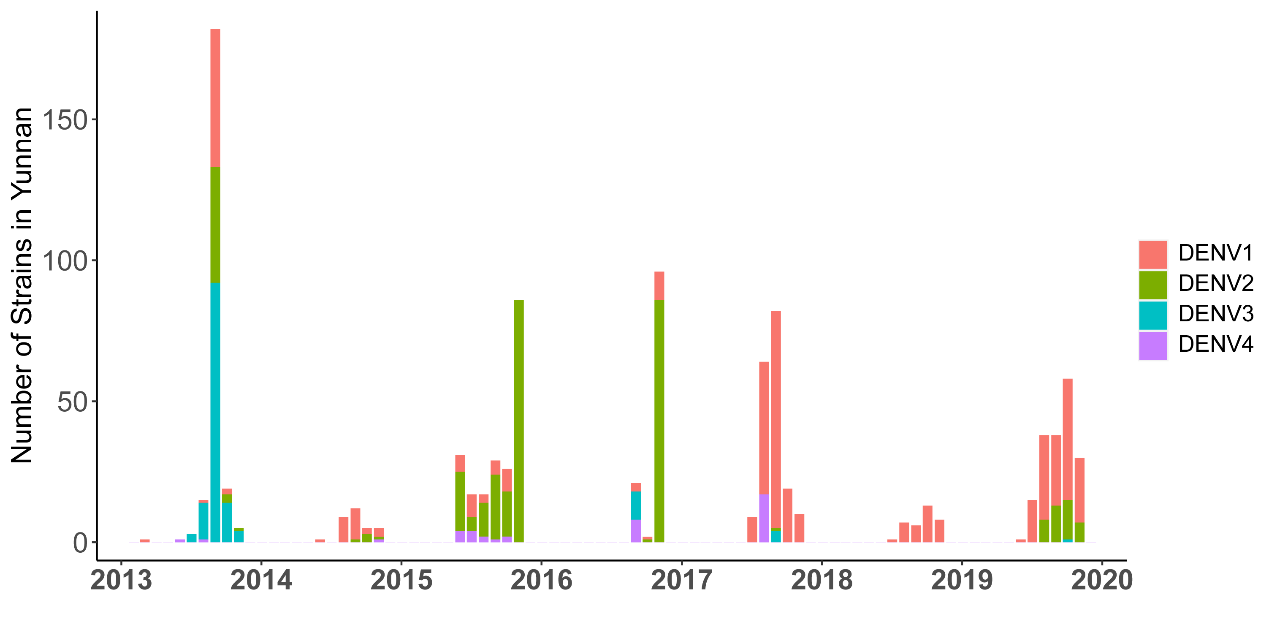


**Fig. S2 DENV sequences in Yunnan.** Samples are colored according to serotypes.


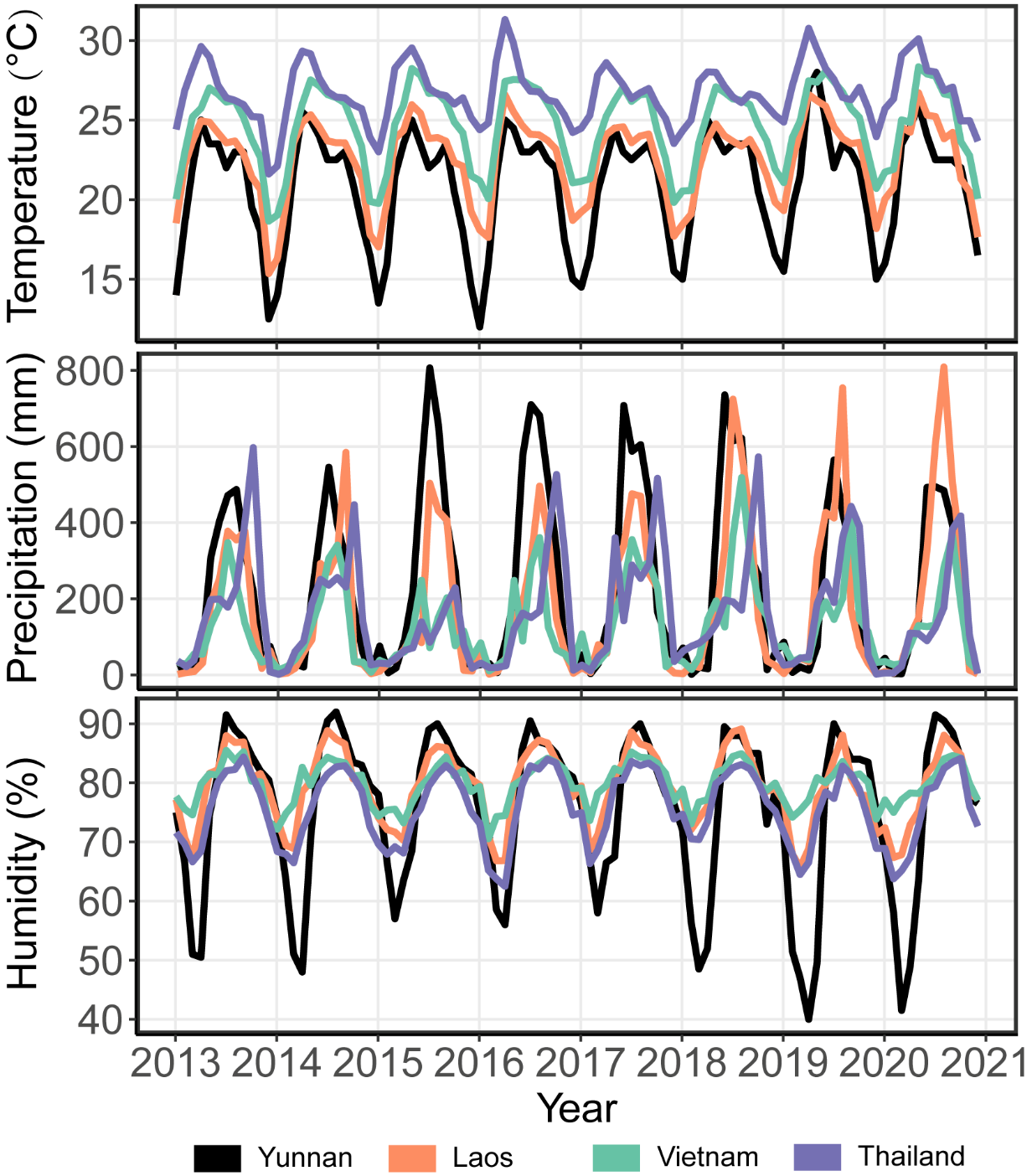


**Fig. S3 Climate factors in Yunnan and border countries during 2013-2020.**


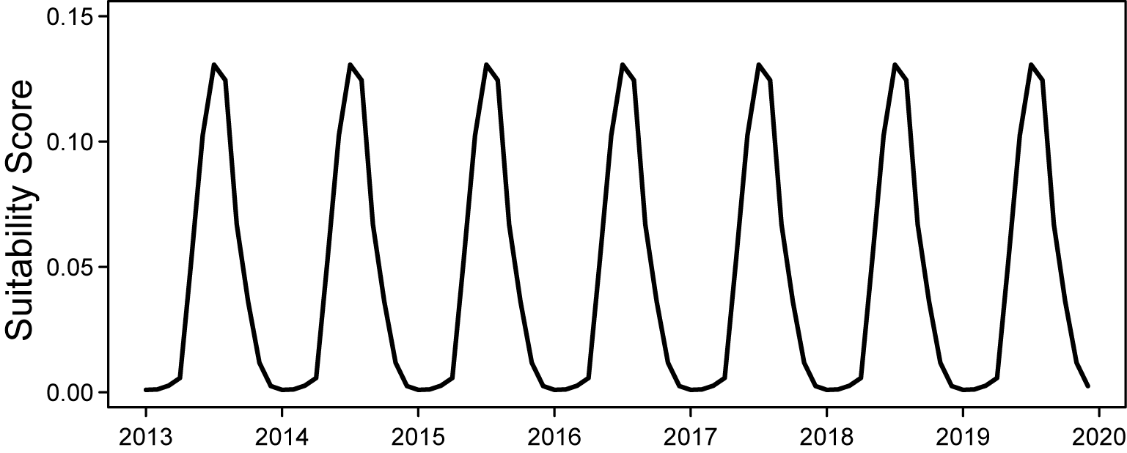


**Fig. S4 Mosquito suitability score^1,2^ in Yunnan, 2013-2020.**

*Noted, We use the data from Ruili and Jinghong*


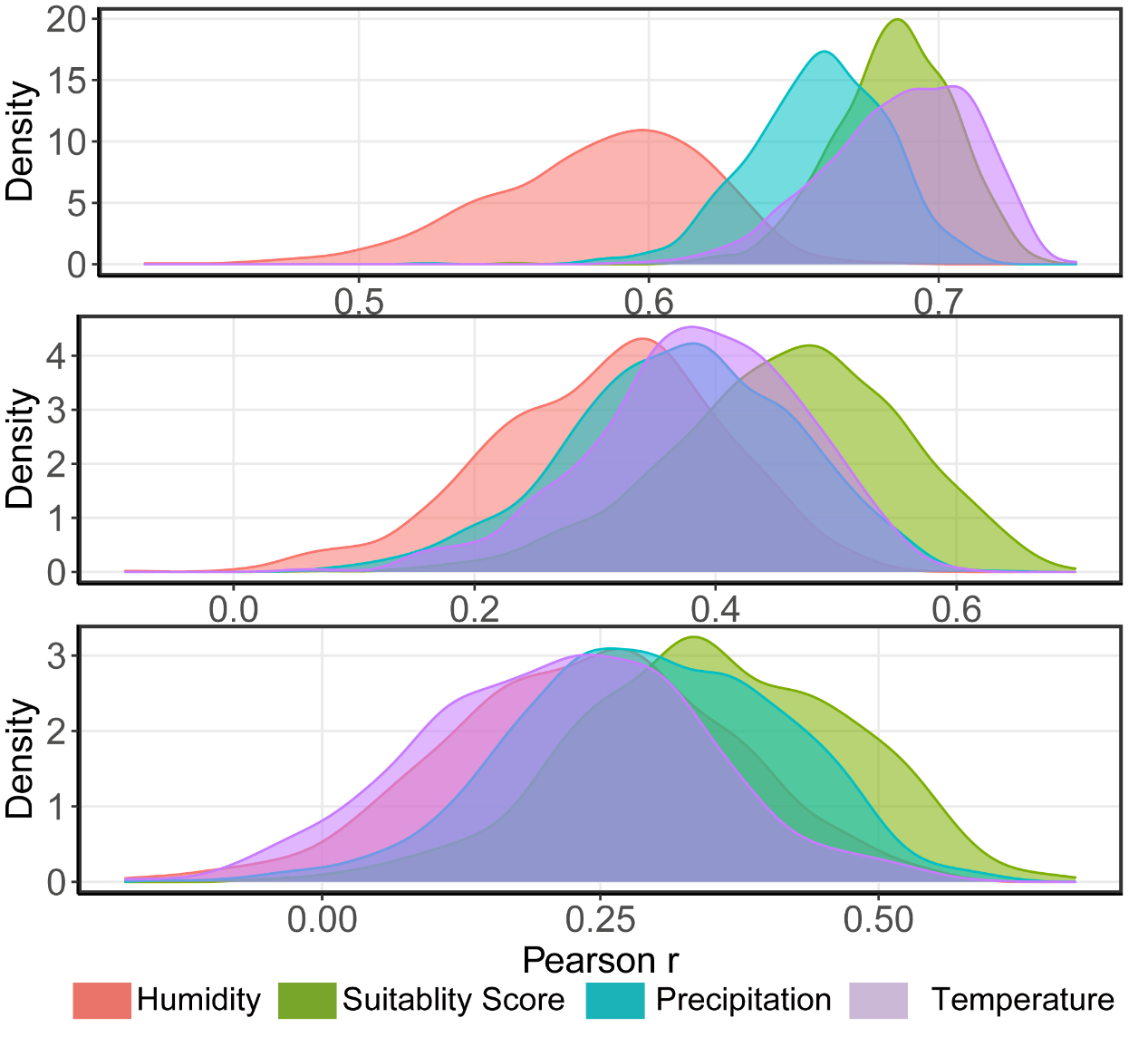


**Fig. S5 Distribution of Pearson *r* between effective reproduction number and climate factors computed using 1000 MCMC chains.**


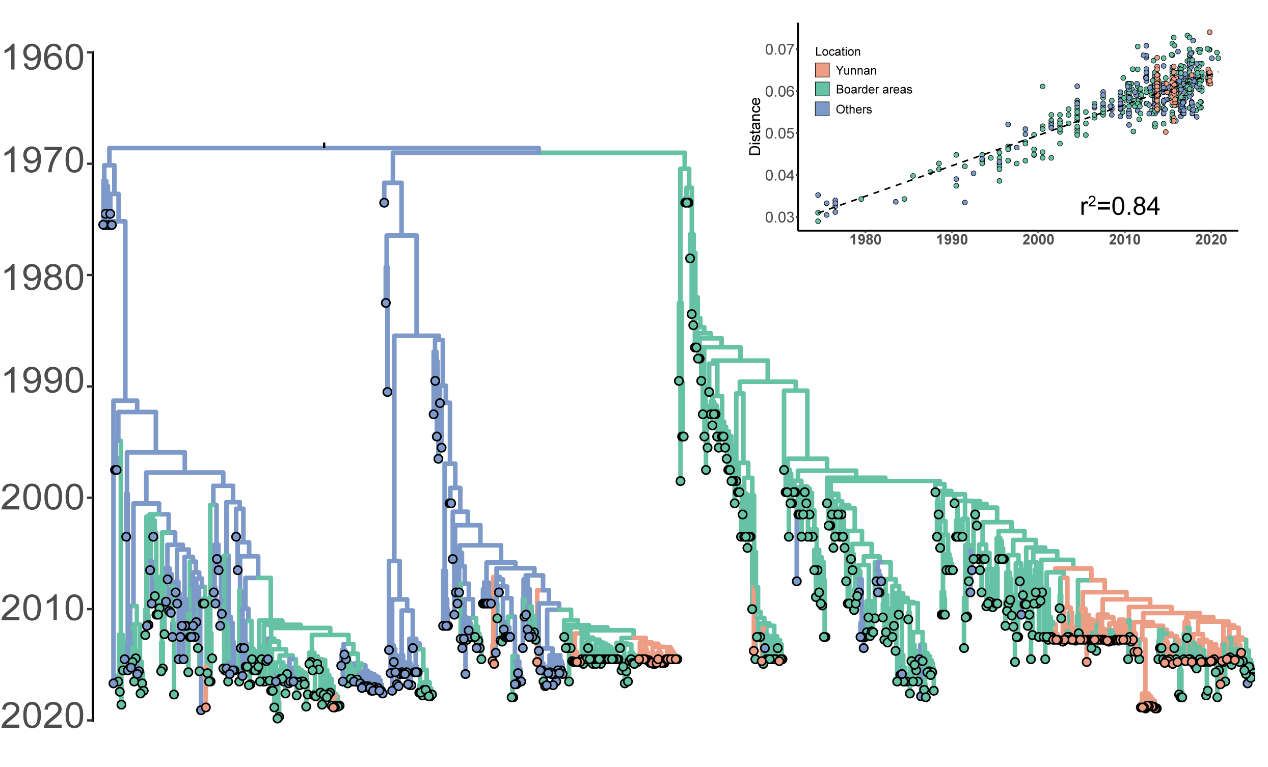


**Fig. S6** **Maximum clade credibility trees of E gene of DENV-2 (n=725).** Branches and tips are colored according to Yunnan (orange), the border areas of Yunnan (green) and other areas (blue). The inset shows a root-to-tip regression of genetic divergence against dates of sample collection.


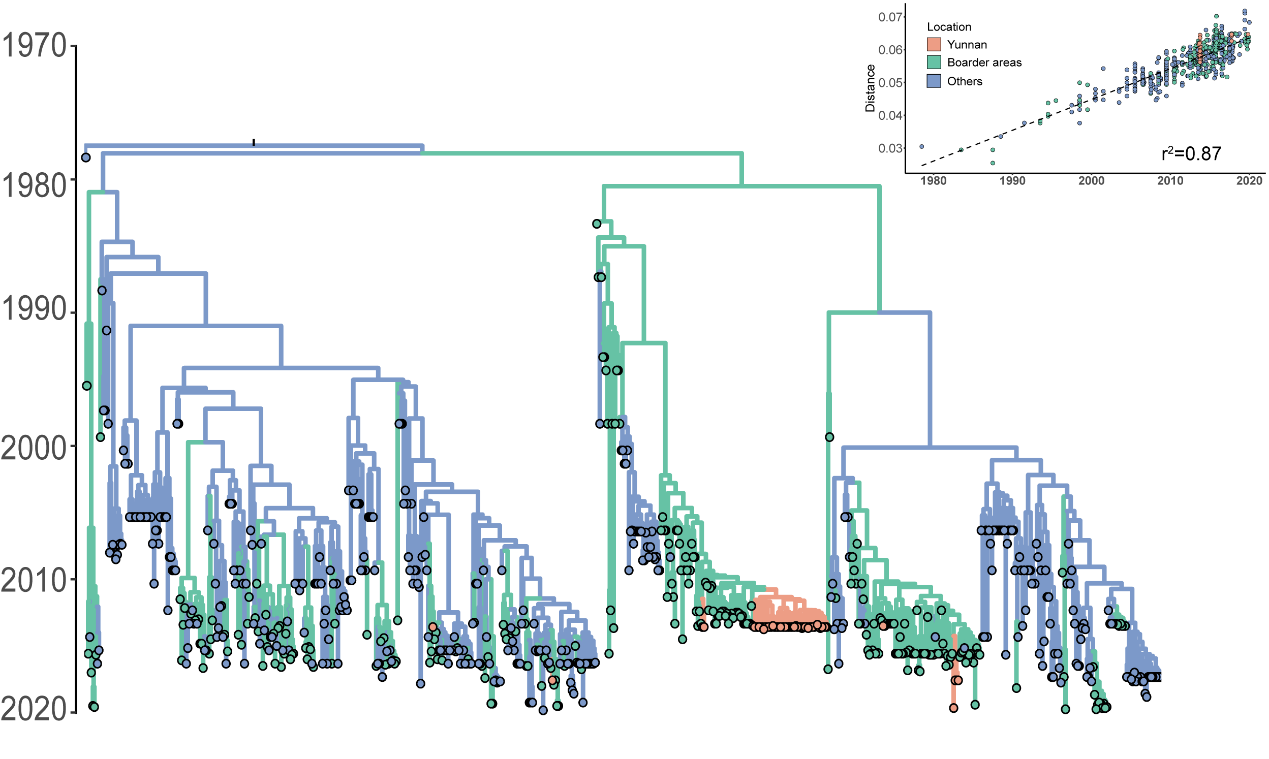


**Fig. S7** **Maximum clade credibility trees of E gene of DENV-3 (n=704).** Branches and tips are colored according to Yunnan (orange), the border areas of Yunnan (green) and other areas (blue). The inset shows a root-to-tip regression of genetic divergence against dates of sample collection.


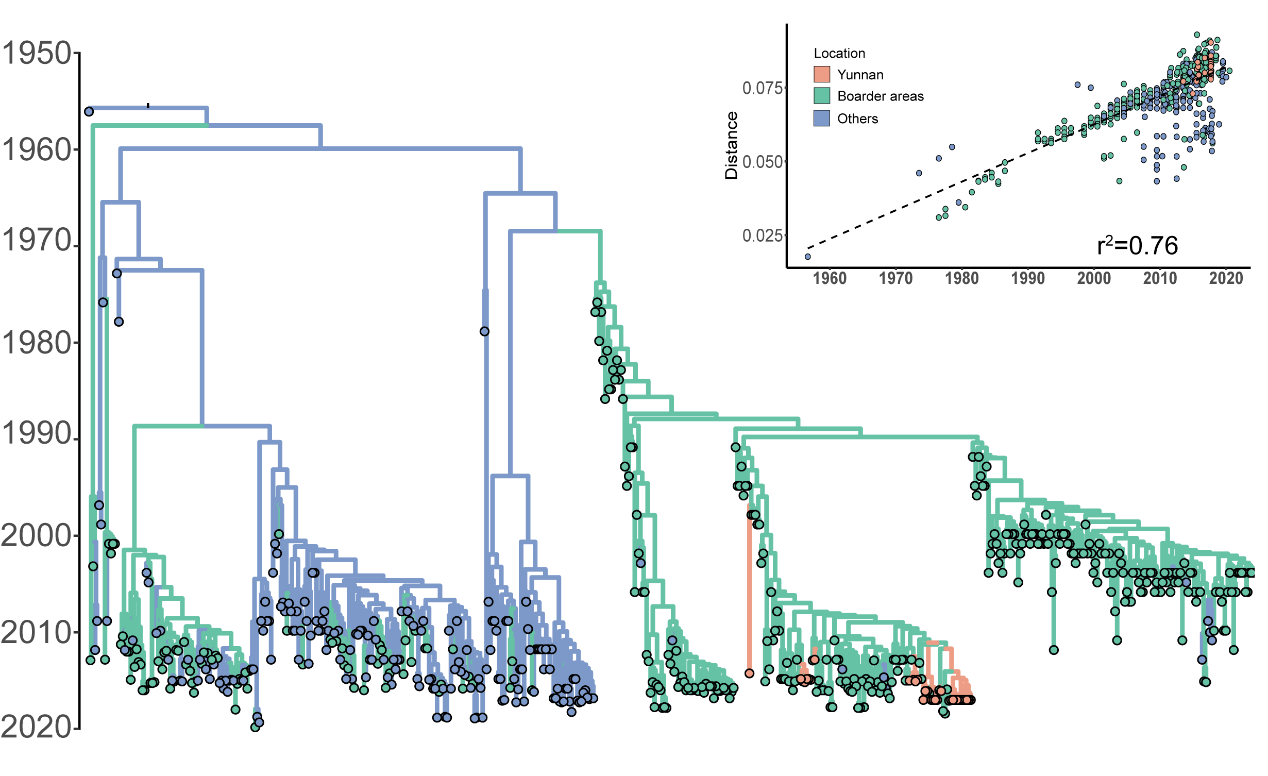


**Fig. S8** **Maximum clade credibility trees of E gene of DENV-4 (n=591).** Branches and tips are colored according to Yunnan (orange), the border areas of Yunnan (green) and other areas (blue). The inset shows a root-to-tip regression of genetic divergence against dates of sample collection.

**
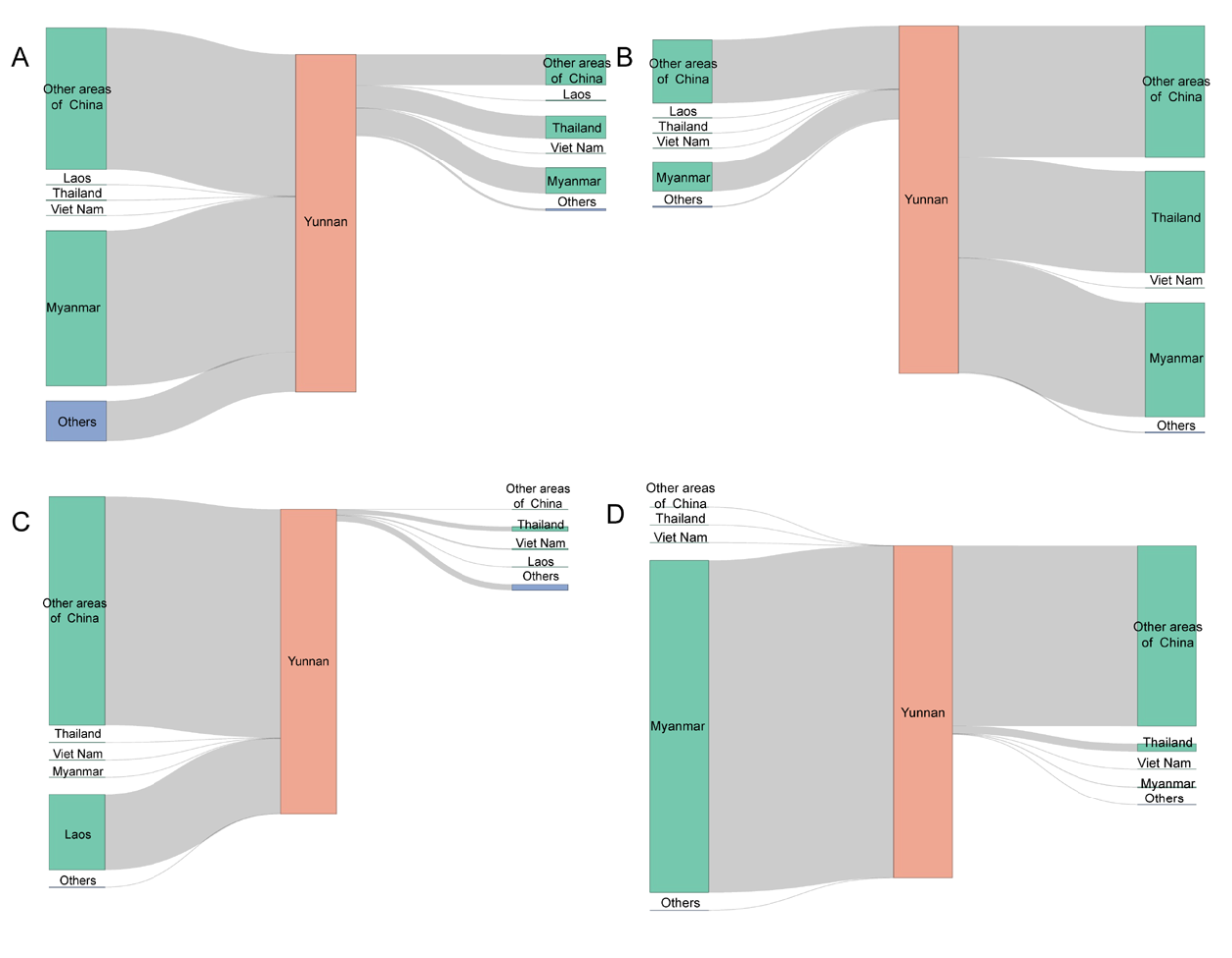
**

**Fig. S9 Relative risk of dengue virus introduction.** Flows are proportional to number of expected transitions from a given origin to Yunnan, and then to a specific destination of (A) DENV-1, (B) DENV-2, (C) DENV-3, and (D) DENV-4.


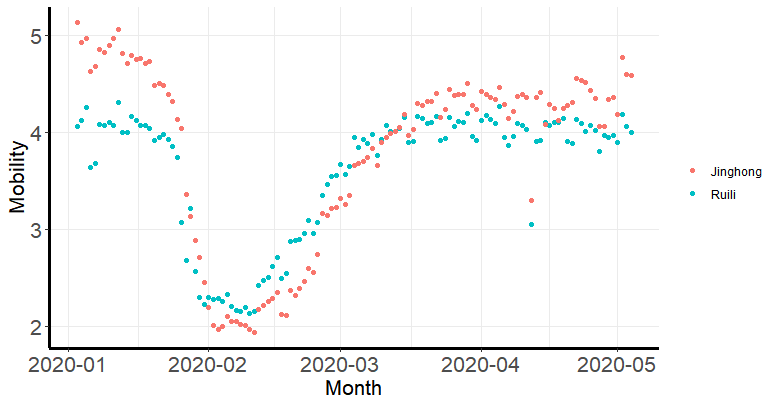


**Fig. S10 Intra-city movement in Ruili and Jinghong.**

**
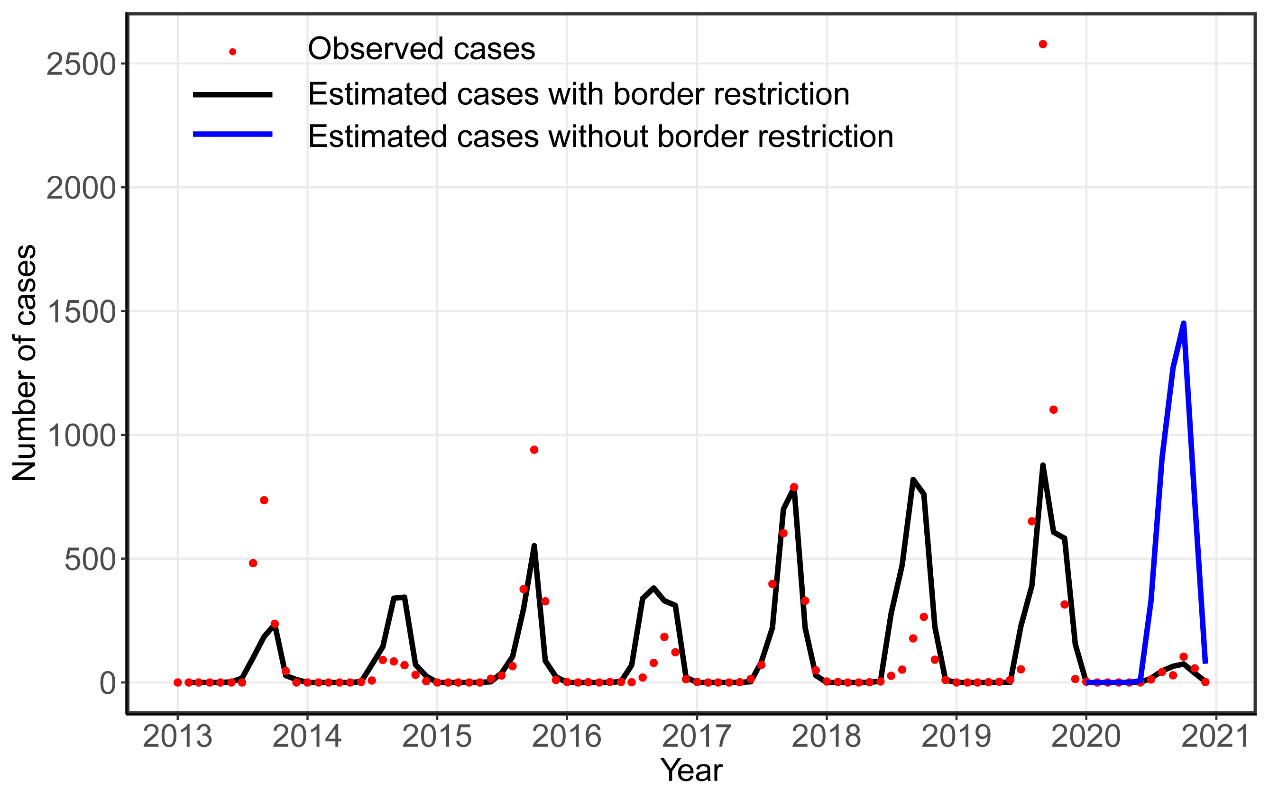
**

**Fig. S11 Time series plot of observed and estimated dengue cases from 2013 to 2020 in Yunnan using the final fitted GLM in our study.**

**Table S1 DENV E gene sequences used in study.**

| Accession number | Location | Year | Serotype |
| --- | --- | --- | --- |
| MN444623 | Laos | 2020 | DENV1 |
| MN894253 | China-Jiangxi | 2019 | DENV1 |
| MN923106 | China-Guangdong | 2020 | DENV1 |
| MN894256 | China-Jiangxi | 2020 | DENV1 |
| MN933746 | China-Guangdong | 2019 | DENV1 |
| MN869908 | China-Guangdong | 2019 | DENV1 |
| MN869907 | China-Guangdong | 2019 | DENV1 |
| MN933666 | China-Guangdong | 2019 | DENV1 |
| MK517741 | China-Guangdong | 2019 | DENV1 |
| MN913514 | China-Guangdong | 2019 | DENV1 |
| MN913516 | China-Guangdong | 2019 | DENV1 |
| MK517740 | China-Guangdong | 2019 | DENV1 |
| MK517742 | China-Guangdong | 2019 | DENV1 |
| MN913517 | China-Guangdong | 2019 | DENV1 |
| MN913519 | China-Guangdong | 2019 | DENV1 |
| MN894185 | China-Jiangxi | 2019 | DENV1 |
| MN933766 | China-Guangdong | 2019 | DENV1 |
| MK529730 | Cambodia | 2019 | DENV1 |
| MN921340 | China-Guangdong | 2020 | DENV1 |
| MT447150 | China-Hainan | 2020 | DENV1 |
| MN921479 | China-Guangdong | 2020 | DENV1 |
| MN894251 | China-Jiangxi | 2020 | DENV1 |
| MK203772 | China-Jiangsu | 2019 | DENV1 |
| MK203780 | China-Jiangsu | 2019 | DENV1 |
| MK203779 | China-Jiangsu | 2019 | DENV1 |
| MN453626 | China-Zhejiang | 2019 | DENV1 |
| MK203786 | China-Jiangsu | 2019 | DENV1 |
| MK203783 | China-Jiangsu | 2019 | DENV1 |
| MN933764 | China-Guangdong | 2019 | DENV1 |
| MK517731 | China-Guangdong | 2019 | DENV1 |
| MK517732 | China-Guangdong | 2019 | DENV1 |
| MN933751 | China-Guangdong | 2019 | DENV1 |
| MN933756 | China-Guangdong | 2019 | DENV1 |
| MW014048 | China-Guangdong | 2019 | DENV1 |
| MN933750 | China-Guangdong | 2019 | DENV1 |
| MN933743 | China-Guangdong | 2019 | DENV1 |
| MN933741 | China-Guangdong | 2019 | DENV1 |
| MN869912 | China-Guangdong | 2019 | DENV1 |
| MN913518 | China-Guangdong | 2019 | DENV1 |
| MK517729 | China-Guangdong | 2019 | DENV1 |
| MN933754 | China-Guangdong | 2019 | DENV1 |
| MK517728 | China-Guangdong | 2019 | DENV1 |
| MN933758 | China-Guangdong | 2019 | DENV1 |
| MN955650 | Thailand | 2019 | DENV1 |
| MN955660 | Thailand | 2019 | DENV1 |
| MN913521 | China-Guangdong | 2020 | DENV1 |
| MN933718 | China-Guangdong | 2019 | DENV1 |
| MN921461 | China-Guangdong | 2020 | DENV1 |
| MN921278 | China-Guangdong | 2020 | DENV1 |
| MN923098 | China-Guangdong | 2019 | DENV1 |
| MN955621 | Thailand | 2018 | DENV1 |
| MN955651 | Thailand | 2019 | DENV1 |
| MN923078 | China-Guangdong | 2020 | DENV1 |
| MN921435 | China-Guangdong | 2020 | DENV1 |
| MN955622 | Thailand | 2018 | DENV1 |
| MN955659 | Thailand | 2019 | DENV1 |
| MG564080 | Thailand | 2016 | DENV1 |
| MG894863 | China-Taiwan | 2016 | DENV1 |
| MN933679 | China-Guangdong | 2018 | DENV1 |
| MN933684 | China-Guangdong | 2018 | DENV1 |
| MN933690 | China-Guangdong | 2018 | DENV1 |
| MT804543 | China-Guangdong | 2018 | DENV1 |
| MT804544 | China-Guangdong | 2018 | DENV1 |
| MT804545 | China-Guangdong | 2018 | DENV1 |
| MT804541 | China-Guangdong | 2018 | DENV1 |
| MT804537 | China-Guangdong | 2018 | DENV1 |
| MT804539 | China-Guangdong | 2018 | DENV1 |
| MN933664 | China-Guangdong | 2018 | DENV1 |
| MN933675 | China-Guangdong | 2018 | DENV1 |
| MN018291 | China-Guangdong | 2017 | DENV1 |
| MF033260 | Singapore | 2017 | DENV1 |
| MK466343 | China-Yunnan | 2018 | DENV1 |
| MK466344 | China-Yunnan | 2018 | DENV1 |
| MK466345 | China-Yunnan | 2018 | DENV1 |
| MK466342 | China-Yunnan | 2018 | DENV1 |
| MK529738 | China-Yunnan | 2019 | DENV1 |
| MK529757 | China-Yunnan | 2019 | DENV1 |
| MK529734 | China-Yunnan | 2019 | DENV1 |
| MK529729 | China-Yunnan | 2019 | DENV1 |
| MN639767 | China-Yunnan | 2019 | DENV1 |
| MK529754 | China-Yunnan | 2019 | DENV1 |
| MN639764 | China-Yunnan | 2019 | DENV1 |
| MN639766 | China-Yunnan | 2019 | DENV1 |
| MN955623 | Thailand | 2018 | DENV1 |
| MN955648 | Thailand | 2019 | DENV1 |
| MG894896 | China-Taiwan | 2016 | DENV1 |
| MN933676 | China-Guangdong | 2018 | DENV1 |
| MN933737 | China-Guangdong | 2019 | DENV1 |
| MN933739 | China-Guangdong | 2019 | DENV1 |
| MN886829 | China-Guangdong | 2018 | DENV1 |
| MN018306 | China-Guangdong | 2018 | DENV1 |
| MN933672 | China-Guangdong | 2018 | DENV1 |
| MK566225 | China-Guangdong | 2018 | DENV1 |
| MN933685 | China-Guangdong | 2018 | DENV1 |
| MK566223 | China-Guangdong | 2018 | DENV1 |
| MK566220 | China-Guangdong | 2018 | DENV1 |
| MN886837 | China-Guangdong | 2018 | DENV1 |
| MH010601 | China-Zhejiang | 2018 | DENV1 |
| MN886832 | China-Guangdong | 2018 | DENV1 |
| MN886835 | China-Guangdong | 2018 | DENV1 |
| MN886833 | China-Guangdong | 2018 | DENV1 |
| KU509291 | Thailand | 2014 | DENV1 |
| JF967876 | Malaysia | 2010 | DENV1 |
| JF967796 | Malaysia | 2009 | DENV1 |
| KX224262 | Singapore | 2015 | DENV1 |
| MH680454 | Singapore | 2015 | DENV1 |
| MF033233 | Singapore | 2015 | DENV1 |
| MH680472 | Singapore | 2015 | DENV1 |
| MH680481 | Singapore | 2016 | DENV1 |
| MH680389 | Singapore | 2015 | DENV1 |
| KX224260 | Singapore | 2015 | DENV1 |
| KU509292 | Viet Nam | 2015 | DENV1 |
| KX452053 | Malaysia | 2014 | DENV1 |
| MH680447 | Singapore | 2015 | DENV1 |
| MF033214 | Singapore | 2014 | DENV1 |
| MG894774 | China-Taiwan | 2014 | DENV1 |
| KJ806845 | Malaysia | 2014 | DENV1 |
| KJ806823 | Malaysia | 2014 | DENV1 |
| KJ806822 | Malaysia | 2014 | DENV1 |
| KJ806961 | Singapore | 2015 | DENV1 |
| KJ806825 | Malaysia | 2014 | DENV1 |
| KJ806824 | Malaysia | 2014 | DENV1 |
| KJ806949 | Singapore | 2014 | DENV1 |
| MG894836 | China-Taiwan | 2015 | DENV1 |
| JQ048541 | China-Guangdong | 2012 | DENV1 |
| KJ806828 | Malaysia | 2014 | DENV1 |
| KJ806817 | Malaysia | 2013 | DENV1 |
| KJ806826 | Malaysia | 2014 | DENV1 |
| MG894727 | China-Taiwan | 2013 | DENV1 |
| KJ806827 | Malaysia | 2014 | DENV1 |
| KJ806831 | Malaysia | 2014 | DENV1 |
| MG894681 | China-Taiwan | 2012 | DENV1 |
| MG894703 | China-Taiwan | 2013 | DENV1 |
| JF967920 | Malaysia | 2011 | DENV1 |
| MG894674 | China-Taiwan | 2011 | DENV1 |
| JF967911 | Malaysia | 2011 | DENV1 |
| JF967887 | Malaysia | 2010 | DENV1 |
| JN415512 | Malaysia | 2009 | DENV1 |
| MN933722 | China-Guangdong | 2019 | DENV1 |
| MF381076 | China-Guangdong | 2016 | DENV1 |
| KT825034 | Indonesia | 2016 | DENV1 |
| KT825037 | Malaysia | 2016 | DENV1 |
| MT705040 | China-Yunnan | 2020 | DENV1 |
| MK466347 | China-Yunnan | 2018 | DENV1 |
| MG894873 | China-Taiwan | 2016 | DENV1 |
| MT705031 | China-Yunnan | 2020 | DENV1 |
| MT705113 | China-Yunnan | 2020 | DENV1 |
| MT705019 | China-Yunnan | 2020 | DENV1 |
| MT705020 | China-Yunnan | 2020 | DENV1 |
| KT819303 | China-Guangdong | 2015 | DENV1 |
| MN018299 | China-Guangdong | 2015 | DENV1 |
| MG894890 | China-Taiwan | 2016 | DENV1 |
| MG894937 | China-Taiwan | 2017 | DENV1 |
| MG894926 | China-Taiwan | 2017 | DENV1 |
| MG894934 | China-Taiwan | 2017 | DENV1 |
| MH680521 | Singapore | 2017 | DENV1 |
| MH680523 | Singapore | 2017 | DENV1 |
| MH680524 | Singapore | 2017 | DENV1 |
| KT825038 | Malaysia | 2016 | DENV1 |
| MH680473 | Singapore | 2015 | DENV1 |
| MG894858 | China-Taiwan | 2016 | DENV1 |
| MH680495 | Singapore | 2016 | DENV1 |
| KP055772 | China-Guangdong | 2015 | DENV1 |
| KX621250 | China-Guangdong | 2015 | DENV1 |
| KP772252 | China-Hubei | 2015 | DENV1 |
| MG894883 | China-Taiwan | 2016 | DENV1 |
| MH680493 | Singapore | 2016 | DENV1 |
| MH680516 | Singapore | 2016 | DENV1 |
| MN018289 | China-Guangdong | 2017 | DENV1 |
| MN018305 | China-Guangdong | 2017 | DENV1 |
| MF033232 | Singapore | 2015 | DENV1 |
| KU291794 | China-Guangdong | 2015 | DENV1 |
| MN886882 | China-Guangdong | 2015 | DENV1 |
| KT037108 | China-Guangdong | 2015 | DENV1 |
| KP191523 | China-Guangdong | 2015 | DENV1 |
| MT804523 | China-Guangdong | 2015 | DENV1 |
| KP191521 | China-Guangdong | 2015 | DENV1 |
| KX225492 | China-Guangdong | 2016 | DENV1 |
| MH680490 | Singapore | 2016 | DENV1 |
| MH729964 | China-Fujian | 2017 | DENV1 |
| MG894928 | China-Taiwan | 2017 | DENV1 |
| MK566207 | China-Guangdong | 2017 | DENV1 |
| MK566210 | China-Guangdong | 2017 | DENV1 |
| MK566204 | China-Guangdong | 2017 | DENV1 |
| MK566212 | China-Guangdong | 2017 | DENV1 |
| MN933704 | China-Guangdong | 2017 | DENV1 |
| MK566211 | China-Guangdong | 2017 | DENV1 |
| MK566208 | China-Guangdong | 2017 | DENV1 |
| MN018317 | China-Guangdong | 2017 | DENV1 |
| MN933655 | China-Guangdong | 2015 | DENV1 |
| KT428609 | China-Guangdong | 2015 | DENV1 |
| MG560230 | China-Guangdong | 2015 | DENV1 |
| MN886893 | China-Guangdong | 2015 | DENV1 |
| KY886978 | China-Zhejiang | 2017 | DENV1 |
| MG894803 | China-Taiwan | 2014 | DENV1 |
| MG894915 | China-Taiwan | 2016 | DENV1 |
| KT825039 | Myanmar | 2016 | DENV1 |
| KT825069 | Malaysia | 2016 | DENV1 |
| MF381077 | China-Guangdong | 2016 | DENV1 |
| MG894887 | China-Taiwan | 2016 | DENV1 |
| MG894889 | China-Taiwan | 2016 | DENV1 |
| MG894875 | China-Taiwan | 2016 | DENV1 |
| MG894899 | China-Taiwan | 2016 | DENV1 |
| MG894853 | China-Taiwan | 2015 | DENV1 |
| MG894901 | China-Taiwan | 2016 | DENV1 |
| MG894897 | China-Taiwan | 2016 | DENV1 |
| KJ806959 | Singapore | 2015 | DENV1 |
| KJ806873 | Malaysia | 2015 | DENV1 |
| MG894865 | China-Taiwan | 2016 | DENV1 |
| KJ806847 | Malaysia | 2014 | DENV1 |
| KJ806872 | Malaysia | 2015 | DENV1 |
| MG933822 | China-Yunnan | 2018 | DENV1 |
| MH680468 | Singapore | 2015 | DENV1 |
| MH680496 | Singapore | 2016 | DENV1 |
| MN018314 | China-Guangdong | 2016 | DENV1 |
| KX452067 | Malaysia | 2014 | DENV1 |
| MN933661 | China-Guangdong | 2017 | DENV1 |
| MN933663 | China-Guangdong | 2017 | DENV1 |
| MF033246 | Singapore | 2016 | DENV1 |
| KT175084 | Malaysia | 2015 | DENV1 |
| MG894809 | China-Taiwan | 2014 | DENV1 |
| KX224261 | Singapore | 2015 | DENV1 |
| MH680504 | Singapore | 2016 | DENV1 |
| KU666939 | Malaysia | 2014 | DENV1 |
| KT232186 | China-Guangdong | 2015 | DENV1 |
| KT825000 | Indonesia | 2014 | DENV1 |
| MG894911 | China-Taiwan | 2016 | DENV1 |
| MN933720 | China-Guangdong | 2019 | DENV1 |
| MG894759 | China-Taiwan | 2014 | DENV1 |
| KY216156 | Indonesia | 2015 | DENV1 |
| MN018323 | China-Guangdong | 2016 | DENV1 |
| MN923100 | China-Guangdong | 2019 | DENV1 |
| MT377728 | Indonesia | 2020 | DENV1 |
| MK629470 | Indonesia | 2014 | DENV1 |
| MH036385 | Indonesia | 2017 | DENV1 |
| MK629469 | Indonesia | 2015 | DENV1 |
| MT078651 | China-Zhejiang | 2020 | DENV1 |
| MN933726 | China-Guangdong | 2019 | DENV1 |
| JF960215 | Singapore | 2010 | DENV1 |
| JF967821 | Malaysia | 2009 | DENV1 |
| JF960218 | Singapore | 2010 | DENV1 |
| JF960216 | Singapore | 2010 | DENV1 |
| GQ357687 | Singapore | 2009 | DENV1 |
| JF967839 | Malaysia | 2009 | DENV1 |
| MH679958 | Singapore | 2013 | DENV1 |
| JF960223 | Singapore | 2011 | DENV1 |
| KJ806838 | Malaysia | 2014 | DENV1 |
| JF967847 | Malaysia | 2009 | DENV1 |
| KU529702 | Indonesia | 2016 | DENV1 |
| MG894964 | China-Taiwan | 2017 | DENV1 |
| MG894752 | China-Taiwan | 2013 | DENV1 |
| MG894846 | China-Taiwan | 2015 | DENV1 |
| KX646378 | Indonesia | 2010 | DENV1 |
| JF967877 | Indonesia | 2010 | DENV1 |
| KM216674 | Indonesia | 2011 | DENV1 |
| KX646379 | Indonesia | 2010 | DENV1 |
| KY216157 | Indonesia | 2013 | DENV1 |
| KY216158 | Indonesia | 2013 | DENV1 |
| KY216159 | Indonesia | 2013 | DENV1 |
| KU529727 | Indonesia | 2016 | DENV1 |
| JN415511 | Malaysia | 2006 | DENV1 |
| EU081277 | Singapore | 2006 | DENV1 |
| MK517738 | China-Guangdong | 2019 | DENV1 |
| MK517733 | China-Guangdong | 2019 | DENV1 |
| MN933736 | China-Guangdong | 2019 | DENV1 |
| MN018292 | China-Guangdong | 2018 | DENV1 |
| MN955646 | Thailand | 2019 | DENV1 |
| MN018329 | China-Guangdong | 2016 | DENV1 |
| MN018330 | China-Guangdong | 2016 | DENV1 |
| MN923091 | China-Guangdong | 2020 | DENV1 |
| MN018336 | China-Guangdong | 2017 | DENV1 |
| MN018285 | China-Guangdong | 2016 | DENV1 |
| MK780859 | Thailand | 2017 | DENV1 |
| MK629464 | Indonesia | 2015 | DENV1 |
| MN018304 | China-Guangdong | 2015 | DENV1 |
| MN018288 | China-Guangdong | 2016 | DENV1 |
| MN018315 | China-Guangdong | 2016 | DENV1 |
| MN018316 | China-Guangdong | 2016 | DENV1 |
| MN933660 | China-Guangdong | 2016 | DENV1 |
| MK629463 | Indonesia | 2014 | DENV1 |
| MN018293 | China-Guangdong | 2014 | DENV1 |
| MH036379 | Indonesia | 2016 | DENV1 |
| MH036380 | Indonesia | 2016 | DENV1 |
| MH036387 | Indonesia | 2017 | DENV1 |
| MH036381 | Indonesia | 2017 | DENV1 |
| MH036386 | Indonesia | 2017 | DENV1 |
| MH036384 | Indonesia | 2017 | DENV1 |
| MH036382 | Indonesia | 2017 | DENV1 |
| MH036375 | Indonesia | 2017 | DENV1 |
| MH036377 | Indonesia | 2017 | DENV1 |
| MK629460 | Indonesia | 2015 | DENV1 |
| MN018335 | China-Guangdong | 2016 | DENV1 |
| MH178411 | Indonesia | 2017 | DENV1 |
| MH823207 | Indonesia | 2016 | DENV1 |
| MK629471 | Indonesia | 2016 | DENV1 |
| MK629462 | Indonesia | 2016 | DENV1 |
| MN018321 | China-Guangdong | 2015 | DENV1 |
| MK629466 | Indonesia | 2016 | DENV1 |
| MK629467 | Indonesia | 2017 | DENV1 |
| MK629465 | Indonesia | 2016 | DENV1 |
| KY586362 | Thailand | 2002 | DENV1 |
| MT804440 | China-Guangdong | 2014 | DENV1 |
| MT804458 | China-Guangdong | 2014 | DENV1 |
| MT804456 | China-Guangdong | 2014 | DENV1 |
| MT804455 | China-Guangdong | 2014 | DENV1 |
| MT804457 | China-Guangdong | 2014 | DENV1 |
| MT804433 | China-Guangdong | 2014 | DENV1 |
| MT804453 | China-Guangdong | 2014 | DENV1 |
| MT804452 | China-Guangdong | 2014 | DENV1 |
| MT804450 | China-Guangdong | 2014 | DENV1 |
| MT804449 | China-Guangdong | 2014 | DENV1 |
| MT804451 | China-Guangdong | 2014 | DENV1 |
| MN886913 | China-Guangdong | 2014 | DENV1 |
| MN886909 | China-Guangdong | 2014 | DENV1 |
| MN886900 | China-Guangdong | 2014 | DENV1 |
| MN886911 | China-Guangdong | 2014 | DENV1 |
| MT804432 | China-Guangdong | 2014 | DENV1 |
| MN886886 | China-Guangdong | 2015 | DENV1 |
| MN886889 | China-Guangdong | 2015 | DENV1 |
| MN018295 | China-Guangdong | 2015 | DENV1 |
| MN886897 | China-Guangdong | 2015 | DENV1 |
| MT804524 | China-Guangdong | 2015 | DENV1 |
| MN886890 | China-Guangdong | 2015 | DENV1 |
| MN512234 | China-Guangxi | 2015 | DENV1 |
| MN933654 | China-Guangdong | 2015 | DENV1 |
| MK780858 | Thailand | 2017 | DENV1 |
| MK629468 | Indonesia | 2016 | DENV1 |
| MN018312 | China-Guangdong | 2016 | DENV1 |
| MN018296 | China-Guangdong | 2016 | DENV1 |
| KP191499 | China-Guangdong | 2015 | DENV1 |
| KX620454 | China-Guangdong | 2015 | DENV1 |
| MN512233 | China-Guangxi | 2015 | DENV1 |
| MF033222 | Singapore | 2015 | DENV1 |
| MG564082 | Thailand | 2016 | DENV1 |
| KU291674 | China-Guangdong | 2014 | DENV1 |
| KU509290 | Thailand | 2014 | DENV1 |
| KJ545453 | China-Guangdong | 2014 | DENV1 |
| KY672933 | China-Yunnan | 2016 | DENV1 |
| KT175079 | China-Taiwan | 2014 | DENV1 |
| KR051929 | Myanmar | 2014 | DENV1 |
| KJ806840 | Malaysia | 2014 | DENV1 |
| MH680136 | Singapore | 2014 | DENV1 |
| KR051930 | Myanmar | 2014 | DENV1 |
| KJ939369 | China-Yunnan | 2014 | DENV1 |
| KX357943 | Myanmar | 2016 | DENV1 |
| KT175103 | Myanmar | 2012 | DENV1 |
| KU509294 | Thailand | 2015 | DENV1 |
| MG894862 | China-Taiwan | 2016 | DENV1 |
| MN933734 | China-Guangdong | 2019 | DENV1 |
| JF967840 | Myanmar | 2009 | DENV1 |
| AY732386 | Thailand | 2003 | DENV1 |
| KY586310 | Thailand | 2002 | DENV1 |
| KY586309 | Thailand | 2002 | DENV1 |
| KY586306 | Thailand | 2001 | DENV1 |
| AY620946 | Myanmar | 2002 | DENV1 |
| KY586308 | Thailand | 2001 | DENV1 |
| KY586311 | Thailand | 2007 | DENV1 |
| KY038895 | China-Yunnan | 2015 | DENV1 |
| MN933656 | China-Guangdong | 2015 | DENV1 |
| KY672937 | China-Yunnan | 2015 | DENV1 |
| KY672938 | China-Yunnan | 2015 | DENV1 |
| KY038893 | China-Yunnan | 2015 | DENV1 |
| KX056451 | China-Yunnan | 2015 | DENV1 |
| KX056452 | China-Yunnan | 2015 | DENV1 |
| KX056448 | China-Yunnan | 2015 | DENV1 |
| KJ806849 | Malaysia | 2014 | DENV1 |
| KY234170 | Thailand | 2016 | DENV1 |
| MF598855 | China-Yunnan | 2017 | DENV1 |
| KF559255 | Myanmar | 2006 | DENV1 |
| KT452791 | Myanmar | 2006 | DENV1 |
| KT175077 | China-Taiwan | 2012 | DENV1 |
| KR051909 | Myanmar | 2014 | DENV1 |
| KR051908 | Myanmar | 2014 | DENV1 |
| KX357952 | Myanmar | 2016 | DENV1 |
| KX357962 | Myanmar | 2016 | DENV1 |
| JF967822 | Thailand | 2009 | DENV1 |
| KT175102 | Myanmar | 2008 | DENV1 |
| JF967848 | Myanmar | 2009 | DENV1 |
| AY726553 | Myanmar | 2003 | DENV1 |
| AY618211 | Myanmar | 2002 | DENV1 |
| AY726551 | Myanmar | 2002 | DENV1 |
| AY726549 | Myanmar | 2002 | DENV1 |
| KF559253 | Myanmar | 2003 | DENV1 |
| AY620950 | Myanmar | 2000 | DENV1 |
| AY713476 | Myanmar | 2002 | DENV1 |
| AY620953 | Myanmar | 2002 | DENV1 |
| AY620949 | Myanmar | 2002 | DENV1 |
| AY620951 | Myanmar | 2001 | DENV1 |
| AY620948 | Myanmar | 2002 | DENV1 |
| AY620952 | Myanmar | 2001 | DENV1 |
| AY618880 | Myanmar | 2001 | DENV1 |
| AY726554 | Myanmar | 1999 | DENV1 |
| AY732444 | Thailand | 1998 | DENV1 |
| MN921385 | China-Guangdong | 2020 | DENV1 |
| MN913529 | China-Guangdong | 2020 | DENV1 |
| MW014042 | Cambodia | 2020 | DENV1 |
| MN921640 | China-Guangdong | 2020 | DENV1 |
| MN923093 | China-Guangdong | 2020 | DENV1 |
| MN921503 | China-Guangdong | 2020 | DENV1 |
| MN923087 | China-Guangdong | 2020 | DENV1 |
| MN921276 | China-Guangdong | 2020 | DENV1 |
| MN921458 | China-Guangdong | 2020 | DENV1 |
| MN894252 | China-Jiangxi | 2020 | DENV1 |
| MN945986 | China-Guangdong | 2020 | DENV1 |
| MT705021 | China-Yunnan | 2020 | DENV1 |
| LC428054 | Vietnam | 2018 | DENV1 |
| LC428080 | Vietnam | 2018 | DENV1 |
| MN921518 | China-Guangdong | 2020 | DENV1 |
| LC428079 | Vietnam | 2018 | DENV1 |
| MN933760 | China-Guangdong | 2019 | DENV1 |
| MN653528 | China-China-Henan | 2020 | DENV1 |
| MN933761 | China-Guangdong | 2019 | DENV1 |
| MN921639 | China-Guangdong | 2020 | DENV1 |
| MN921441 | China-Guangdong | 2020 | DENV1 |
| MN894250 | China-Jiangxi | 2020 | DENV1 |
| MN921265 | China-Guangdong | 2020 | DENV1 |
| MN921287 | China-Guangdong | 2020 | DENV1 |
| MN921642 | China-Guangdong | 2020 | DENV1 |
| MN921297 | China-Guangdong | 2020 | DENV1 |
| MW014036 | China-Guangdong | 2020 | DENV1 |
| MN913520 | China-Guangdong | 2019 | DENV1 |
| MN894229 | China-Jiangxi | 2020 | DENV1 |
| MN921259 | China-Guangdong | 2020 | DENV1 |
| MT705036 | China-Yunnan | 2020 | DENV1 |
| MN921274 | China-Guangdong | 2020 | DENV1 |
| MT705018 | China-Yunnan | 2020 | DENV1 |
| MW228041 | Cambodia | 2020 | DENV1 |
| MN921419 | China-Guangdong | 2020 | DENV1 |
| MN921289 | China-Guangdong | 2020 | DENV1 |
| MN921258 | China-Guangdong | 2020 | DENV1 |
| MN921459 | China-Guangdong | 2020 | DENV1 |
| MW014035 | Cambodia | 2020 | DENV1 |
| MT705028 | China-Yunnan | 2020 | DENV1 |
| MK905537 | China-Henan | 2019 | DENV1 |
| MN923099 | China-Guangdong | 2019 | DENV1 |
| MW014040 | Cambodia | 2019 | DENV1 |
| MN923079 | China-Guangdong | 2020 | DENV1 |
| MN921361 | China-Guangdong | 2020 | DENV1 |
| MW014046 | Cambodia | 2019 | DENV1 |
| MN921266 | China-Guangdong | 2020 | DENV1 |
| MW014044 | China-Guangdong | 2020 | DENV1 |
| MN921467 | China-Guangdong | 2020 | DENV1 |
| MT705046 | China-Yunnan | 2020 | DENV1 |
| MT705135 | China-Yunnan | 2020 | DENV1 |
| MT705025 | China-Yunnan | 2020 | DENV1 |
| MN894239 | China-Jiangxi | 2020 | DENV1 |
| MN921432 | China-Guangdong | 2020 | DENV1 |
| MW228042 | Laos | 2020 | DENV1 |
| MT705024 | China-Yunnan | 2020 | DENV1 |
| MT705120 | China-Yunnan | 2020 | DENV1 |
| MT705124 | China-Yunnan | 2020 | DENV1 |
| MN921634 | China-Guangdong | 2020 | DENV1 |
| MT705039 | China-Yunnan | 2020 | DENV1 |
| MT705133 | China-Yunnan | 2020 | DENV1 |
| MT705038 | China-Yunnan | 2020 | DENV1 |
| MN653548 | China-Henan | 2020 | DENV1 |
| MN921313 | China-Guangdong | 2020 | DENV1 |
| MN921533 | China-Guangdong | 2020 | DENV1 |
| MN921341 | China-Guangdong | 2020 | DENV1 |
| MN894265 | China-Jiangxi | 2020 | DENV1 |
| MN921422 | China-Guangdong | 2020 | DENV1 |
| MN894192 | China-Jiangxi | 2020 | DENV1 |
| MW314721 | Cambodia | 2020 | DENV1 |
| MN921272 | China-Guangdong | 2020 | DENV1 |
| MN921444 | China-Guangdong | 2020 | DENV1 |
| MN653534 | China-Henan | 2020 | DENV1 |
| MN933727 | China-Guangdong | 2019 | DENV1 |
| MT705147 | China-Yunnan | 2020 | DENV1 |
| MN894264 | China-Jiangxi | 2020 | DENV1 |
| MN921492 | China-Guangdong | 2020 | DENV1 |
| MN894213 | China-Jiangxi | 2020 | DENV1 |
| MN894221 | China-Jiangxi | 2020 | DENV1 |
| MN894191 | China-Jiangxi | 2020 | DENV1 |
| MN894218 | China-Jiangxi | 2020 | DENV1 |
| MW014034 | China-Guangdong | 2020 | DENV1 |
| MN921462 | China-Guangdong | 2020 | DENV1 |
| MN921506 | China-Guangdong | 2020 | DENV1 |
| MN894243 | China-Jiangxi | 2020 | DENV1 |
| MN921550 | China-Guangdong | 2020 | DENV1 |
| MN921449 | China-Guangdong | 2020 | DENV1 |
| MN921305 | China-Guangdong | 2020 | DENV1 |
| MT705034 | China-Yunnan | 2020 | DENV1 |
| MN921483 | China-Guangdong | 2020 | DENV1 |
| MN923097 | China-Guangdong | 2020 | DENV1 |
| MN921285 | China-Guangdong | 2020 | DENV1 |
| MT705042 | China-Yunnan | 2020 | DENV1 |
| MN894246 | China-Jiangxi | 2020 | DENV1 |
| MN894238 | China-Jiangxi | 2020 | DENV1 |
| MN894267 | China-Jiangxi | 2020 | DENV1 |
| MN894233 | China-Jiangxi | 2020 | DENV1 |
| MN894237 | China-Jiangxi | 2020 | DENV1 |
| MN921552 | China-Guangdong | 2020 | DENV1 |
| MN921516 | China-Guangdong | 2020 | DENV1 |
| MN444624 | Laos | 2020 | DENV1 |
| MW314720 | Cambodia | 2019 | DENV1 |
| MN921631 | China-Guangdong | 2020 | DENV1 |
| MN921630 | China-Guangdong | 2020 | DENV1 |
| MN923089 | China-Guangdong | 2020 | DENV1 |
| MN921392 | China-Guangdong | 2020 | DENV1 |
| MW014037 | China-Guangdong | 2020 | DENV1 |
| MN921447 | China-Guangdong | 2020 | DENV1 |
| MN894255 | China-Jiangxi | 2020 | DENV1 |
| MT705143 | China-Yunnan | 2020 | DENV1 |
| MW014038 | China-Guangdong | 2020 | DENV1 |
| MN923094 | China-Guangdong | 2020 | DENV1 |
| MN921299 | China-Guangdong | 2020 | DENV1 |
| MN921328 | China-Guangdong | 2020 | DENV1 |
| MN921391 | China-Guangdong | 2020 | DENV1 |
| MN921453 | China-Guangdong | 2020 | DENV1 |
| MW014043 | China-Guangdong | 2020 | DENV1 |
| MN921425 | China-Guangdong | 2020 | DENV1 |
| MN921332 | China-Guangdong | 2020 | DENV1 |
| MW228043 | Laos | 2020 | DENV1 |
| MN894226 | China-Jiangxi | 2020 | DENV1 |
| MN894240 | China-Jiangxi | 2020 | DENV1 |
| MT705047 | China-Yunnan | 2020 | DENV1 |
| MT705037 | China-Yunnan | 2020 | DENV1 |
| MN018307 | China-Guangdong | 2016 | DENV1 |
| MN912109 | Vietnam | 2018 | DENV1 |
| MN912224 | Vietnam | 2018 | DENV1 |
| KY971719 | Vietnam | 2016 | DENV1 |
| MN018334 | China-Guangdong | 2016 | DENV1 |
| MK566214 | China-Guangdong | 2018 | DENV1 |
| MN912202 | Vietnam | 2018 | DENV1 |
| MH010598 | China-Zhejiang | 2018 | DENV1 |
| MN912177 | Vietnam | 2018 | DENV1 |
| MN512235 | Guangxi | 2015 | DENV1 |
| LC428056 | Vietnam | 2018 | DENV1 |
| MH729968 | Vietnam | 2018 | DENV1 |
| LC428071 | Vietnam | 2018 | DENV1 |
| MT804538 | China-Guangdong | 2018 | DENV1 |
| LC428055 | Vietnam | 2018 | DENV1 |
| LC428060 | Vietnam | 2018 | DENV1 |
| MN018327 | China-Guangdong | 2018 | DENV1 |
| MN886839 | China-Guangdong | 2018 | DENV1 |
| LC428057 | Vietnam | 2018 | DENV1 |
| MN912152 | Vietnam | 2018 | DENV1 |
| LC428072 | Vietnam | 2018 | DENV1 |
| LC428078 | Vietnam | 2018 | DENV1 |
| LC428076 | Vietnam | 2018 | DENV1 |
| KY971718 | Vietnam | 2016 | DENV1 |
| KY971692 | Vietnam | 2010 | DENV1 |
| MN933652 | China-Guangdong | 2014 | DENV1 |
| AY732417 | Thailand | 1998 | DENV1 |
| KY586483 | Thailand | 1996 | DENV1 |
| KY586534 | Thailand | 1996 | DENV1 |
| KY586537 | Thailand | 1996 | DENV1 |
| KY586484 | Thailand | 1996 | DENV1 |
| KY586516 | Thailand | 1999 | DENV1 |
| KX056463 | China-Yunnan | 2016 | DENV1 |
| KX056464 | China-Yunnan | 2016 | DENV1 |
| KY672931 | China-Yunnan | 2016 | DENV1 |
| KY672932 | China-Yunnan | 2016 | DENV1 |
| KX357953 | Myanmar | 2016 | DENV1 |
| KX357958 | Myanmar | 2016 | DENV1 |
| KX056468 | China-Yunnan | 2016 | DENV1 |
| KJ470736 | China-Yunnan | 2014 | DENV1 |
| KR051925 | Myanmar | 2014 | DENV1 |
| KX357933 | Myanmar | 2016 | DENV1 |
| MH680501 | Singapore | 2016 | DENV1 |
| KJ806855 | Malaysia | 2014 | DENV1 |
| KJ470728 | China-Yunnan | 2014 | DENV1 |
| KJ939373 | China-Yunnan | 2014 | DENV1 |
| KJ470741 | Myanmar | 2014 | DENV1 |
| KJ939393 | China-Yunnan | 2014 | DENV1 |
| KT825001 | Myanmar | 2014 | DENV1 |
| KY038882 | China-Yunnan | 2014 | DENV1 |
| KY038883 | China-Yunnan | 2014 | DENV1 |
| KR051924 | Myanmar | 2014 | DENV1 |
| KJ470716 | China-Yunnan | 2014 | DENV1 |
| KJ470739 | Myanmar | 2014 | DENV1 |
| MN886914 | China-Guangdong | 2014 | DENV1 |
| KX056465 | China-Yunnan | 2016 | DENV1 |
| KJ939375 | China-Yunnan | 2014 | DENV1 |
| KJ470732 | China-Yunnan | 2014 | DENV1 |
| MG894723 | China-Taiwan | 2013 | DENV1 |
| MF033210 | Singapore | 2014 | DENV1 |
| KJ470737 | China-Yunnan | 2014 | DENV1 |
| KX056471 | China-Yunnan | 2016 | DENV1 |
| KJ470720 | China-Yunnan | 2014 | DENV1 |
| KJ939386 | China-Yunnan | 2014 | DENV1 |
| KY038884 | China-Yunnan | 2014 | DENV1 |
| KJ939385 | China-Yunnan | 2014 | DENV1 |
| KJ939390 | China-Yunnan | 2014 | DENV1 |
| MG894776 | China-Taiwan | 2014 | DENV1 |
| KJ470738 | Myanmar | 2014 | DENV1 |
| KJ470715 | China-Yunnan | 2014 | DENV1 |
| KJ939391 | China-Yunnan | 2014 | DENV1 |
| KJ939389 | China-Yunnan | 2014 | DENV1 |
| KR051926 | Myanmar | 2014 | DENV1 |
| KJ939368 | China-Yunnan | 2014 | DENV1 |
| KJ470740 | Myanmar | 2014 | DENV1 |
| KJ939392 | China-Yunnan | 2014 | DENV1 |
| KF559257 | Myanmar | 2010 | DENV1 |
| KJ470718 | China-Yunnan | 2014 | DENV1 |
| MG894871 | China-Taiwan | 2016 | DENV1 |
| KY586431 | Thailand | 2010 | DENV1 |
| KX357970 | Myanmar | 2016 | DENV1 |
| MF033202 | Singapore | 2013 | DENV1 |
| JF967810 | Myanmar | 2009 | DENV1 |
| KY038885 | China-Yunnan | 2013 | DENV1 |
| MG894763 | China-Taiwan | 2014 | DENV1 |
| KX056460 | China-Yunnan | 2014 | DENV1 |
| KJ806844 | Malaysia | 2014 | DENV1 |
| MG894773 | China-Taiwan | 2014 | DENV1 |
| MG894891 | China-Taiwan | 2016 | DENV1 |
| KF559256 | Myanmar | 2008 | DENV1 |
| JQ993132 | Thailand | 2008 | DENV1 |
| KY586428 | Thailand | 2007 | DENV1 |
| MF598849 | China-Yunnan | 2017 | DENV1 |
| MF598853 | China-Yunnan | 2017 | DENV1 |
| MF598850 | China-Yunnan | 2017 | DENV1 |
| MF598860 | China-Yunnan | 2017 | DENV1 |
| MF598858 | China-Yunnan | 2017 | DENV1 |
| MF598857 | China-Yunnan | 2017 | DENV1 |
| MF598861 | China-Yunnan | 2017 | DENV1 |
| MG933832 | China-Yunnan | 2018 | DENV1 |
| MG933837 | China-Yunnan | 2018 | DENV1 |
| MH729967 | Myanmar | 2018 | DENV1 |
| MG933828 | China-Yunnan | 2018 | DENV1 |
| MK529755 | China-Yunnan | 2019 | DENV1 |
| MN955630 | Thailand | 2018 | DENV1 |
| MN955652 | Thailand | 2019 | DENV1 |
| MN955631 | Thailand | 2018 | DENV1 |
| MK529743 | China-Yunnan | 2019 | DENV1 |
| LC410183 | Thailand | 2017 | DENV1 |
| MG679801 | Myanmar | 2018 | DENV1 |
| MT705151 | China-Yunnan | 2020 | DENV1 |
| MN955637 | Thailand | 2019 | DENV1 |
| KR051928 | Myanmar | 2014 | DENV1 |
| MG894740 | China-Taiwan | 2013 | DENV1 |
| MG894818 | China-Taiwan | 2015 | DENV1 |
| KT175104 | Myanmar | 2015 | DENV1 |
| KY672936 | China-Yunnan | 2016 | DENV1 |
| KY672935 | China-Yunnan | 2016 | DENV1 |
| KJ806941 | Singapore | 2014 | DENV1 |
| MG933835 | China-Yunnan | 2018 | DENV1 |
| KX357938 | Myanmar | 2016 | DENV1 |
| KX357967 | Myanmar | 2016 | DENV1 |
| MG933811 | China-Yunnan | 2018 | DENV1 |
| MN933716 | China-Guangdong | 2018 | DENV1 |
| KX357959 | Myanmar | 2016 | DENV1 |
| KX357917 | Myanmar | 2016 | DENV1 |
| KX357930 | Myanmar | 2016 | DENV1 |
| KX357954 | Myanmar | 2016 | DENV1 |
| KX357963 | Myanmar | 2016 | DENV1 |
| MW014049 | Thailand | 2019 | DENV1 |
| MN955653 | Thailand | 2019 | DENV1 |
| MN955656 | Thailand | 2019 | DENV1 |
| MN955658 | Thailand | 2019 | DENV1 |
| MN955643 | Thailand | 2019 | DENV1 |
| MN955649 | Thailand | 2019 | DENV1 |
| MN955657 | Thailand | 2019 | DENV1 |
| MG679800 | Myanmar | 2018 | DENV1 |
| MN923081 | China-Guangdong | 2020 | DENV1 |
| KX056466 | China-Yunnan | 2016 | DENV1 |
| KX056470 | China-Yunnan | 2016 | DENV1 |
| KR051921 | Myanmar | 2014 | DENV1 |
| KX357946 | Myanmar | 2016 | DENV1 |
| KY495792 | Thailand | 2016 | DENV1 |
| JF967879 | Thailand | 2010 | DENV1 |
| JQ993188 | Thailand | 2007 | DENV1 |
| JN415527 | Thailand | 2009 | DENV1 |
| KY586430 | Thailand | 2007 | DENV1 |
| EF113153 | China-Guangdong | 2007 | DENV1 |
| EF113152 | China-Guangdong | 2007 | DENV1 |
| EF508205 | China-Guangdong | 2007 | DENV1 |
| KX225491 | China-Guangdong | 2016 | DENV1 |
| JQ277850 | China-Guangdong | 2007 | DENV1 |
| JQ277874 | China-Guangdong | 2007 | DENV1 |
| FJ196856 | China-Guangdong | 2007 | DENV1 |
| JQ277849 | China-Guangdong | 2007 | DENV1 |
| FJ196855 | China-Guangdong | 2007 | DENV1 |
| FJ196858 | China-Guangdong | 2007 | DENV1 |
| KY586429 | Thailand | 2007 | DENV1 |
| MG933845 | China-Yunnan | 2018 | DENV1 |
| MG933825 | China-Yunnan | 2018 | DENV1 |
| MG933842 | China-Yunnan | 2018 | DENV1 |
| MG933843 | China-Yunnan | 2018 | DENV1 |
| MG933814 | China-Yunnan | 2018 | DENV1 |
| MG933821 | China-Yunnan | 2018 | DENV1 |
| MG933818 | China-Yunnan | 2018 | DENV1 |
| MG933824 | China-Yunnan | 2018 | DENV1 |
| MG933823 | China-Yunnan | 2018 | DENV1 |
| MG933826 | China-Yunnan | 2018 | DENV1 |
| MG933815 | China-Yunnan | 2018 | DENV1 |
| KX357915 | Myanmar | 2016 | DENV1 |
| KX357955 | Myanmar | 2016 | DENV1 |
| KX357951 | Myanmar | 2016 | DENV1 |
| KX357944 | Myanmar | 2016 | DENV1 |
| KR051912 | Myanmar | 2014 | DENV1 |
| KX056455 | China-Yunnan | 2015 | DENV1 |
| KX056453 | China-Yunnan | 2015 | DENV1 |
| KY672941 | China-Yunnan | 2015 | DENV1 |
| KY672942 | China-Yunnan | 2015 | DENV1 |
| KY038888 | China-Yunnan | 2015 | DENV1 |
| KJ470742 | Myanmar | 2014 | DENV1 |
| KJ939371 | China-Yunnan | 2014 | DENV1 |
| KJ939374 | China-Yunnan | 2014 | DENV1 |
| KJ939372 | China-Yunnan | 2014 | DENV1 |
| KX357965 | Myanmar | 2016 | DENV1 |
| KX357966 | Myanmar | 2016 | DENV1 |
| MG894961 | China-Taiwan | 2017 | DENV1 |
| MG933830 | China-Yunnan | 2018 | DENV1 |
| MG894898 | China-Taiwan | 2016 | DENV1 |
| KX357923 | Myanmar | 2016 | DENV1 |
| KR051913 | Myanmar | 2014 | DENV1 |
| KX056473 | China-Yunnan | 2016 | DENV1 |
| KX357945 | Myanmar | 2016 | DENV1 |
| MG894777 | China-Taiwan | 2014 | DENV1 |
| KX357916 | Myanmar | 2016 | DENV1 |
| KY038890 | China-Yunnan | 2016 | DENV1 |
| MN960676 | China-Guangdong | 2020 | DENV1 |
| KX357925 | Myanmar | 2016 | DENV1 |
| KX056472 | China-Yunnan | 2016 | DENV1 |
| KX357968 | Myanmar | 2016 | DENV1 |
| KX357932 | Myanmar | 2016 | DENV1 |
| MF405201 | China-Yunnan | 2016 | DENV1 |
| KX357918 | Myanmar | 2016 | DENV1 |
| KX056469 | China-Yunnan | 2016 | DENV1 |
| MF598851 | China-Yunnan | 2017 | DENV1 |
| MF598854 | China-Yunnan | 2017 | DENV1 |
| MF598852 | China-Yunnan | 2017 | DENV1 |
| MF598856 | China-Yunnan | 2017 | DENV1 |
| MG933829 | China-Yunnan | 2018 | DENV1 |
| MG933809 | China-Yunnan | 2018 | DENV1 |
| MG933820 | China-Yunnan | 2018 | DENV1 |
| MF598848 | China-Yunnan | 2017 | DENV1 |
| KX357948 | Myanmar | 2016 | DENV1 |
| KX357961 | Myanmar | 2016 | DENV1 |
| KX357924 | Myanmar | 2016 | DENV1 |
| MG933813 | China-Yunnan | 2018 | DENV1 |
| KX357941 | Myanmar | 2016 | DENV1 |
| MG933838 | China-Yunnan | 2018 | DENV1 |
| KX357928 | Myanmar | 2016 | DENV1 |
| KX357950 | Myanmar | 2016 | DENV1 |
| KT825058 | Thailand | 2014 | DENV1 |
| MG894894 | China-Taiwan | 2016 | DENV1 |
| KR051911 | Myanmar | 2014 | DENV1 |
| KU291653 | China-Guangdong | 2014 | DENV1 |
| KX357926 | Myanmar | 2016 | DENV1 |
| KU291654 | China-Guangdong | 2014 | DENV1 |
| KR051915 | Myanmar | 2014 | DENV1 |
| KJ806946 | Singapore | 2014 | DENV1 |
| KJ545443 | China-Guangdong | 2014 | DENV1 |
| KR051917 | Myanmar | 2014 | DENV1 |
| KJ545444 | China-Guangdong | 2014 | DENV1 |
| KT825014 | Thailand | 2014 | DENV1 |
| KX357956 | Myanmar | 2016 | DENV1 |
| MG894779 | China-Taiwan | 2014 | DENV1 |
| MG894771 | China-Taiwan | 2014 | DENV1 |
| MG894767 | China-Taiwan | 2014 | DENV1 |
| KJ545482 | Thailand | 2014 | DENV1 |
| KU291679 | China-Guangdong | 2014 | DENV1 |
| MG894766 | China-Taiwan | 2014 | DENV1 |
| KT824995 | Thailand | 2013 | DENV1 |
| MG894743 | China-Taiwan | 2013 | DENV1 |
| MF033201 | Singapore | 2013 | DENV1 |
| KX357911 | Myanmar | 2016 | DENV1 |
| KU509250 | Thailand | 2013 | DENV1 |
| KT824994 | Thailand | 2013 | DENV1 |
| MG564072 | Thailand | 2012 | DENV1 |
| MG564076 | Thailand | 2012 | DENV1 |
| KJ470722 | China-Yunnan | 2014 | DENV1 |
| KJ470729 | China-Yunnan | 2014 | DENV1 |
| KJ806843 | Malaysia | 2014 | DENV1 |
| MG894802 | China-Taiwan | 2014 | DENV1 |
| MF033221 | Singapore | 2015 | DENV1 |
| EF508204 | China-Guangdong | 2007 | DENV1 |
| KY586439 | Thailand | 2007 | DENV1 |
| KY586438 | Thailand | 2007 | DENV1 |
| KY978439 | India | 2016 | DENV1 |
| MK603943 | India | 2018 | DENV1 |
| KY978434 | India | 2013 | DENV1 |
| MH729961 | China-Fujian | 2016 | DENV1 |
| MH729960 | China-Fujian | 2016 | DENV1 |
| MN018303 | China-Guangdong | 2015 | DENV1 |
| MN018320 | China-Guangdong | 2015 | DENV1 |
| MT006147 | Sri Lanka | 2018 | DENV1 |
| JF960220 | Singapore | 2011 | DENV1 |
| KY586434 | Thailand | 2007 | DENV1 |
| KY586437 | Thailand | 2007 | DENV1 |
| KY586449 | Thailand | 2007 | DENV1 |
| KY586447 | Thailand | 2005 | DENV1 |
| KY586452 | Thailand | 2003 | DENV1 |
| KY586442 | Thailand | 2004 | DENV1 |
| EF508200 | China-Guangdong | 2002 | DENV1 |
| JQ993171 | Thailand | 2007 | DENV1 |
| KY586444 | Thailand | 2008 | DENV1 |
| KY586443 | Thailand | 2005 | DENV1 |
| KY586446 | Thailand | 2007 | DENV1 |
| KY586424 | Thailand | 2007 | DENV1 |
| KY586423 | Thailand | 2007 | DENV1 |
| KY586425 | Thailand | 2008 | DENV1 |
| EU448394 | Thailand | 2008 | DENV1 |
| AB608787 | China-Taiwan | 2009 | DENV1 |
| JQ403517 | China-Taiwan | 2009 | DENV1 |
| KY586440 | Thailand | 2006 | DENV1 |
| KY586479 | Thailand | 2003 | DENV1 |
| KY586535 | Thailand | 1996 | DENV1 |
| FJ410213 | Vietnam | 2009 | DENV1 |
| FJ882565 | Vietnam | 2004 | DENV1 |
| GQ199831 | Vietnam | 2004 | DENV1 |
| AB608789 | China-Taiwan | 1995 | DENV1 |
| AY732475 | Thailand | 1995 | DENV1 |
| AY732480 | Thailand | 1995 | DENV1 |
| AY713474 | Myanmar | 2002 | DENV1 |
| AY726552 | Myanmar | 2003 | DENV1 |
| AY713475 | Myanmar | 2002 | DENV1 |
| AY726550 | Myanmar | 2002 | DENV1 |
| AY726555 | Myanmar | 1999 | DENV1 |
| AY732478 | Thailand | 1992 | DENV1 |
| AY732477 | Thailand | 1992 | DENV1 |
| AB608788 | China-Taiwan | 1995 | DENV1 |
| AY732483 | Thailand | 1982 | DENV1 |
| MN018318 | China-Guangdong | 2017 | DENV1 |
| MN923080 | China-Guangdong | 2020 | DENV1 |
| MN018297 | China-Guangdong | 2017 | DENV1 |
| MN018290 | China-Guangdong | 2017 | DENV1 |
| MN923105 | China-Guangdong | 2020 | DENV1 |
| KJ933413 | China-Jiangsu | 2012 | DENV1 |
| MK566219 | China-Guangdong | 2018 | DENV1 |
| KC762628 | Indonesia | 2008 | DENV1 |
| KC762653 | Indonesia | 2008 | DENV1 |
| AY858983 | Indonesia | 2005 | DENV1 |
| JN205310 | China-Guangdong | 2003 | DENV1 |
| KC762630 | Indonesia | 2008 | DENV1 |
| AB189121 | Indonesia | 1999 | DENV1 |
| MK629461 | Indonesia | 2014 | DENV1 |
| KC762651 | Indonesia | 2008 | DENV1 |
| AB189120 | Indonesia | 1999 | DENV1 |
| KY021898 | India | 2016 | DENV1 |
| MH594882 | India | 2018 | DENV1 |
| KT175087 | Malaysia | 2015 | DENV1 |
| KT175110 | India | 2015 | DENV1 |
| MF427699 | India | 2017 | DENV1 |
| MF427700 | India | 2017 | DENV1 |
| KJ415095 | China-Zhejiang | 2014 | DENV1 |
| MK858141 | India | 2018 | DENV1 |
| MN933653 | China-Guangdong | 2014 | DENV1 |
| MH594884 | India | 2018 | DENV1 |
| KX056461 | China-Yunnan | 2014 | DENV1 |
| KY672944 | China-Yunnan | 2014 | DENV1 |
| KY038896 | China-Yunnan | 2014 | DENV1 |
| MF033204 | Singapore | 2013 | DENV1 |
| MK603945 | India | 2017 | DENV1 |
| MK603946 | India | 2017 | DENV1 |
| MK858139 | India | 2017 | DENV1 |
| MK858120 | India | 2017 | DENV1 |
| MH010600 | China-Zhejiang | 2018 | DENV1 |
| MK858119 | India | 2017 | DENV1 |
| MK858136 | India | 2017 | DENV1 |
| MG894782 | China-Taiwan | 2014 | DENV1 |
| MH822958 | India | 2013 | DENV1 |
| KY581731 | India | 2014 | DENV1 |
| KM216672 | India | 2011 | DENV1 |
| KY581730 | India | 2014 | DENV1 |
| JF967932 | India | 2011 | DENV1 |
| KU166894 | India | 2014 | DENV1 |
| KU166890 | India | 2014 | DENV1 |
| MG894729 | China-Taiwan | 2013 | DENV1 |
| MH680452 | Singapore | 2015 | DENV1 |
| KU291647 | China-Guangdong | 2014 | DENV1 |
| KJ415094 | China-Zhejiang | 2014 | DENV1 |
| KJ470730 | China-Yunnan | 2014 | DENV1 |
| MG894694 | China-Taiwan | 2012 | DENV1 |
| KT824969 | India | 2011 | DENV1 |
| JN415486 | India | 2011 | DENV1 |
| KU509255 | India | 2012 | DENV1 |
| MN913515 | China-Guangdong | 2019 | DENV1 |
| MT006166 | Sri Lanka | 2019 | DENV1 |
| MT006183 | Sri Lanka | 2018 | DENV1 |
| MT006182 | Sri Lanka | 2018 | DENV1 |
| MH594881 | India | 2018 | DENV1 |
| MH594888 | India | 2018 | DENV1 |
| MT006154 | Sri Lanka | 2019 | DENV1 |
| JX088742 | China-Guangdong | 2012 | DENV1 |
| KM216667 | India | 2011 | DENV1 |
| MH822959 | India | 2013 | DENV1 |
| MK858127 | India | 2017 | DENV1 |
| MK588396 | India | 2017 | DENV1 |
| MK858132 | India | 2017 | DENV1 |
| MK858128 | India | 2016 | DENV1 |
| MT126435 | India | 2015 | DENV1 |
| MK603954 | India | 2018 | DENV1 |
| MK603955 | India | 2018 | DENV1 |
| MN923086 | China-Guangdong | 2020 | DENV1 |
| MW295817 | China-Guangdong | 2021 | DENV1 |
| MH594893 | India | 2018 | DENV1 |
| KT825068 | India | 2016 | DENV1 |
| MH680497 | Singapore | 2016 | DENV1 |
| MH891767 | India | 2017 | DENV1 |
| MF033256 | Singapore | 2017 | DENV1 |
| MF033261 | Singapore | 2017 | DENV1 |
| MH454524 | India | 2017 | DENV1 |
| MK651231 | Bhutan | 2018 | DENV1 |
| MH891771 | India | 2018 | DENV1 |
| MH454523 | India | 2017 | DENV1 |
| MF033226 | Singapore | 2015 | DENV1 |
| MH680486 | Singapore | 2016 | DENV1 |
| MF033212 | Singapore | 2014 | DENV1 |
| MK588397 | India | 2017 | DENV1 |
| MG721060 | India | 2017 | DENV1 |
| KX721476 | India | 2016 | DENV1 |
| KX721481 | India | 2016 | DENV1 |
| KX721479 | India | 2016 | DENV1 |
| KX721482 | India | 2016 | DENV1 |
| KU166896 | India | 2014 | DENV1 |
| KY581734 | India | 2014 | DENV1 |
| KU166895 | India | 2014 | DENV1 |
| KY581735 | India | 2015 | DENV1 |
| JQ692085 | India | 2011 | DENV1 |
| KF289072 | India | 2012 | DENV1 |
| MT126437 | India | 2017 | DENV1 |
| MT126440 | India | 2018 | DENV1 |
| MT126436 | India | 2016 | DENV1 |
| MK603942 | India | 2018 | DENV1 |
| MK603950 | India | 2019 | DENV1 |
| MH594887 | India | 2018 | DENV1 |
| MT126438 | India | 2017 | DENV1 |
| MH594885 | India | 2018 | DENV1 |
| MH594886 | India | 2018 | DENV1 |
| MN923082 | China-Guangdong | 2020 | DENV1 |
| KY978440 | India | 2016 | DENV1 |
| MF033253 | Singapore | 2016 | DENV1 |
| KY021899 | India | 2016 | DENV1 |
| MN923085 | China-Guangdong | 2020 | DENV1 |
| MN923101 | China-Guangdong | 2019 | DENV1 |
| MK603953 | India | 2018 | DENV1 |
| MK858140 | India | 2017 | DENV1 |
| MF033237 | Singapore | 2016 | DENV1 |
| MN886850 | China-Guangdong | 2015 | DENV1 |
| MT804483 | China-Guangdong | 2015 | DENV1 |
| KP686070 | China-Henan | 2015 | DENV1 |
| KR071622 | China-Hubei | 2015 | DENV1 |
| KR024705 | China-Hubei | 2015 | DENV1 |
| MN886858 | China-Guangdong | 2015 | DENV1 |
| MN886866 | China-Guangdong | 2015 | DENV1 |
| KM403632 | Singapore | 2014 | DENV1 |
| MW014047 | China-Guangdong | 2019 | DENV1 |
| LC436668 | Bangladesh | 2018 | DENV1 |
| LC436610 | Bangladesh | 2018 | DENV1 |
| KM403627 | Singapore | 2014 | DENV1 |
| KR024708 | China-Zhejiang | 2015 | DENV1 |
| KM403592 | Singapore | 2014 | DENV1 |
| KM403596 | Singapore | 2014 | DENV1 |
| MH679963 | Singapore | 2013 | DENV1 |
| KM403578 | Singapore | 2014 | DENV1 |
| KM403576 | Singapore | 2014 | DENV1 |
| KM403589 | Singapore | 2014 | DENV1 |
| KM403590 | Singapore | 2014 | DENV1 |
| LC436612 | Bangladesh | 2018 | DENV1 |
| MN018286 | China-Guangdong | 2017 | DENV1 |
| KM403605 | Singapore | 2014 | DENV1 |
| MH680300 | Singapore | 2014 | DENV1 |
| JQ917404 | India | 2010 | DENV1 |
| JQ922548 | India | 2006 | DENV1 |
| JQ922545 | India | 1983 | DENV1 |
| MK858137 | India | 2017 | DENV1 |
| AY732474 | Thailand | 1981 | DENV1 |
| AY732476 | Thailand | 1981 | DENV1 |
| KF289073 | India | 1957 | DENV1 |
| AY722802 | Myanmar | 1997 | DENV1 |
| AY722803 | Myanmar | 1999 | DENV1 |
| AY722801 | Myanmar | 1977 | DENV1 |
| AY713473 | Myanmar | 1972 | DENV1 |
| JQ922546 | India | 1972 | DENV1 |
| JQ922544 | India | 1964 | DENV1 |
| EF457905 | Malaysia | 1973 | DENV1 |
| MK949428 | China-Hunan | 2019 | DENV2 |
| MH827554 | China-Guangdong | 2018 | DENV2 |
| MK564481 | China-Guangdong | 2018 | DENV2 |
| MT252659 | Singapore | 2018 | DENV2 |
| MN923152 | China-Guangdong | 2019 | DENV2 |
| MN913512 | China-Guangdong | 2020 | DENV2 |
| MK789429 | China-Guangdong | 2018 | DENV2 |
| MH729986 | China-Fujian | 2018 | DENV2 |
| MH827547 | China-Guangdong | 2018 | DENV2 |
| MT252653 | Singapore | 2018 | DENV2 |
| MK587782 | China-Guangdong | 2018 | DENV2 |
| MT252657 | Singapore | 2018 | DENV2 |
| MT252658 | Singapore | 2018 | DENV2 |
| MN955682 | Thailand | 2019 | DENV2 |
| MK587779 | China-Guangdong | 2017 | DENV2 |
| MN018344 | China-Guangdong | 2017 | DENV2 |
| MN018343 | China-Guangdong | 2017 | DENV2 |
| MH110717 | China-Zhejiang | 2018 | DENV2 |
| MT252652 | Singapore | 2018 | DENV2 |
| MW295816 | China-Guangdong | 2021 | DENV2 |
| MW295818 | China-Guangdong | 2021 | DENV2 |
| MN913513 | China-Guangdong | 2020 | DENV2 |
| MN955671 | Thailand | 2018 | DENV2 |
| MN955676 | Thailand | 2018 | DENV2 |
| MN018354 | China-Guangdong | 2017 | DENV2 |
| MT006171 | Sri Lanka | 2018 | DENV2 |
| MT006181 | Sri Lanka | 2018 | DENV2 |
| MT252651 | Singapore | 2018 | DENV2 |
| MT006145 | Sri Lanka | 2018 | DENV2 |
| MT006184 | Sri Lanka | 2018 | DENV2 |
| MH827548 | China-Guangdong | 2018 | DENV2 |
| MN018341 | China-Guangdong | 2017 | DENV2 |
| MT006153 | Sri Lanka | 2018 | DENV2 |
| MT006173 | Sri Lanka | 2018 | DENV2 |
| MT006162 | Sri Lanka | 2018 | DENV2 |
| MT006163 | Sri Lanka | 2019 | DENV2 |
| MT006142 | Sri Lanka | 2018 | DENV2 |
| MT252655 | Singapore | 2018 | DENV2 |
| MT006148 | Sri Lanka | 2018 | DENV2 |
| MT006175 | Sri Lanka | 2018 | DENV2 |
| MK587780 | China-Guangdong | 2018 | DENV2 |
| MT006160 | Sri Lanka | 2018 | DENV2 |
| MT006186 | Sri Lanka | 2018 | DENV2 |
| MT006159 | Sri Lanka | 2018 | DENV2 |
| MK564483 | China-Guangdong | 2018 | DENV2 |
| MK513444 | Singapore | 2015 | DENV2 |
| MT252643 | Singapore | 2016 | DENV2 |
| MH827551 | China-Guangdong | 2018 | DENV2 |
| MN018342 | China-Guangdong | 2017 | DENV2 |
| MT252654 | Singapore | 2018 | DENV2 |
| MH729980 | Singapore | 2016 | DENV2 |
| MH827537 | China-Guangdong | 2018 | DENV2 |
| MH729972 | Philippines | 2016 | DENV2 |
| MT705611 | China-Yunnan | 2020 | DENV2 |
| MT705614 | China-Yunnan | 2020 | DENV2 |
| MW018170 | China-Guangdong | 2020 | DENV2 |
| MW018166 | China-Guangdong | 2020 | DENV2 |
| MW018168 | China-Guangdong | 2020 | DENV2 |
| MN913511 | China-Guangdong | 2020 | DENV2 |
| MN923139 | China-Guangdong | 2019 | DENV2 |
| MN913507 | China-Guangdong | 2019 | DENV2 |
| MN955685 | Thailand | 2019 | DENV2 |
| MK780865 | Thailand | 2016 | DENV2 |
| MK780860 | Thailand | 2016 | DENV2 |
| MK780863 | Thailand | 2016 | DENV2 |
| MN923169 | China-Guangdong | 2019 | DENV2 |
| MN923155 | China-Guangdong | 2019 | DENV2 |
| MK780868 | Thailand | 2017 | DENV2 |
| MN923170 | China-Guangdong | 2019 | DENV2 |
| MN955684 | Thailand | 2019 | DENV2 |
| MW018171 | Thailand | 2020 | DENV2 |
| MN955679 | Thailand | 2019 | DENV2 |
| MK780867 | Thailand | 2016 | DENV2 |
| MH827528 | China-Guangdong | 2018 | DENV2 |
| MN018337 | China-Guangdong | 2016 | DENV2 |
| MH827533 | China-Guangdong | 2018 | DENV2 |
| MN018338 | China-Guangdong | 2016 | DENV2 |
| MH827536 | China-Guangdong | 2018 | DENV2 |
| MN018346 | China-Guangdong | 2016 | DENV2 |
| MH729981 | China-Fujian | 2017 | DENV2 |
| MH729982 | China-Fujian | 2017 | DENV2 |
| MH729983 | China-Fujian | 2017 | DENV2 |
| MH729975 | China-Fujian | 2016 | DENV2 |
| MK587781 | China-Guangdong | 2018 | DENV2 |
| MK587783 | China-Guangdong | 2018 | DENV2 |
| MK411558 | Thailand | 2016 | DENV2 |
| MK411559 | Indonesia | 2016 | DENV2 |
| MH823208 | Indonesia | 2015 | DENV2 |
| MT252650 | Singapore | 2017 | DENV2 |
| KT012518 | Indonesia | 2015 | DENV2 |
| MH827530 | China-Guangdong | 2018 | DENV2 |
| EU448430 | Indonesia | 2008 | DENV2 |
| EU069578 | Singapore | 2005 | DENV2 |
| MN018353 | China-Guangdong | 2017 | DENV2 |
| MT252645 | Singapore | 2017 | DENV2 |
| MK629473 | Indonesia | 2017 | DENV2 |
| MT252644 | Singapore | 2016 | DENV2 |
| MH827525 | China-Guangdong | 2018 | DENV2 |
| MT252648 | Singapore | 2017 | DENV2 |
| MG596006 | China-Guangdong | 2017 | DENV2 |
| MK578532 | China-Guangdong | 2016 | DENV2 |
| KP723479 | China-Guangdong | 2011 | DENV2 |
| KY882458 | China-Guangdong | 2017 | DENV2 |
| JX470186 | China-Guangdong | 2011 | DENV2 |
| MT705606 | China-Yunnan | 2020 | DENV2 |
| MG895149 | China-Taiwan | 2017 | DENV2 |
| MT377729 | Indonesia | 2020 | DENV2 |
| MG895060 | China-Taiwan | 2015 | DENV2 |
| KJ806885 | Malaysia | 2014 | DENV2 |
| KF052657 | Indonesia | 2013 | DENV2 |
| KT175122 | Indonesia | 2014 | DENV2 |
| KC589011 | Indonesia | 2012 | DENV2 |
| KF052658 | Indonesia | 2013 | DENV2 |
| MG895153 | China-Taiwan | 2017 | DENV2 |
| KF052652 | Indonesia | 2013 | DENV2 |
| KF052663 | Indonesia | 2013 | DENV2 |
| LC064746 | Indonesia | 2016 | DENV2 |
| KT175113 | China-Taiwan | 2014 | DENV2 |
| JF968014 | Indonesia | 2011 | DENV2 |
| KT781547 | Indonesia | 2014 | DENV2 |
| KU529746 | Indonesia | 2016 | DENV2 |
| JF968002 | Indonesia | 2010 | DENV2 |
| MN923153 | China-Guangdong | 2019 | DENV2 |
| KJ806783 | Malaysia | 2014 | DENV2 |
| KY275202 | East Timor | 2011 | DENV2 |
| KY275215 | East Timor | 2011 | DENV2 |
| KC762680 | Indonesia | 2010 | DENV2 |
| MG895022 | China-Taiwan | 2013 | DENV2 |
| KC762672 | Indonesia | 2008 | DENV2 |
| GQ357790 | Singapore | 2008 | DENV2 |
| JF968035 | Malaysia | 2011 | DENV2 |
| KX380807 | Singapore | 2013 | DENV2 |
| MG895025 | China-Taiwan | 2013 | DENV2 |
| MG895005 | China-Taiwan | 2012 | DENV2 |
| MK564478 | China-Guangdong | 2016 | DENV2 |
| MT845880 | China-Guangdong | 2017 | DENV2 |
| KX270815 | China-Guangdong/Guangzhou | 2011 | DENV2 |
| JX093609 | Vietnam | 2012 | DENV2 |
| JX093594 | Vietnam | 2012 | DENV2 |
| GU908496 | Vietnam | 2010 | DENV2 |
| DQ518636 | Malaysia | 2005 | DENV2 |
| MK564482 | China-Guangdong | 2018 | DENV2 |
| MN955675 | Thailand | 2018 | DENV2 |
| MN955683 | Thailand | 2019 | DENV2 |
| MT252649 | Singapore | 2017 | DENV2 |
| MT252646 | Singapore | 2017 | DENV2 |
| MN018352 | China-Guangdong | 2016 | DENV2 |
| MH827527 | China-Guangdong | 2018 | DENV2 |
| MN018349 | China-Guangdong | 2015 | DENV2 |
| MK629474 | Indonesia | 2016 | DENV2 |
| MK564477 | China-Guangdong | 2016 | DENV2 |
| KM279556 | Singapore | 2013 | DENV2 |
| JN544368 | Singapore | 2011 | DENV2 |
| JN851116 | Singapore | 2008 | DENV2 |
| JN851113 | Singapore | 2007 | DENV2 |
| JN196569 | Singapore | 2010 | DENV2 |
| JN380855 | Singapore | 2011 | DENV2 |
| MH827526 | China-Guangdong | 2018 | DENV2 |
| MN018351 | China-Guangdong | 2015 | DENV2 |
| MH827544 | China-Guangdong | 2018 | DENV2 |
| MK629472 | Indonesia | 2017 | DENV2 |
| DQ518634 | Indonesia | 2005 | DENV2 |
| MN018350 | China-Guangdong | 2017 | DENV2 |
| MH827545 | China-Guangdong | 2018 | DENV2 |
| MN923133 | China-Guangdong | 2018 | DENV2 |
| MH729973 | Philippines | 2016 | DENV2 |
| MN944002 | China-Guangdong | 2020 | DENV2 |
| AB189124 | Indonesia | 1999 | DENV2 |
| AB189122 | Indonesia | 1999 | DENV2 |
| MT252656 | Singapore | 2018 | DENV2 |
| GQ398264 | Indonesia | 1977 | DENV2 |
| GQ398263 | Indonesia | 1976 | DENV2 |
| GQ398260 | Indonesia | 1977 | DENV2 |
| GQ398261 | Indonesia | 1977 | DENV2 |
| GQ398262 | Indonesia | 1977 | DENV2 |
| GQ398258 | Indonesia | 1976 | DENV2 |
| GQ398259 | Indonesia | 1977 | DENV2 |
| MF156233 | China-Yunnan | 2016 | DENV2 |
| MG895150 | China-Taiwan | 2017 | DENV2 |
| MF156243 | China-Yunnan | 2016 | DENV2 |
| MF940236 | China-Yunnan | 2016 | DENV2 |
| MH729977 | China-Fujian | 2016 | DENV2 |
| MH729978 | China-Fujian | 2016 | DENV2 |
| MH729979 | China-Fujian | 2016 | DENV2 |
| KX262953 | China-Yunnan | 2016 | DENV2 |
| KX262952 | China-Yunnan | 2016 | DENV2 |
| MF940240 | China-Yunnan | 2016 | DENV2 |
| MF940252 | China-Yunnan | 2016 | DENV2 |
| MG593025 | China-Guangdong | 2016 | DENV2 |
| KX082961 | China-Guangdong | 2016 | DENV2 |
| KX082959 | China-Guangdong | 2016 | DENV2 |
| MK587775 | China-Guangdong | 2016 | DENV2 |
| MK587776 | China-Guangdong | 2016 | DENV2 |
| KX082963 | China-Guangdong | 2016 | DENV2 |
| MH827535 | China-Guangdong | 2018 | DENV2 |
| MF156238 | China-Yunnan | 2016 | DENV2 |
| MF156240 | China-Yunnan | 2016 | DENV2 |
| MF940239 | China-Yunnan | 2016 | DENV2 |
| MF940251 | China-Yunnan | 2016 | DENV2 |
| KX262950 | China-Yunnan | 2016 | DENV2 |
| KY672953 | China-Yunnan | 2016 | DENV2 |
| MF940242 | China-Yunnan | 2016 | DENV2 |
| MF940238 | China-Yunnan | 2016 | DENV2 |
| MF940253 | China-Yunnan | 2016 | DENV2 |
| MF940241 | China-Yunnan | 2016 | DENV2 |
| MF940243 | China-Yunnan | 2016 | DENV2 |
| MF940250 | China-Yunnan | 2016 | DENV2 |
| MF940237 | China-Yunnan | 2016 | DENV2 |
| MF940246 | China-Yunnan | 2016 | DENV2 |
| MF940249 | China-Yunnan | 2016 | DENV2 |
| KX262946 | China-Yunnan | 2016 | DENV2 |
| KY672952 | China-Yunnan | 2016 | DENV2 |
| MF156237 | China-Yunnan | 2016 | DENV2 |
| MG596005 | China-Guangdong | 2017 | DENV2 |
| MK578531 | China-Guangdong | 2016 | DENV2 |
| MH110615 | China-Zhejiang | 2018 | DENV2 |
| KR071787 | China-Guangdong | 2015 | DENV2 |
| KT781567 | Vietnam | 2015 | DENV2 |
| KY971740 | Vietnam | 2016 | DENV2 |
| KY971736 | Vietnam | 2016 | DENV2 |
| KY971728 | Vietnam | 2016 | DENV2 |
| KY971738 | Vietnam | 2016 | DENV2 |
| KY971739 | Vietnam | 2016 | DENV2 |
| KY971725 | Vietnam | 2016 | DENV2 |
| MG895118 | China-Taiwan | 2016 | DENV2 |
| KY971724 | Vietnam | 2016 | DENV2 |
| KY971726 | Vietnam | 2016 | DENV2 |
| KY971727 | Vietnam | 2016 | DENV2 |
| KY971721 | Vietnam | 2016 | DENV2 |
| MG895121 | China-Taiwan | 2016 | DENV2 |
| KY971722 | Vietnam | 2016 | DENV2 |
| KY038906 | China-Yunnan | 2016 | DENV2 |
| KY038908 | China-Yunnan | 2016 | DENV2 |
| KY038907 | China-Yunnan | 2016 | DENV2 |
| KY038904 | China-Yunnan | 2016 | DENV2 |
| KY038902 | China-Yunnan | 2016 | DENV2 |
| KY038898 | China-Yunnan | 2016 | DENV2 |
| KY971735 | Vietnam | 2016 | DENV2 |
| MG895120 | China-Taiwan | 2016 | DENV2 |
| KY971730 | Vietnam | 2016 | DENV2 |
| KY971741 | Vietnam | 2016 | DENV2 |
| KY971732 | Vietnam | 2016 | DENV2 |
| MG895119 | China-Taiwan | 2016 | DENV2 |
| KY971723 | Vietnam | 2016 | DENV2 |
| MH827529 | China-Guangdong | 2018 | DENV2 |
| MN018339 | China-Guangdong | 2015 | DENV2 |
| KT781566 | Thailand | 2015 | DENV2 |
| KT175134 | Thailand | 2015 | DENV2 |
| KU291798 | China-Guangdong | 2014 | DENV2 |
| MH822942 | India | 2015 | DENV2 |
| MT182726 | India | 2018 | DENV2 |
| MT182727 | India | 2018 | DENV2 |
| MH010616 | China-Zhejiang | 2018 | DENV2 |
| MG721062 | India | 2017 | DENV2 |
| MK858101 | India | 2017 | DENV2 |
| MG721058 | India | 2017 | DENV2 |
| MK858111 | India | 2017 | DENV2 |
| MK858107 | India | 2017 | DENV2 |
| MG721055 | India | 2017 | DENV2 |
| MG721057 | India | 2017 | DENV2 |
| MK858108 | India | 2017 | DENV2 |
| MH891772 | India | 2018 | DENV2 |
| MH594927 | India | 2018 | DENV2 |
| MK858103 | India | 2017 | DENV2 |
| MK858110 | India | 2017 | DENV2 |
| KY937188 | China-Yunnan | 2016 | DENV2 |
| KF479233 | China-Hebei | 2013 | DENV2 |
| MG895044 | China-Taiwan | 2014 | DENV2 |
| MH822944 | India | 2013 | DENV2 |
| KJ806910 | Malaysia | 2014 | DENV2 |
| MG895021 | China-Taiwan | 2013 | DENV2 |
| KJ806792 | Malaysia | 2014 | DENV2 |
| KT806312 | India | 2012 | DENV2 |
| KT781565 | India | 2015 | DENV2 |
| MG894990 | China-Taiwan | 2012 | DENV2 |
| MK858115 | India | 2018 | DENV2 |
| MK858114 | India | 2018 | DENV2 |
| MH594925 | India | 2018 | DENV2 |
| MH594926 | India | 2018 | DENV2 |
| MT182729 | India | 2017 | DENV2 |
| KJ806779 | Malaysia | 2014 | DENV2 |
| KX380828 | Singapore | 2014 | DENV2 |
| KX380829 | Singapore | 2014 | DENV2 |
| MW018162 | China-Guangdong | 2019 | DENV2 |
| MW018163 | China-Guangdong | 2019 | DENV2 |
| MG895048 | China-Taiwan | 2014 | DENV2 |
| MK858097 | India | 2014 | DENV2 |
| MG895046 | China-Taiwan | 2014 | DENV2 |
| MH822945 | India | 2013 | DENV2 |
| KT781518 | India | 2010 | DENV2 |
| KX270809 | China-Guangdong | 2012 | DENV2 |
| MH822943 | India | 2015 | DENV2 |
| KY937186 | China-Yunnan | 2016 | DENV2 |
| KY937187 | China-Yunnan | 2016 | DENV2 |
| JN030339 | Singapore | 2011 | DENV2 |
| JN030342 | Singapore | 2011 | DENV2 |
| JN030341 | Singapore | 2011 | DENV2 |
| JN030333 | Singapore | 2011 | DENV2 |
| JN030334 | Singapore | 2011 | DENV2 |
| JN030340 | Singapore | 2011 | DENV2 |
| KJ545481 | Thailand | 2014 | DENV2 |
| KU291801 | China-Guangdong | 2014 | DENV2 |
| KJ545478 | India | 2014 | DENV2 |
| KU291799 | China-Guangdong | 2014 | DENV2 |
| KJ806815 | Malaysia | 2015 | DENV2 |
| MG895047 | China-Taiwan | 2014 | DENV2 |
| KT781572 | Thailand | 2016 | DENV2 |
| KX224267 | Singapore | 2015 | DENV2 |
| MH822939 | India | 2013 | DENV2 |
| MG895045 | China-Taiwan | 2014 | DENV2 |
| MH891768 | India | 2017 | DENV2 |
| MH822940 | India | 2015 | DENV2 |
| MH822941 | India | 2014 | DENV2 |
| MG895041 | China-Taiwan | 2014 | DENV2 |
| JN568258 | India | 2010 | DENV2 |
| KF577806 | India | 2011 | DENV2 |
| JQ955624 | India | 2012 | DENV2 |
| JF967992 | India | 2010 | DENV2 |
| DQ448237 | India | 2002 | DENV2 |
| DQ448235 | India | 2002 | DENV2 |
| KU509271 | India | 2007 | DENV2 |
| KY978441 | India | 2013 | DENV2 |
| KY978444 | India | 2013 | DENV2 |
| KY978442 | India | 2013 | DENV2 |
| KY978443 | India | 2013 | DENV2 |
| JQ922549 | India | 1997 | DENV2 |
| FJ538925 | India | 1993 | DENV2 |
| FJ538912 | India | 1996 | DENV2 |
| FJ538913 | India | 1998 | DENV2 |
| FJ538924 | India | 1991 | DENV2 |
| FJ538911 | India | 1994 | DENV2 |
| MK517751 | China-Guangdong | 2019 | DENV2 |
| MK578533 | China-Guangdong | 2019 | DENV2 |
| MN913508 | China-Guangdong | 2019 | DENV2 |
| MW018161 | China-Guangdong | 2019 | DENV2 |
| MK517752 | China-Guangdong | 2019 | DENV2 |
| MN913509 | China-Guangdong | 2019 | DENV2 |
| MN913510 | China-Guangdong | 2019 | DENV2 |
| MK564488 | China-Guangdong | 2018 | DENV2 |
| MN923166 | China-Guangdong | 2018 | DENV2 |
| MK651230 | Bhutan | 2017 | DENV2 |
| MK651227 | Bhutan | 2017 | DENV2 |
| MK651229 | Bhutan | 2017 | DENV2 |
| MK858096 | India | 2015 | DENV2 |
| LC436618 | Bangladesh | 2018 | DENV2 |
| LC436619 | Bangladesh | 2018 | DENV2 |
| MK858113 | India | 2018 | DENV2 |
| MK858099 | India | 2017 | DENV2 |
| MK858106 | India | 2017 | DENV2 |
| MK858100 | India | 2017 | DENV2 |
| MK858104 | India | 2017 | DENV2 |
| MK858102 | India | 2017 | DENV2 |
| MK858116 | India | 2018 | DENV2 |
| MT182728 | India | 2017 | DENV2 |
| MT182725 | India | 2018 | DENV2 |
| MT182730 | India | 2016 | DENV2 |
| MH891770 | India | 2018 | DENV2 |
| MK858109 | India | 2017 | DENV2 |
| MT182731 | India | 2015 | DENV2 |
| FJ538920 | India | 1975 | DENV2 |
| FJ538922 | India | 1984 | DENV2 |
| FJ538923 | India | 1992 | DENV2 |
| KX357979 | Myanmar | 2016 | DENV2 |
| MG895113 | China-Taiwan | 2016 | DENV2 |
| KX262938 | China-Yunnan | 2016 | DENV2 |
| KX262934 | China-Yunnan | 2016 | DENV2 |
| KX262929 | China-Yunnan | 2016 | DENV2 |
| KX262941 | China-Yunnan | 2016 | DENV2 |
| KT806324 | Myanmar | 2015 | DENV2 |
| KX357978 | Myanmar | 2016 | DENV2 |
| KT175136 | Myanmar | 2015 | DENV2 |
| KT175115 | China-Taiwan | 2015 | DENV2 |
| MN955665 | Thailand | 2019 | DENV2 |
| MN955667 | Thailand | 2019 | DENV2 |
| MG895086 | China-Taiwan | 2016 | DENV2 |
| KX262940 | China-Yunnan | 2016 | DENV2 |
| MN955664 | Thailand | 2019 | DENV2 |
| KY038912 | China-Yunnan | 2015 | DENV2 |
| KY038913 | China-Yunnan | 2015 | DENV2 |
| KY038914 | China-Yunnan | 2015 | DENV2 |
| KJ470761 | Myanmar | 2014 | DENV2 |
| KJ939404 | China-Yunnan | 2014 | DENV2 |
| MT705582 | China-Yunnan | 2020 | DENV2 |
| MT705583 | China-Yunnan | 2020 | DENV2 |
| MT705594 | China-Yunnan | 2020 | DENV2 |
| MT705604 | China-Yunnan | 2020 | DENV2 |
| MT705609 | China-Yunnan | 2020 | DENV2 |
| MT705610 | China-Yunnan | 2020 | DENV2 |
| MT705608 | China-Yunnan | 2020 | DENV2 |
| MT705605 | China-Yunnan | 2020 | DENV2 |
| MT705574 | China-Yunnan | 2020 | DENV2 |
| MT705595 | China-Yunnan | 2020 | DENV2 |
| MT705584 | China-Yunnan | 2020 | DENV2 |
| MH827532 | China-Guangdong | 2018 | DENV2 |
| MN018345 | China-Guangdong | 2016 | DENV2 |
| KX262932 | China-Yunnan | 2016 | DENV2 |
| MK780871 | Thailand | 2016 | DENV2 |
| MK780873 | Thailand | 2016 | DENV2 |
| MG895080 | China-Taiwan | 2015 | DENV2 |
| KX262927 | China-Yunnan | 2016 | DENV2 |
| KY672945 | China-Yunnan | 2016 | DENV2 |
| MG895156 | China-Taiwan | 2017 | DENV2 |
| MG596009 | China-Guangdong | 2017 | DENV2 |
| MN955668 | Thailand | 2019 | DENV2 |
| MG933846 | China-Yunnan | 2018 | DENV2 |
| KY672955 | China-Yunnan | 2016 | DENV2 |
| KX262935 | China-Yunnan | 2016 | DENV2 |
| MW018159 | China-Guangdong | 2019 | DENV2 |
| MN955662 | Thailand | 2018 | DENV2 |
| KX262939 | China-Yunnan | 2016 | DENV2 |
| MG564100 | Thailand | 2016 | DENV2 |
| MG564101 | Thailand | 2016 | DENV2 |
| KX357977 | Myanmar | 2016 | DENV2 |
| MG895115 | China-Taiwan | 2016 | DENV2 |
| KX262943 | China-Yunnan | 2016 | DENV2 |
| KX262928 | China-Yunnan | 2016 | DENV2 |
| KX357990 | Myanmar | 2016 | DENV2 |
| MK780878 | Thailand | 2016 | DENV2 |
| MK780870 | Thailand | 2016 | DENV2 |
| MK780876 | Thailand | 2016 | DENV2 |
| KX262942 | China-Yunnan | 2016 | DENV2 |
| KX357992 | Myanmar | 2016 | DENV2 |
| KX357999 | Myanmar | 2016 | DENV2 |
| KX262936 | China-Yunnan | 2016 | DENV2 |
| MG895038 | China-Taiwan | 2014 | DENV2 |
| KX357995 | Myanmar | 2016 | DENV2 |
| MK780872 | Thailand | 2016 | DENV2 |
| KY038915 | China-Yunnan | 2015 | DENV2 |
| KX357994 | Myanmar | 2016 | DENV2 |
| KY672946 | China-Yunnan | 2016 | DENV2 |
| LC410187 | Thailand | 2017 | DENV2 |
| LC410188 | Thailand | 2017 | DENV2 |
| MG895103 | China-Taiwan | 2016 | DENV2 |
| MK780879 | Thailand | 2017 | DENV2 |
| KX357982 | Myanmar | 2016 | DENV2 |
| MG885749 | India | 2018 | DENV2 |
| MK780874 | Thailand | 2017 | DENV2 |
| MK780877 | Thailand | 2016 | DENV2 |
| LC410184 | Thailand | 2017 | DENV2 |
| MG895032 | China-Taiwan | 2014 | DENV2 |
| KX357980 | Myanmar | 2016 | DENV2 |
| KJ470757 | Myanmar | 2014 | DENV2 |
| KY672947 | China-Yunnan | 2014 | DENV2 |
| KJ470760 | Myanmar | 2014 | DENV2 |
| KJ470744 | China-Yunnan | 2014 | DENV2 |
| KJ939401 | China-Yunnan | 2014 | DENV2 |
| KJ939394 | China-Yunnan | 2014 | DENV2 |
| KJ470758 | Myanmar | 2014 | DENV2 |
| KJ939406 | China-Yunnan | 2014 | DENV2 |
| KJ939376 | China-Yunnan | 2014 | DENV2 |
| KJ939384 | China-Yunnan | 2014 | DENV2 |
| KJ939382 | China-Yunnan | 2014 | DENV2 |
| KX262954 | China-Yunnan | 2014 | DENV2 |
| KJ939378 | China-Yunnan | 2014 | DENV2 |
| KX262957 | China-Yunnan | 2014 | DENV2 |
| KY672949 | China-Yunnan | 2014 | DENV2 |
| KY038917 | China-Yunnan | 2014 | DENV2 |
| KY038916 | China-Yunnan | 2014 | DENV2 |
| KJ470762 | Myanmar | 2014 | DENV2 |
| KJ939399 | China-Yunnan | 2014 | DENV2 |
| KT175138 | Myanmar | 2015 | DENV2 |
| MG895071 | China-Taiwan | 2015 | DENV2 |
| KX262956 | China-Yunnan | 2014 | DENV2 |
| KJ470745 | China-Yunnan | 2014 | DENV2 |
| KJ939398 | China-Yunnan | 2014 | DENV2 |
| KJ939397 | China-Yunnan | 2014 | DENV2 |
| KJ470743 | China-Yunnan | 2014 | DENV2 |
| KJ939396 | China-Yunnan | 2014 | DENV2 |
| KX262937 | China-Yunnan | 2016 | DENV2 |
| KY038911 | China-Yunnan | 2014 | DENV2 |
| KY038910 | China-Yunnan | 2014 | DENV2 |
| MG895020 | China-Taiwan | 2013 | DENV2 |
| MG895042 | China-Taiwan | 2014 | DENV2 |
| KR051903 | Myanmar | 2014 | DENV2 |
| KY586626 | Thailand | 2010 | DENV2 |
| MG564090 | Thailand | 2010 | DENV2 |
| KR051904 | Myanmar | 2014 | DENV2 |
| KJ470751 | Myanmar | 2014 | DENV2 |
| KX262955 | China-Yunnan | 2014 | DENV2 |
| KX262958 | China-Yunnan | 2014 | DENV2 |
| KY672948 | China-Yunnan | 2014 | DENV2 |
| KR051898 | Myanmar | 2014 | DENV2 |
| KR051899 | Myanmar | 2014 | DENV2 |
| KY038918 | China-Yunnan | 2014 | DENV2 |
| KX357988 | Myanmar | 2016 | DENV2 |
| KY586627 | Thailand | 2010 | DENV2 |
| KJ939381 | China-Yunnan | 2014 | DENV2 |
| KJ939407 | China-Yunnan | 2014 | DENV2 |
| KR051902 | Myanmar | 2014 | DENV2 |
| KJ470750 | China-Yunnan | 2014 | DENV2 |
| KJ939383 | China-Yunnan | 2014 | DENV2 |
| KJ939400 | China-Yunnan | 2014 | DENV2 |
| KJ470759 | Myanmar | 2014 | DENV2 |
| KJ939379 | China-Yunnan | 2014 | DENV2 |
| KR051901 | Myanmar | 2014 | DENV2 |
| KX270817 | China-Guangdong | 2011 | DENV2 |
| MG894984 | China-Taiwan | 2012 | DENV2 |
| KX357998 | Myanmar | 2016 | DENV2 |
| MG895026 | China-Taiwan | 2013 | DENV2 |
| KT781543 | Thailand | 2013 | DENV2 |
| KU509273 | Thailand | 2012 | DENV2 |
| JF968045 | Thailand | 2011 | DENV2 |
| JF968033 | Thailand | 2011 | DENV2 |
| MG895031 | China-Taiwan | 2014 | DENV2 |
| MG564091 | Thailand | 2011 | DENV2 |
| KY586629 | Thailand | 2007 | DENV2 |
| KY586630 | Thailand | 2007 | DENV2 |
| EU448416 | Thailand | 2007 | DENV2 |
| KR051900 | Myanmar | 2014 | DENV2 |
| MG895034 | China-Taiwan | 2014 | DENV2 |
| MG895012 | China-Taiwan | 2013 | DENV2 |
| MG895107 | China-Taiwan | 2016 | DENV2 |
| KY849764 | Laos | 2010 | DENV2 |
| KY849767 | Laos | 2011 | DENV2 |
| KY586624 | Thailand | 2009 | DENV2 |
| JN544398 | Singapore | 2012 | DENV2 |
| MG564085 | Thailand | 2010 | DENV2 |
| MG564095 | Thailand | 2012 | DENV2 |
| MG564088 | Thailand | 2011 | DENV2 |
| MG895007 | China-Taiwan | 2013 | DENV2 |
| JF968029 | Thailand | 2011 | DENV2 |
| KY586628 | Thailand | 2007 | DENV2 |
| JF968021 | Laos | 2011 | DENV2 |
| KM216697 | Laos | 2011 | DENV2 |
| JF968020 | Laos | 2011 | DENV2 |
| KY849766 | Laos | 2010 | DENV2 |
| JN568282 | Vietnam | 2011 | DENV2 |
| KY849763 | Laos | 2011 | DENV2 |
| JF967990 | Thailand | 2010 | DENV2 |
| KY586622 | Thailand | 2007 | DENV2 |
| KY586623 | Thailand | 2007 | DENV2 |
| KY586620 | Thailand | 2008 | DENV2 |
| KY586621 | Thailand | 2007 | DENV2 |
| JF968026 | Myanmar | 2011 | DENV2 |
| KY586625 | Thailand | 2011 | DENV2 |
| KY586631 | Thailand | 2003 | DENV2 |
| KY586632 | Thailand | 2004 | DENV2 |
| KY586635 | Thailand | 2002 | DENV2 |
| JQ993210 | Thailand | 2008 | DENV2 |
| JQ993212 | Thailand | 2008 | DENV2 |
| JQ993227 | Thailand | 2008 | DENV2 |
| JQ993213 | Thailand | 2008 | DENV2 |
| JQ993211 | Thailand | 2008 | DENV2 |
| JX093603 | Vietnam | 2012 | DENV2 |
| JX101615 | Vietnam | 2012 | DENV2 |
| JX093605 | Vietnam | 2012 | DENV2 |
| JQ993226 | Thailand | 2006 | DENV2 |
| DQ518646 | Thailand | 2005 | DENV2 |
| JQ993224 | Thailand | 2005 | DENV2 |
| JQ993220 | Thailand | 2005 | DENV2 |
| JQ993219 | Thailand | 2005 | DENV2 |
| AB194882 | Thailand | 2005 | DENV2 |
| KY586633 | Thailand | 2007 | DENV2 |
| MG564084 | Thailand | 2012 | DENV2 |
| MG564086 | Thailand | 2012 | DENV2 |
| KX147335 | Thailand | 2015 | DENV2 |
| EU448415 | Cambodia | 2008 | DENV2 |
| JQ403522 | China-Taiwan | 2009 | DENV2 |
| JF967987 | Cambodia | 2010 | DENV2 |
| KY586636 | Thailand | 2005 | DENV2 |
| KY586634 | Thailand | 2003 | DENV2 |
| LC147056 | Laos | 2014 | DENV2 |
| LC147057 | Laos | 2014 | DENV2 |
| JF968013 | Thailand | 2011 | DENV2 |
| KY586597 | Thailand | 2010 | DENV2 |
| JN568273 | Thailand | 2008 | DENV2 |
| KY849765 | Laos | 2010 | DENV2 |
| KY586596 | Thailand | 2008 | DENV2 |
| KY849768 | Laos | 2011 | DENV2 |
| KY586602 | Thailand | 2005 | DENV2 |
| KY586595 | Thailand | 2005 | DENV2 |
| KY586603 | Thailand | 2003 | DENV2 |
| KY586604 | Thailand | 2003 | DENV2 |
| KY586608 | Thailand | 2001 | DENV2 |
| KY586614 | Thailand | 2005 | DENV2 |
| KY586611 | Thailand | 2003 | DENV2 |
| KY586606 | Thailand | 2003 | DENV2 |
| KY586605 | Thailand | 2005 | DENV2 |
| KY586607 | Thailand | 2002 | DENV2 |
| KY586609 | Thailand | 2001 | DENV2 |
| KY586610 | Thailand | 2001 | DENV2 |
| KY586589 | Thailand | 2005 | DENV2 |
| KY586590 | Thailand | 2006 | DENV2 |
| DQ181797 | Thailand | 2002 | DENV2 |
| KY586639 | Thailand | 2001 | DENV2 |
| KY586640 | Thailand | 2005 | DENV2 |
| KY586638 | Thailand | 2002 | DENV2 |
| KY586637 | Thailand | 2002 | DENV2 |
| KY586619 | Thailand | 2001 | DENV2 |
| KY586591 | Thailand | 2003 | DENV2 |
| DQ181872 | Thailand | 2001 | DENV2 |
| KY586588 | Thailand | 2003 | DENV2 |
| KY586582 | Thailand | 2005 | DENV2 |
| KY586594 | Thailand | 2003 | DENV2 |
| KY586592 | Thailand | 2006 | DENV2 |
| KY586615 | Thailand | 2004 | DENV2 |
| KY586616 | Thailand | 2003 | DENV2 |
| KY586583 | Thailand | 2005 | DENV2 |
| MG895152 | China-Taiwan | 2017 | DENV2 |
| MG895161 | China-Taiwan | 2017 | DENV2 |
| LC410185 | Thailand | 2017 | DENV2 |
| MG895158 | China-Taiwan | 2017 | DENV2 |
| MN955663 | Thailand | 2018 | DENV2 |
| MG895105 | China-Taiwan | 2016 | DENV2 |
| KY495819 | Cambodia | 2016 | DENV2 |
| MW018160 | Cambodia | 2019 | DENV2 |
| MN955661 | Thailand | 2018 | DENV2 |
| MN955666 | Thailand | 2019 | DENV2 |
| MG895130 | China-Taiwan | 2016 | DENV2 |
| MG596014 | China-Guangdong | 2017 | DENV2 |
| MH010611 | China-Zhejiang | 2018 | DENV2 |
| MG895101 | China-Taiwan | 2016 | DENV2 |
| MH827538 | China-Guangdong | 2018 | DENV2 |
| MH010609 | China-Zhejiang | 2018 | DENV2 |
| MH010610 | China-Zhejiang | 2018 | DENV2 |
| MH010617 | China-Zhejiang | 2018 | DENV2 |
| MT252647 | Singapore | 2017 | DENV2 |
| MG895167 | China-Taiwan | 2017 | DENV2 |
| JX093643 | Vietnam | 2012 | DENV2 |
| KP191527 | China-Guangdong | 2015 | DENV2 |
| MH827531 | China-Guangdong | 2018 | DENV2 |
| KT175128 | Vietnam | 2013 | DENV2 |
| MG564093 | Thailand | 2012 | DENV2 |
| MN923135 | China-Guangdong | 2019 | DENV2 |
| KT781556 | Thailand | 2014 | DENV2 |
| KJ545457 | China-Guangdong/Foshan | 2014 | DENV2 |
| MG895052 | China-Taiwan | 2014 | DENV2 |
| KU291805 | China-Guangdong | 2014 | DENV2 |
| MG895043 | China-Taiwan | 2014 | DENV2 |
| MG564087 | Thailand | 2012 | DENV2 |
| MG564097 | Thailand | 2013 | DENV2 |
| MG564096 | Thailand | 2013 | DENV2 |
| MG564098 | Thailand | 2013 | DENV2 |
| MG895016 | China-Taiwan | 2013 | DENV2 |
| KX270813 | China-Guangdong | 2014 | DENV2 |
| MG894981 | China-Taiwan | 2012 | DENV2 |
| MG895029 | China-Taiwan | 2013 | DENV2 |
| JF967988 | Cambodia | 2010 | DENV2 |
| KX270810 | China-Guangdong | 2012 | DENV2 |
| KF955401 | Cambodia | 2009 | DENV2 |
| KT452795 | Cambodia | 2010 | DENV2 |
| JF967966 | Cambodia | 2009 | DENV2 |
| KJ806930 | Malaysia | 2015 | DENV2 |
| KJ806907 | Malaysia | 2014 | DENV2 |
| KJ806920 | Malaysia | 2014 | DENV2 |
| JF967989 | Cambodia | 2010 | DENV2 |
| GU908498 | Vietnam | 2010 | DENV2 |
| JN568274 | Thailand | 2011 | DENV2 |
| MG894971 | China-Taiwan | 2011 | DENV2 |
| KY586585 | Thailand | 2008 | DENV2 |
| KY586586 | Thailand | 2007 | DENV2 |
| JF968030 | Thailand | 2011 | DENV2 |
| JN568244 | Laos | 2011 | DENV2 |
| KY586587 | Thailand | 2009 | DENV2 |
| KY586584 | Thailand | 2004 | DENV2 |
| GQ357789 | Singapore | 2009 | DENV2 |
| KX357976 | Myanmar | 2016 | DENV2 |
| DQ181799 | Thailand | 1999 | DENV2 |
| KX357993 | Myanmar | 2016 | DENV2 |
| KX357997 | Myanmar | 2016 | DENV2 |
| KX357996 | Myanmar | 2016 | DENV2 |
| KX224266 | Singapore | 2015 | DENV2 |
| KR051907 | Myanmar | 2014 | DENV2 |
| KR051906 | Myanmar | 2014 | DENV2 |
| KX262933 | China-Yunnan | 2016 | DENV2 |
| KR051905 | Myanmar | 2014 | DENV2 |
| MG564102 | Thailand | 2016 | DENV2 |
| KX357985 | Myanmar | 2016 | DENV2 |
| KX357987 | Myanmar | 2016 | DENV2 |
| KT175137 | Myanmar | 2015 | DENV2 |
| KX262931 | China-Yunnan | 2016 | DENV2 |
| KX357986 | Myanmar | 2016 | DENV2 |
| KX357981 | Myanmar | 2016 | DENV2 |
| KX357983 | Myanmar | 2016 | DENV2 |
| KX357989 | Myanmar | 2016 | DENV2 |
| KX357984 | Myanmar | 2016 | DENV2 |
| KY038909 | China-Yunnan | 2015 | DENV2 |
| KX270818 | China-Guangdong | 2011 | DENV2 |
| KY586695 | Thailand | 2006 | DENV2 |
| KY586696 | Thailand | 2005 | DENV2 |
| KY586697 | Thailand | 2005 | DENV2 |
| KY586698 | Thailand | 2005 | DENV2 |
| DQ518651 | Myanmar | 2003 | DENV2 |
| DQ518652 | Myanmar | 2005 | DENV2 |
| DQ181892 | Thailand | 2001 | DENV2 |
| DQ181891 | Thailand | 2001 | DENV2 |
| DQ518653 | Myanmar | 2005 | DENV2 |
| DQ181854 | Thailand | 1994 | DENV2 |
| DQ181886 | Thailand | 1994 | DENV2 |
| DQ181882 | Thailand | 1995 | DENV2 |
| DQ181885 | Thailand | 1994 | DENV2 |
| DQ181881 | Thailand | 1996 | DENV2 |
| DQ181865 | Thailand | 1997 | DENV2 |
| KY586694 | Thailand | 1996 | DENV2 |
| DQ181901 | Thailand | 1997 | DENV2 |
| DQ181897 | Thailand | 1999 | DENV2 |
| DQ181899 | Thailand | 1998 | DENV2 |
| KY586690 | Thailand | 2000 | DENV2 |
| KY586691 | Thailand | 1999 | DENV2 |
| KY586693 | Thailand | 1996 | DENV2 |
| DQ181870 | Thailand | 1996 | DENV2 |
| DQ181875 | Thailand | 2000 | DENV2 |
| KY586692 | Thailand | 2002 | DENV2 |
| DQ181829 | Thailand | 1988 | DENV2 |
| DQ181887 | Thailand | 1991 | DENV2 |
| DQ181861 | Thailand | 1999 | DENV2 |
| DQ181867 | Thailand | 1997 | DENV2 |
| KY586689 | Thailand | 1996 | DENV2 |
| DQ181884 | Thailand | 1994 | DENV2 |
| DQ181802 | Thailand | 1989 | DENV2 |
| DQ181847 | Thailand | 1989 | DENV2 |
| DQ181848 | Thailand | 1988 | DENV2 |
| DQ181840 | Thailand | 1992 | DENV2 |
| DQ181798 | Thailand | 2000 | DENV2 |
| DQ181803 | Thailand | 1986 | DENV2 |
| DQ181805 | Thailand | 1980 | DENV2 |
| DQ181804 | Thailand | 1985 | DENV2 |
| DQ181806 | Thailand | 1975 | DENV2 |
| GU289914 | Thailand | 1975 | DENV2 |
| AJ487271 | Thailand | 1975 | DENV2 |
| JX649147 | Vietnam | 1996 | DENV2 |
| JX649148 | Vietnam | 1996 | DENV2 |
| FM210217 | Vietnam | 2000 | DENV2 |
| DQ181801 | Thailand | 1991 | DENV2 |
| KR347419 | China-Yunnan | 2014 | DENV3 |
| KR347360 | China-Yunnan | 2014 | DENV3 |
| KR347380 | China-Yunnan | 2014 | DENV3 |
| KR347367 | China-Yunnan | 2014 | DENV3 |
| KR347370 | China-Yunnan | 2014 | DENV3 |
| KX262919 | China-Yunnan | 2014 | DENV3 |
| KR347417 | China-Yunnan | 2014 | DENV3 |
| KR347432 | China-Yunnan | 2014 | DENV3 |
| KR296743 | China-Yunnan | 2014 | DENV3 |
| KY038920 | China-Yunnan | 2014 | DENV3 |
| KR347366 | China-Yunnan | 2014 | DENV3 |
| KR347423 | China-Yunnan | 2014 | DENV3 |
| KR347400 | China-Yunnan | 2014 | DENV3 |
| KR347398 | China-Yunnan | 2014 | DENV3 |
| KR347409 | China-Yunnan | 2014 | DENV3 |
| KR347435 | China-Yunnan | 2014 | DENV3 |
| KR347393 | China-Yunnan | 2014 | DENV3 |
| KR347365 | China-Yunnan | 2014 | DENV3 |
| KR347418 | China-Yunnan | 2014 | DENV3 |
| KR347422 | China-Yunnan | 2014 | DENV3 |
| KR347378 | China-Yunnan | 2014 | DENV3 |
| KR347397 | China-Yunnan | 2014 | DENV3 |
| KR347394 | China-Yunnan | 2014 | DENV3 |
| KR347382 | China-Yunnan | 2014 | DENV3 |
| KR347373 | China-Yunnan | 2014 | DENV3 |
| KR347375 | China-Yunnan | 2014 | DENV3 |
| KF816153 | China-Yunnan | 2014 | DENV3 |
| KR347433 | China-Yunnan | 2014 | DENV3 |
| KR347431 | China-Yunnan | 2014 | DENV3 |
| KX262914 | China-Yunnan | 2014 | DENV3 |
| KF816156 | China-Yunnan | 2014 | DENV3 |
| KM651771 | China-Yunnan | 2014 | DENV3 |
| KX262916 | China-Yunnan | 2014 | DENV3 |
| KR347361 | China-Yunnan | 2014 | DENV3 |
| KR347415 | China-Yunnan | 2014 | DENV3 |
| KR347424 | China-Yunnan | 2014 | DENV3 |
| KR347425 | China-Yunnan | 2014 | DENV3 |
| KR347426 | China-Yunnan | 2014 | DENV3 |
| KR347436 | China-Yunnan | 2014 | DENV3 |
| KR347420 | China-Yunnan | 2014 | DENV3 |
| KM651778 | China-Yunnan | 2014 | DENV3 |
| KR347437 | China-Yunnan | 2014 | DENV3 |
| KR347428 | China-Yunnan | 2014 | DENV3 |
| KR347429 | China-Yunnan | 2014 | DENV3 |
| KR347408 | China-Yunnan | 2014 | DENV3 |
| KR347368 | China-Yunnan | 2014 | DENV3 |
| KR347390 | China-Yunnan | 2014 | DENV3 |
| KX262918 | China-Yunnan | 2014 | DENV3 |
| MN018369 | China-Guangdong | 2014 | DENV3 |
| HG530167 | Laos | 2012 | DENV3 |
| KJ622199 | China-Henan | 2014 | DENV3 |
| KJ622193 | China-Henan | 2014 | DENV3 |
| KJ622194 | China-Henan | 2014 | DENV3 |
| KJ622195 | China-Henan | 2014 | DENV3 |
| KJ622191 | China-Henan | 2014 | DENV3 |
| HG530176 | Laos | 2013 | DENV3 |
| HG530195 | Laos | 2013 | DENV3 |
| KF816158 | Laos | 2014 | DENV3 |
| HG530154 | Laos | 2013 | DENV3 |
| LN680425 | Laos | 2013 | DENV3 |
| HG530148 | Laos | 2013 | DENV3 |
| HG530155 | Laos | 2013 | DENV3 |
| HG530182 | Laos | 2013 | DENV3 |
| HG530201 | Laos | 2013 | DENV3 |
| HG530141 | Laos | 2013 | DENV3 |
| LC147061 | Laos | 2014 | DENV3 |
| LC147059 | Laos | 2014 | DENV3 |
| HG530145 | Laos | 2013 | DENV3 |
| HG530150 | Laos | 2013 | DENV3 |
| HG530187 | Laos | 2013 | DENV3 |
| HG530175 | Laos | 2013 | DENV3 |
| HG530194 | Laos | 2013 | DENV3 |
| HG530147 | Laos | 2013 | DENV3 |
| HG530163 | Laos | 2013 | DENV3 |
| LN680426 | Laos | 2013 | DENV3 |
| KY849769 | Laos | 2011 | DENV3 |
| KY849775 | Laos | 2011 | DENV3 |
| KY849774 | Laos | 2011 | DENV3 |
| KY849771 | Laos | 2010 | DENV3 |
| HG530199 | Laos | 2013 | DENV3 |
| KR347387 | China-Yunnan | 2014 | DENV3 |
| KR347407 | China-Yunnan | 2014 | DENV3 |
| KF816160 | Laos | 2014 | DENV3 |
| MG895202 | China-Taiwan | 2014 | DENV3 |
| HG530162 | Laos | 2013 | DENV3 |
| HG530200 | Laos | 2013 | DENV3 |
| JF968066 | Myanmar | 2009 | DENV3 |
| KY586805 | Thailand | 2009 | DENV3 |
| KY586804 | Thailand | 2008 | DENV3 |
| KT758790 | Thailand | 2014 | DENV3 |
| KY586808 | Thailand | 2011 | DENV3 |
| KT758741 | Thailand | 2012 | DENV3 |
| GQ357834 | Singapore | 2009 | DENV3 |
| KY586815 | Thailand | 2007 | DENV3 |
| KY586816 | Thailand | 2007 | DENV3 |
| EU448443 | Myanmar | 2008 | DENV3 |
| KT424097 | Thailand | 2015 | DENV3 |
| KY586810 | Thailand | 2010 | DENV3 |
| KY586813 | Thailand | 2010 | DENV3 |
| JF968088 | Myanmar | 2010 | DENV3 |
| KT452792 | Myanmar | 2009 | DENV3 |
| KY586811 | Thailand | 2010 | DENV3 |
| KY586812 | Thailand | 2011 | DENV3 |
| EU448444 | Myanmar | 2007 | DENV3 |
| EU478409 | Myanmar | 2007 | DENV3 |
| KY586817 | Thailand | 2007 | DENV3 |
| MG564106 | Thailand | 2012 | DENV3 |
| DQ518666 | Myanmar | 2006 | DENV3 |
| KY586803 | Thailand | 2008 | DENV3 |
| JN030196 | Singapore | 2009 | DENV3 |
| JN036387 | Bangladesh | 2007 | DENV3 |
| JN036386 | Bangladesh | 2008 | DENV3 |
| JN030195 | Singapore | 2010 | DENV3 |
| JN036381 | Bangladesh | 2010 | DENV3 |
| JF968064 | Bangladesh | 2009 | DENV3 |
| JN036384 | Bangladesh | 2009 | DENV3 |
| JN036385 | Bangladesh | 2008 | DENV3 |
| JN030198 | Singapore | 2009 | DENV3 |
| JN036392 | Bangladesh | 2007 | DENV3 |
| EU448445 | Bangladesh | 2008 | DENV3 |
| EU448446 | Bangladesh | 2007 | DENV3 |
| JN036389 | Bangladesh | 2007 | DENV3 |
| JN036390 | Bangladesh | 2007 | DENV3 |
| JN036394 | Bangladesh | 2007 | DENV3 |
| JN036393 | Bangladesh | 2007 | DENV3 |
| JN036383 | Bangladesh | 2009 | DENV3 |
| JN036395 | Bangladesh | 2007 | DENV3 |
| JN036382 | Bangladesh | 2009 | DENV3 |
| JN036391 | Bangladesh | 2007 | DENV3 |
| JF968085 | Bangladesh | 2010 | DENV3 |
| AY656670 | Bangladesh | 2001 | DENV3 |
| AY656672 | Bangladesh | 2002 | DENV3 |
| AY656674 | Bangladesh | 2001 | DENV3 |
| EU448447 | Myanmar | 1999 | DENV3 |
| AY656671 | Bangladesh | 2001 | DENV3 |
| DQ518665 | Bangladesh | 2002 | DENV3 |
| DQ675528 | China-Taiwan | 1999 | DENV3 |
| KT758748 | Thailand | 2013 | DENV3 |
| MG564117 | Thailand | 2016 | DENV3 |
| AY923865 | Thailand | 1995 | DENV3 |
| KJ737429 | Thailand | 1995 | DENV3 |
| AY676348 | Thailand | 1999 | DENV3 |
| MG895205 | China-Taiwan | 2014 | DENV3 |
| AY676349 | Thailand | 1999 | DENV3 |
| AY676350 | Thailand | 1994 | DENV3 |
| AY676351 | Thailand | 1994 | DENV3 |
| AY876494 | Thailand | 1995 | DENV3 |
| DQ675520 | Indonesia | 1999 | DENV3 |
| AY676353 | Thailand | 1988 | DENV3 |
| KJ737430 | Thailand | 1984 | DENV3 |
| AY676352 | Thailand | 1988 | DENV3 |
| KU509302 | Thailand | 2014 | DENV3 |
| KU509303 | Thailand | 2014 | DENV3 |
| KP176710 | Vietnam | 2014 | DENV3 |
| LN680429 | Laos | 2013 | DENV3 |
| LN680428 | Laos | 2013 | DENV3 |
| KF816163 | Laos | 2014 | DENV3 |
| MF370226 | China-Yunnan | 2014 | DENV3 |
| KP176712 | Vietnam | 2014 | DENV3 |
| KP176711 | Vietnam | 2014 | DENV3 |
| KP176713 | Vietnam | 2014 | DENV3 |
| MG564119 | Thailand | 2016 | DENV3 |
| MG895240 | China-Taiwan | 2016 | DENV3 |
| KU509304 | Thailand | 2015 | DENV3 |
| KY234176 | Thailand | 2016 | DENV3 |
| KY234178 | Thailand | 2016 | DENV3 |
| KY234177 | Thailand | 2016 | DENV3 |
| KY234179 | Thailand | 2016 | DENV3 |
| MF142763 | Thailand | 2016 | DENV3 |
| KY234173 | Thailand | 2013 | DENV3 |
| MG564114 | Thailand | 2016 | DENV3 |
| MG895241 | China-Taiwan | 2016 | DENV3 |
| MG895247 | China-Taiwan | 2016 | DENV3 |
| KX224286 | Singapore | 2015 | DENV3 |
| MG564127 | Thailand | 2016 | DENV3 |
| MG564128 | Thailand | 2016 | DENV3 |
| MG564126 | Thailand | 2016 | DENV3 |
| LC410193 | Thailand | 2017 | DENV3 |
| LC410195 | Thailand | 2017 | DENV3 |
| KP176707 | Thailand | 2013 | DENV3 |
| KP176715 | China-Taiwan | 2013 | DENV3 |
| MG564125 | Thailand | 2016 | DENV3 |
| KT758771 | Thailand | 2014 | DENV3 |
| LC410194 | Thailand | 2017 | DENV3 |
| KY495823 | Thailand | 2016 | DENV3 |
| KP176708 | Thailand | 2014 | DENV3 |
| MG564107 | Thailand | 2016 | DENV3 |
| MG895223 | China-Taiwan | 2015 | DENV3 |
| MG564118 | Thailand | 2016 | DENV3 |
| MN018378 | China-Guangdong | 2016 | DENV3 |
| MK780885 | Thailand | 2016 | DENV3 |
| MK780884 | Thailand | 2016 | DENV3 |
| MG895245 | China-Taiwan | 2016 | DENV3 |
| MG895235 | China-Taiwan | 2016 | DENV3 |
| MG564116 | Thailand | 2016 | DENV3 |
| MG564121 | Thailand | 2016 | DENV3 |
| MN922036 | China-Guangdong | 2020 | DENV3 |
| MG895265 | China-Taiwan | 2016 | DENV3 |
| MN018381 | China-Guangdong | 2016 | DENV3 |
| MN018372 | China-Guangdong | 2016 | DENV3 |
| MN018375 | China-Guangdong | 2016 | DENV3 |
| MN018371 | China-Guangdong | 2016 | DENV3 |
| MN018367 | China-Guangdong | 2016 | DENV3 |
| MN018376 | China-Guangdong | 2016 | DENV3 |
| MG895242 | China-Taiwan | 2016 | DENV3 |
| MG895252 | China-Taiwan | 2016 | DENV3 |
| MG895249 | China-Taiwan | 2016 | DENV3 |
| MG895232 | China-Taiwan | 2015 | DENV3 |
| KY921907 | Singapore | 2015 | DENV3 |
| MG895244 | China-Taiwan | 2016 | DENV3 |
| MG933847 | China-Yunnan | 2018 | DENV3 |
| MG933849 | China-Yunnan | 2018 | DENV3 |
| MG933848 | China-Yunnan | 2018 | DENV3 |
| MG564124 | Thailand | 2016 | DENV3 |
| MT815453 | China-Yunnan | 2020 | DENV3 |
| MG895243 | China-Taiwan | 2016 | DENV3 |
| MG895284 | China-Taiwan | 2017 | DENV3 |
| MG895254 | China-Taiwan | 2016 | DENV3 |
| MG895271 | China-Taiwan | 2017 | DENV3 |
| MG895251 | China-Taiwan | 2016 | DENV3 |
| MK780886 | Thailand | 2016 | DENV3 |
| KX357894 | Myanmar | 2016 | DENV3 |
| MG564123 | Thailand | 2016 | DENV3 |
| MK780887 | Thailand | 2016 | DENV3 |
| KT758772 | Singapore | 2014 | DENV3 |
| MG564110 | Thailand | 2014 | DENV3 |
| KT758784 | Thailand | 2016 | DENV3 |
| KX224278 | Singapore | 2014 | DENV3 |
| MG564104 | Thailand | 2012 | DENV3 |
| JF968098 | Thailand | 2011 | DENV3 |
| KP893719 | Vietnam | 2014 | DENV3 |
| GQ357856 | Singapore | 2009 | DENV3 |
| JF968092 | Thailand | 2011 | DENV3 |
| KY586822 | Thailand | 2010 | DENV3 |
| KY586819 | Thailand | 2008 | DENV3 |
| KY586820 | Thailand | 2009 | DENV3 |
| KT758769 | India | 2014 | DENV3 |
| MG721064 | India | 2017 | DENV3 |
| KU216209 | India | 2014 | DENV3 |
| KX270820 | China-Guangdong | 2012 | DENV3 |
| KX224282 | Singapore | 2014 | DENV3 |
| MH428211 | Singapore | 2014 | DENV3 |
| KP176706 | India | 2014 | DENV3 |
| JF968097 | India | 2011 | DENV3 |
| KP176705 | India | 2012 | DENV3 |
| JQ922557 | India | 2006 | DENV3 |
| JQ686078 | India | 2006 | DENV3 |
| JQ922556 | India | 2006 | DENV3 |
| KM217159 | Pakistan | 2014 | DENV3 |
| KF041258 | Pakistan | 2010 | DENV3 |
| KF041252 | Pakistan | 2010 | DENV3 |
| KF041251 | Pakistan | 2010 | DENV3 |
| KF041254 | Pakistan | 2009 | DENV3 |
| KT239362 | Pakistan | 2015 | DENV3 |
| MK894339 | China-Guangdong | 2018 | DENV3 |
| KF041250 | Pakistan | 2007 | DENV3 |
| KF041257 | Pakistan | 2007 | DENV3 |
| KF041243 | Pakistan | 2007 | DENV3 |
| KF041256 | Pakistan | 2007 | DENV3 |
| KF041248 | Pakistan | 2007 | DENV3 |
| KT239360 | Pakistan | 2015 | DENV3 |
| KF041241 | Pakistan | 2008 | DENV3 |
| KF041245 | Pakistan | 2010 | DENV3 |
| KF041253 | Pakistan | 2007 | DENV3 |
| KT239357 | Pakistan | 2015 | DENV3 |
| KF041247 | Pakistan | 2007 | DENV3 |
| KT239361 | Pakistan | 2015 | DENV3 |
| KF041246 | Pakistan | 2007 | DENV3 |
| KF041242 | Pakistan | 2007 | DENV3 |
| KF041255 | Pakistan | 2008 | DENV3 |
| KX427164 | India | 2016 | DENV3 |
| KF041259 | Pakistan | 2007 | DENV3 |
| KF041249 | Pakistan | 2007 | DENV3 |
| KF041238 | Pakistan | 2007 | DENV3 |
| KF041240 | Pakistan | 2007 | DENV3 |
| KF041244 | Pakistan | 2007 | DENV3 |
| KF041239 | Pakistan | 2007 | DENV3 |
| JQ686072 | India | 2010 | DENV3 |
| JQ686073 | India | 2010 | DENV3 |
| JQ686071 | India | 2010 | DENV3 |
| JQ686074 | India | 2011 | DENV3 |
| JQ686075 | India | 2011 | DENV3 |
| JQ686076 | India | 2011 | DENV3 |
| KT758752 | India | 2013 | DENV3 |
| MN964273 | China-Guangdong | 2020 | DENV3 |
| MH010603 | China-Zhejiang | 2018 | DENV3 |
| GU363549 | China-Guangdong | 2010 | DENV3 |
| KX387842 | India | 2016 | DENV3 |
| FJ644564 | India | 2008 | DENV3 |
| GQ466079 | India | 2009 | DENV3 |
| MH594938 | India | 2018 | DENV3 |
| MH594946 | India | 2018 | DENV3 |
| MH594947 | India | 2018 | DENV3 |
| MH594931 | India | 2018 | DENV3 |
| MH594944 | India | 2018 | DENV3 |
| MH594933 | India | 2018 | DENV3 |
| MH594940 | India | 2018 | DENV3 |
| MH594941 | India | 2018 | DENV3 |
| MN253128 | India | 2019 | DENV3 |
| MT224916 | India | 2018 | DENV3 |
| MT224917 | India | 2018 | DENV3 |
| MN253131 | India | 2019 | DENV3 |
| MH594948 | India | 2018 | DENV3 |
| MH594937 | India | 2018 | DENV3 |
| MK858154 | India | 2018 | DENV3 |
| MH891766 | India | 2017 | DENV3 |
| MN244926 | India | 2018 | DENV3 |
| MH594939 | India | 2018 | DENV3 |
| MH594943 | India | 2018 | DENV3 |
| MN253124 | India | 2018 | DENV3 |
| MH594942 | India | 2018 | DENV3 |
| MN964274 | China-Guangdong | 2020 | DENV3 |
| MN915213 | China-Guangdong | 2020 | DENV3 |
| MN227700 | China-Guangdong | 2020 | DENV3 |
| MN227699 | China-Guangdong | 2020 | DENV3 |
| MN913503 | China-Guangdong | 2020 | DENV3 |
| MN915219 | China-Guangdong | 2020 | DENV3 |
| MN915211 | China-Guangdong | 2020 | DENV3 |
| MN915198 | China-Guangdong | 2019 | DENV3 |
| MK894340 | China-Guangdong | 2019 | DENV3 |
| MH594936 | India | 2018 | DENV3 |
| MH594949 | India | 2018 | DENV3 |
| MN018385 | China-Guangdong | 2017 | DENV3 |
| MK858153 | India | 2017 | DENV3 |
| MK858155 | India | 2018 | DENV3 |
| KX224293 | Singapore | 2014 | DENV3 |
| MH428213 | Singapore | 2014 | DENV3 |
| MH428212 | Singapore | 2014 | DENV3 |
| MH594934 | India | 2018 | DENV3 |
| KF954947 | China-Guangdong | 2014 | DENV3 |
| MN018386 | China-Guangdong | 2014 | DENV3 |
| KF954949 | China-Guangdong | 2014 | DENV3 |
| KF954946 | China-Guangdong | 2014 | DENV3 |
| KF954948 | China-Guangdong | 2014 | DENV3 |
| KU291644 | China-Guangdong | 2014 | DENV3 |
| KJ545474 | China-Guangdong | 2014 | DENV3 |
| KJ545473 | China-Guangdong | 2014 | DENV3 |
| KX224271 | Singapore | 2014 | DENV3 |
| KX224273 | Singapore | 2014 | DENV3 |
| KX224274 | Singapore | 2014 | DENV3 |
| MH594932 | India | 2018 | DENV3 |
| KX380841 | Singapore | 2013 | DENV3 |
| KY978445 | India | 2013 | DENV3 |
| MK858150 | India | 2017 | DENV3 |
| MT224921 | India | 2015 | DENV3 |
| MK858152 | India | 2017 | DENV3 |
| MG721061 | India | 2017 | DENV3 |
| KX224279 | Singapore | 2014 | DENV3 |
| JQ686080 | India | 2011 | DENV3 |
| JQ686081 | India | 2010 | DENV3 |
| JN030194 | Singapore | 2008 | DENV3 |
| JQ686070 | India | 2011 | DENV3 |
| MT224915 | India | 2018 | DENV3 |
| MT224918 | India | 2017 | DENV3 |
| MT224920 | India | 2016 | DENV3 |
| MT224919 | India | 2016 | DENV3 |
| KX420603 | India | 2016 | DENV3 |
| MH822957 | India | 2014 | DENV3 |
| MH594935 | India | 2018 | DENV3 |
| MH594945 | India | 2018 | DENV3 |
| KP176704 | India | 2012 | DENV3 |
| KP176703 | India | 2012 | DENV3 |
| KU509281 | India | 2010 | DENV3 |
| KX855927 | India | 2015 | DENV3 |
| MK858149 | India | 2017 | DENV3 |
| JQ686082 | India | 2010 | DENV3 |
| KY586821 | Thailand | 2011 | DENV3 |
| DQ675533 | China-Taiwan | 2000 | DENV3 |
| MK780888 | Thailand | 2017 | DENV3 |
| KY072811 | East Timor | 2013 | DENV3 |
| KY072815 | East Timor | 2012 | DENV3 |
| KY275242 | East Timor | 2012 | DENV3 |
| KY072813 | East Timor | 2013 | DENV3 |
| KY072810 | East Timor | 2013 | DENV3 |
| KY275236 | East Timor | 2012 | DENV3 |
| MH036413 | Indonesia | 2017 | DENV3 |
| JF968091 | Indonesia | 2011 | DENV3 |
| KU509285 | Thailand | 2011 | DENV3 |
| MG895214 | China-Taiwan | 2014 | DENV3 |
| MK629489 | Indonesia | 2015 | DENV3 |
| MG895230 | China-Taiwan | 2015 | DENV3 |
| MG895259 | China-Taiwan | 2016 | DENV3 |
| MK629488 | Indonesia | 2014 | DENV3 |
| MG895228 | China-Taiwan | 2015 | DENV3 |
| MG895233 | China-Taiwan | 2015 | DENV3 |
| MH173166 | Indonesia | 2017 | DENV3 |
| MN018384 | China-Guangdong | 2017 | DENV3 |
| JF968104 | Indonesia | 2011 | DENV3 |
| JF968095 | Indonesia | 2011 | DENV3 |
| KM216737 | Indonesia | 2011 | DENV3 |
| KT758774 | Malaysia | 2015 | DENV3 |
| KX224285 | Singapore | 2015 | DENV3 |
| MG895209 | China-Taiwan | 2014 | DENV3 |
| MH729988 | China-Fujian | 2017 | DENV3 |
| KY709191 | Indonesia | 2016 | DENV3 |
| MG895229 | China-Taiwan | 2015 | DENV3 |
| MG895189 | China-Taiwan | 2013 | DENV3 |
| MG895281 | China-Taiwan | 2017 | DENV3 |
| MK629491 | Indonesia | 2014 | DENV3 |
| GQ357842 | Singapore | 2008 | DENV3 |
| MK780880 | Thailand | 2016 | DENV3 |
| MK780881 | Thailand | 2016 | DENV3 |
| MK780882 | Thailand | 2016 | DENV3 |
| KY709189 | Indonesia | 2016 | DENV3 |
| KJ184317 | Indonesia | 2013 | DENV3 |
| MG895210 | China-Taiwan | 2014 | DENV3 |
| KY709193 | Indonesia | 2016 | DENV3 |
| MN018374 | China-Guangdong | 2016 | DENV3 |
| MG895286 | China-Taiwan | 2017 | DENV3 |
| MG895200 | China-Taiwan | 2014 | DENV3 |
| MG895203 | China-Taiwan | 2014 | DENV3 |
| MK629490 | Indonesia | 2015 | DENV3 |
| JN030179 | Singapore | 2011 | DENV3 |
| MG895195 | China-Taiwan | 2014 | DENV3 |
| MN018373 | China-Guangdong | 2017 | DENV3 |
| JF968078 | Indonesia | 2010 | DENV3 |
| KT204458 | Indonesia | 2013 | DENV3 |
| MK629487 | Indonesia | 2017 | DENV3 |
| MK629485 | Indonesia | 2017 | DENV3 |
| MK629486 | Indonesia | 2015 | DENV3 |
| KF709426 | Indonesia | 2014 | DENV3 |
| JN030190 | Singapore | 2011 | DENV3 |
| JN575567 | Indonesia | 2009 | DENV3 |
| JN030181 | Singapore | 2011 | DENV3 |
| JN030188 | Singapore | 2011 | DENV3 |
| JN030186 | Singapore | 2011 | DENV3 |
| KT204463 | Indonesia | 2011 | DENV3 |
| JN568283 | Indonesia | 2011 | DENV3 |
| JN575560 | Indonesia | 2011 | DENV3 |
| GQ357839 | Singapore | 2007 | DENV3 |
| KC762684 | Indonesia | 2008 | DENV3 |
| KT758757 | Indonesia | 2014 | DENV3 |
| MG895191 | China-Taiwan | 2013 | DENV3 |
| KC589012 | Indonesia | 2012 | DENV3 |
| KC589013 | Indonesia | 2012 | DENV3 |
| MK629482 | Indonesia | 2014 | DENV3 |
| JF968102 | Indonesia | 2011 | DENV3 |
| KT758773 | Indonesia | 2015 | DENV3 |
| MK629483 | Indonesia | 2017 | DENV3 |
| MN018370 | China-Guangdong | 2016 | DENV3 |
| MG895212 | China-Taiwan | 2014 | DENV3 |
| KY216160 | Indonesia | 2015 | DENV3 |
| EU448434 | Indonesia | 2008 | DENV3 |
| MG895192 | China-Taiwan | 2013 | DENV3 |
| MG895219 | China-Taiwan | 2015 | DENV3 |
| MG895246 | China-Taiwan | 2016 | DENV3 |
| EU448435 | Indonesia | 2007 | DENV3 |
| AY858042 | Indonesia | 2005 | DENV3 |
| AY858048 | Indonesia | 2005 | DENV3 |
| AY858047 | Indonesia | 2005 | DENV3 |
| KU291642 | China-Guangdong | 2014 | DENV3 |
| MG895176 | China-Taiwan | 2012 | DENV3 |
| JF968112 | Malaysia | 2011 | DENV3 |
| JF968105 | Indonesia | 2011 | DENV3 |
| MH036415 | Indonesia | 2017 | DENV3 |
| MG895226 | China-Taiwan | 2015 | DENV3 |
| KX646387 | Indonesia | 2010 | DENV3 |
| JF968072 | Indonesia | 2010 | DENV3 |
| JN575561 | Indonesia | 2011 | DENV3 |
| JF968090 | Indonesia | 2010 | DENV3 |
| MG895213 | China-Taiwan | 2014 | DENV3 |
| KY709190 | Indonesia | 2016 | DENV3 |
| KY709192 | Indonesia | 2016 | DENV3 |
| MG895288 | China-Taiwan | 2017 | DENV3 |
| MG895225 | China-Taiwan | 2015 | DENV3 |
| MG895224 | China-Taiwan | 2015 | DENV3 |
| KT758754 | Indonesia | 2014 | DENV3 |
| MG895193 | China-Taiwan | 2013 | DENV3 |
| KT758755 | Indonesia | 2014 | DENV3 |
| KX270819 | China-Guangdong | 2013 | DENV3 |
| KT758744 | Indonesia | 2013 | DENV3 |
| MG895187 | China-Taiwan | 2013 | DENV3 |
| KT758756 | Indonesia | 2014 | DENV3 |
| MG895178 | China-Taiwan | 2012 | DENV3 |
| KY863456 | Indonesia | 2017 | DENV3 |
| MG895181 | China-Taiwan | 2012 | DENV3 |
| MG895198 | China-Taiwan | 2014 | DENV3 |
| MK629484 | Indonesia | 2015 | DENV3 |
| MG895260 | China-Taiwan | 2016 | DENV3 |
| DQ518676 | Indonesia | 1999 | DENV3 |
| GQ357855 | Singapore | 2009 | DENV3 |
| JN030164 | Singapore | 2010 | DENV3 |
| JN030162 | Singapore | 2010 | DENV3 |
| GQ357853 | Singapore | 2009 | DENV3 |
| JN196615 | Singapore | 2010 | DENV3 |
| GQ357843 | Singapore | 2006 | DENV3 |
| GQ357844 | Singapore | 2006 | DENV3 |
| KX380840 | Singapore | 2013 | DENV3 |
| EU081221 | Singapore | 2006 | DENV3 |
| EU081223 | Singapore | 2006 | DENV3 |
| GQ357837 | Singapore | 2006 | DENV3 |
| EU448438 | Malaysia | 2008 | DENV3 |
| EU448439 | Malaysia | 2007 | DENV3 |
| GQ357838 | Singapore | 2007 | DENV3 |
| GQ357840 | Singapore | 2008 | DENV3 |
| JN030191 | Singapore | 2011 | DENV3 |
| AB189125 | Indonesia | 1999 | DENV3 |
| MH036402 | Indonesia | 2017 | DENV3 |
| MH036403 | Indonesia | 2017 | DENV3 |
| MH036410 | Indonesia | 2017 | DENV3 |
| MH036411 | Indonesia | 2017 | DENV3 |
| MH036404 | Indonesia | 2017 | DENV3 |
| MH036405 | Indonesia | 2017 | DENV3 |
| MK629475 | Indonesia | 2016 | DENV3 |
| MH036412 | Indonesia | 2017 | DENV3 |
| MH036398 | Indonesia | 2017 | DENV3 |
| MT377733 | Indonesia | 2019 | DENV3 |
| MH036406 | Indonesia | 2016 | DENV3 |
| MH823209 | Indonesia | 2017 | DENV3 |
| MG895293 | China-Taiwan | 2017 | DENV3 |
| MG895227 | China-Taiwan | 2015 | DENV3 |
| MG895264 | China-Taiwan | 2016 | DENV3 |
| MH173170 | Indonesia | 2017 | DENV3 |
| MH173171 | Indonesia | 2017 | DENV3 |
| MT006158 | Sri Lanka | 2019 | DENV3 |
| MT006169 | Sri Lanka | 2019 | DENV3 |
| MN083246 | Sri Lanka | 2018 | DENV3 |
| MG895273 | China-Taiwan | 2017 | DENV3 |
| MG895282 | China-Taiwan | 2017 | DENV3 |
| KY921906 | Singapore | 2016 | DENV3 |
| MH036401 | Indonesia | 2017 | DENV3 |
| MT377731 | Indonesia | 2019 | DENV3 |
| MT377732 | Indonesia | 2019 | DENV3 |
| MT377730 | Indonesia | 2019 | DENV3 |
| MG895279 | China-Taiwan | 2017 | DENV3 |
| MG895278 | China-Taiwan | 2017 | DENV3 |
| MG895196 | China-Taiwan | 2014 | DENV3 |
| KF709425 | Indonesia | 2014 | DENV3 |
| MN922040 | China-Guangdong | 2020 | DENV3 |
| MN922041 | China-Guangdong | 2020 | DENV3 |
| MN018387 | China-Guangdong | 2018 | DENV3 |
| MH594462 | Vietnam | 2018 | DENV3 |
| MG933850 | China-Yunnan | 2018 | DENV3 |
| MG895290 | China-Taiwan | 2017 | DENV3 |
| KY006150 | Indonesia | 2016 | DENV3 |
| MG895236 | China-Taiwan | 2016 | DENV3 |
| KY006145 | Indonesia | 2016 | DENV3 |
| KY006151 | Indonesia | 2016 | DENV3 |
| MK894338 | China-Guangdong | 2018 | DENV3 |
| MG895256 | China-Taiwan | 2016 | DENV3 |
| MT377734 | Indonesia | 2020 | DENV3 |
| MH036409 | Indonesia | 2017 | DENV3 |
| MH036414 | Indonesia | 2016 | DENV3 |
| MK629476 | Indonesia | 2016 | DENV3 |
| KT204456 | Indonesia | 2013 | DENV3 |
| LC436676 | Bangladesh | 2018 | DENV3 |
| MN922035 | China-Guangdong | 2020 | DENV3 |
| MN922034 | China-Guangdong | 2020 | DENV3 |
| LC436677 | Bangladesh | 2018 | DENV3 |
| LC436666 | Bangladesh | 2018 | DENV3 |
| LC436663 | Bangladesh | 2018 | DENV3 |
| LC436661 | Bangladesh | 2018 | DENV3 |
| KY495820 | Malaysia | 2017 | DENV3 |
| MG895255 | China-Taiwan | 2016 | DENV3 |
| KX224276 | Singapore | 2015 | DENV3 |
| MG778911 | China-Henan | 2018 | DENV3 |
| JN380808 | Singapore | 2010 | DENV3 |
| MG895276 | China-Taiwan | 2017 | DENV3 |
| MH036399 | Indonesia | 2017 | DENV3 |
| MH036400 | Indonesia | 2017 | DENV3 |
| MH036395 | Indonesia | 2017 | DENV3 |
| MG895206 | China-Taiwan | 2014 | DENV3 |
| MG895217 | China-Taiwan | 2015 | DENV3 |
| MK629477 | Indonesia | 2014 | DENV3 |
| KY709194 | Indonesia | 2016 | DENV3 |
| MG895215 | China-Taiwan | 2015 | DENV3 |
| MK629478 | Indonesia | 2015 | DENV3 |
| KY709188 | Indonesia | 2016 | DENV3 |
| MG895199 | China-Taiwan | 2014 | DENV3 |
| MG895208 | China-Taiwan | 2014 | DENV3 |
| KX646388 | Indonesia | 2010 | DENV3 |
| KX224295 | Singapore | 2014 | DENV3 |
| MH036408 | Indonesia | 2017 | DENV3 |
| MH173169 | Indonesia | 2017 | DENV3 |
| JF968113 | Indonesia | 2011 | DENV3 |
| JF968096 | Indonesia | 2011 | DENV3 |
| MH173172 | Indonesia | 2017 | DENV3 |
| MH178416 | Indonesia | 2017 | DENV3 |
| MH178417 | Indonesia | 2017 | DENV3 |
| MH173173 | Indonesia | 2017 | DENV3 |
| MN018382 | China-Guangdong | 2016 | DENV3 |
| KY006147 | Indonesia | 2016 | DENV3 |
| MH173167 | Indonesia | 2017 | DENV3 |
| MH173168 | Indonesia | 2017 | DENV3 |
| LC064747 | Indonesia | 2016 | DENV3 |
| MG895231 | China-Taiwan | 2015 | DENV3 |
| MG895237 | China-Taiwan | 2016 | DENV3 |
| KY006152 | Indonesia | 2016 | DENV3 |
| MG895262 | China-Taiwan | 2016 | DENV3 |
| KY006146 | Indonesia | 2016 | DENV3 |
| KT758794 | Indonesia | 2016 | DENV3 |
| MG895258 | China-Taiwan | 2016 | DENV3 |
| KY006149 | Indonesia | 2016 | DENV3 |
| MG895207 | China-Taiwan | 2014 | DENV3 |
| KT758793 | Indonesia | 2016 | DENV3 |
| MK629480 | Indonesia | 2016 | DENV3 |
| KY006144 | Indonesia | 2016 | DENV3 |
| MK629479 | Indonesia | 2015 | DENV3 |
| MK629481 | Indonesia | 2017 | DENV3 |
| KY006148 | Indonesia | 2016 | DENV3 |
| KP191531 | China-Yunnan | 2014 | DENV3 |
| MN018383 | China-Guangdong | 2015 | DENV3 |
| MG895239 | China-Taiwan | 2016 | DENV3 |
| MG895266 | China-Taiwan | 2016 | DENV3 |
| KX224284 | Singapore | 2015 | DENV3 |
| JF968082 | Indonesia | 2010 | DENV3 |
| JN575568 | Indonesia | 2009 | DENV3 |
| MN083245 | SriLanka | 2018 | DENV3 |
| EU448436 | Singapore | 2006 | DENV3 |
| KC762691 | Indonesia | 2008 | DENV3 |
| JF968074 | Indonesia | 2010 | DENV3 |
| JF968081 | Indonesia | 2010 | DENV3 |
| JN030192 | Singapore | 2011 | DENV3 |
| KM216738 | Indonesia | 2011 | DENV3 |
| KT204462 | Indonesia | 2011 | DENV3 |
| KT758758 | Indonesia | 2014 | DENV3 |
| MG895297 | China-Taiwan | 2013 | DENV3 |
| AY858037 | Indonesia | 2005 | DENV3 |
| JF968063 | Indonesia | 2009 | DENV3 |
| JF968087 | Indonesia | 2010 | DENV3 |
| AY858040 | Indonesia | 2005 | DENV3 |
| DQ518677 | Indonesia | 2004 | DENV3 |
| AY858043 | Indonesia | 2005 | DENV3 |
| AB189126 | Indonesia | 1999 | DENV3 |
| AB189127 | Indonesia | 1999 | DENV3 |
| AY858039 | Indonesia | 1999 | DENV3 |
| MG895261 | China-Taiwan | 2016 | DENV3 |
| MG895263 | China-Taiwan | 2016 | DENV3 |
| MG895270 | China-Taiwan | 2017 | DENV3 |
| MK629492 | Indonesia | 2016 | DENV3 |
| KY499643 | Indonesia | 2016 | DENV3 |
| MG895272 | China-Taiwan | 2017 | DENV3 |
| MG895216 | China-Taiwan | 2015 | DENV3 |
| MK629493 | Indonesia | 2016 | DENV3 |
| MG895275 | China-Taiwan | 2017 | DENV3 |
| MG895221 | China-Taiwan | 2015 | DENV3 |
| MH729991 | Indonesia | 2018 | DENV3 |
| MH036396 | Indonesia | 2017 | DENV3 |
| MH729989 | Indonesia | 2017 | DENV3 |
| MG895277 | China-Taiwan | 2017 | DENV3 |
| JF968070 | Indonesia | 2010 | DENV3 |
| AY858044 | Indonesia | 2005 | DENV3 |
| AY858045 | Indonesia | 2005 | DENV3 |
| AY858046 | Indonesia | 2005 | DENV3 |
| JF968071 | Indonesia | 2010 | DENV3 |
| MG895211 | China-Taiwan | 2014 | DENV3 |
| JF968057 | Indonesia | 2009 | DENV3 |
| JN568284 | Indonesia | 2010 | DENV3 |
| KX646386 | Indonesia | 2010 | DENV3 |
| AY858041 | Indonesia | 2005 | DENV3 |
| GQ357857 | Singapore | 2004 | DENV3 |
| GQ357849 | Singapore | 2006 | DENV3 |
| GQ357848 | Singapore | 2006 | DENV3 |
| GQ357845 | Singapore | 2006 | DENV3 |
| DQ518678 | Indonesia | 2006 | DENV3 |
| KC762692 | Indonesia | 2010 | DENV3 |
| KC762693 | Indonesia | 2010 | DENV3 |
| EU448437 | Indonesia | 2004 | DENV3 |
| MG895194 | China-Taiwan | 2013 | DENV3 |
| AB214881 | East Timor | 2006 | DENV3 |
| DQ453976 | East Timor | 2006 | DENV3 |
| AB214882 | East Timor | 2006 | DENV3 |
| DQ453970 | East Timor | 2006 | DENV3 |
| DQ453972 | East Timor | 2006 | DENV3 |
| DQ453978 | East Timor | 2006 | DENV3 |
| DQ453971 | East Timor | 2006 | DENV3 |
| DQ453975 | East Timor | 2006 | DENV3 |
| DQ453981 | East Timor | 2006 | DENV3 |
| DQ453973 | East Timor | 2006 | DENV3 |
| DQ453979 | East Timor | 2006 | DENV3 |
| AB214879 | East Timor | 2006 | DENV3 |
| DQ453969 | East Timor | 2006 | DENV3 |
| DQ453974 | East Timor | 2006 | DENV3 |
| DQ453980 | East Timor | 2006 | DENV3 |
| KY072809 | East Timor | 2002 | DENV3 |
| KY275235 | East Timor | 2002 | DENV3 |
| KY275243 | East Timor | 2002 | DENV3 |
| JN575566 | East Timor | 2001 | DENV3 |
| AB189128 | Indonesia | 1999 | DENV3 |
| FM986662 | Malaysia | 1998 | DENV3 |
| FM986663 | Malaysia | 1998 | DENV3 |
| DQ518675 | China-Taiwan | 2000 | DENV3 |
| AY858038 | Indonesia | 1989 | DENV3 |
| DQ518674 | Indonesia | 1992 | DENV3 |
| KC762681 | Indonesia | 2008 | DENV3 |
| KC762683 | Indonesia | 2008 | DENV3 |
| KC762682 | Indonesia | 2008 | DENV3 |
| KC762688 | Indonesia | 2008 | DENV3 |
| KC762685 | Indonesia | 2008 | DENV3 |
| KC762690 | Indonesia | 2009 | DENV3 |
| KC762686 | Indonesia | 2008 | DENV3 |
| KC762687 | Indonesia | 2008 | DENV3 |
| KC762689 | Indonesia | 2008 | DENV3 |
| MH729987 | Philippines | 2016 | DENV3 |
| MH729990 | Philippines | 2017 | DENV3 |
| MN018379 | China-Guangdong | 2016 | DENV3 |
| MN922037 | China-Guangdong | 2020 | DENV3 |
| MN922038 | China-Guangdong | 2020 | DENV3 |
| MN018389 | China-Guangdong | 2017 | DENV3 |
| KX224287 | Singapore | 2015 | DENV3 |
| MN018380 | China-Guangdong | 2016 | DENV3 |
| DQ675519 | China-Taiwan | 1996 | DENV3 |
| KT452798 | Indonesia | 1979 | DENV3 |
| EU448464 | Singapore | 2002 | DENV4 |
| FM986674 | Malaysia | 2003 | DENV4 |
| MK238000 | Vietnam | 2017 | DENV4 |
| MK238034 | Vietnam | 2016 | DENV4 |
| MK238033 | Vietnam | 2016 | DENV4 |
| MK238009 | Vietnam | 2017 | DENV4 |
| MK238012 | Vietnam | 2017 | DENV4 |
| MK238017 | Vietnam | 2017 | DENV4 |
| MK238020 | Vietnam | 2017 | DENV4 |
| MK238023 | Vietnam | 2016 | DENV4 |
| MK238026 | Vietnam | 2016 | DENV4 |
| MK238008 | Vietnam | 2017 | DENV4 |
| MK238003 | Vietnam | 2017 | DENV4 |
| MK238015 | Vietnam | 2017 | DENV4 |
| MK238019 | Vietnam | 2018 | DENV4 |
| MK238021 | Vietnam | 2016 | DENV4 |
| MK238018 | Vietnam | 2018 | DENV4 |
| MK238022 | Vietnam | 2016 | DENV4 |
| MK238030 | Vietnam | 2016 | DENV4 |
| MK238001 | Vietnam | 2017 | DENV4 |
| MK238002 | Vietnam | 2017 | DENV4 |
| MK238004 | Vietnam | 2017 | DENV4 |
| MK238028 | Vietnam | 2016 | DENV4 |
| MK238032 | Vietnam | 2016 | DENV4 |
| MG895359 | China-Taiwan | 2015 | DENV4 |
| MK238014 | Vietnam | 2017 | DENV4 |
| MK238006 | Vietnam | 2017 | DENV4 |
| MN018394 | China-Guangdong | 2016 | DENV4 |
| MK238024 | Vietnam | 2016 | DENV4 |
| MG895373 | China-Taiwan | 2016 | DENV4 |
| MG895378 | China-Taiwan | 2016 | DENV4 |
| MG895354 | China-Taiwan | 2015 | DENV4 |
| KP792537 | Singapore | 2012 | DENV4 |
| MG895342 | China-Taiwan | 2014 | DENV4 |
| MG895325 | China-Taiwan | 2013 | DENV4 |
| MG895320 | China-Taiwan | 2013 | DENV4 |
| MK614090 | China-Guangdong | 2019 | DENV4 |
| MK614092 | China-Guangdong | 2018 | DENV4 |
| MK614091 | China-Guangdong | 2019 | DENV4 |
| MK238013 | Vietnam | 2017 | DENV4 |
| MK238029 | Vietnam | 2016 | DENV4 |
| MK614093 | China-Guangdong | 2018 | DENV4 |
| MG895368 | China-Taiwan | 2016 | DENV4 |
| MG895375 | China-Taiwan | 2016 | DENV4 |
| MG895394 | China-Taiwan | 2017 | DENV4 |
| MG895337 | China-Taiwan | 2014 | DENV4 |
| MG895323 | China-Taiwan | 2013 | DENV4 |
| KF955521 | Thailand | 2007 | DENV4 |
| KY586942 | Thailand | 2007 | DENV4 |
| EU448453 | Cambodia | 2004 | DENV4 |
| AB111089 | Thailand | 2003 | DENV4 |
| KY586941 | Thailand | 1999 | DENV4 |
| EU478410 | Myanmar | 2007 | DENV4 |
| AY618964 | Thailand | 1992 | DENV4 |
| AY618966 | Thailand | 1992 | DENV4 |
| AY618972 | Thailand | 1995 | DENV4 |
| MN027549 | Philippines | 2016 | DENV4 |
| AY618944 | Thailand | 2002 | DENV4 |
| KY586914 | Thailand | 2001 | DENV4 |
| KY586913 | Thailand | 2002 | DENV4 |
| KY586920 | Thailand | 2002 | DENV4 |
| AY618936 | Thailand | 2001 | DENV4 |
| KY586921 | Thailand | 2002 | DENV4 |
| KY586917 | Thailand | 2001 | DENV4 |
| KY586919 | Thailand | 2001 | DENV4 |
| MG895388 | China-Taiwan | 2017 | DENV4 |
| MG895389 | China-Taiwan | 2017 | DENV4 |
| MG895347 | China-Taiwan | 2014 | DENV4 |
| MG895338 | China-Taiwan | 2014 | DENV4 |
| MG895313 | China-Taiwan | 2012 | DENV4 |
| KT749997 | Philippines | 2012 | DENV4 |
| MG895348 | China-Taiwan | 2014 | DENV4 |
| MN018393 | China-Guangdong | 2016 | DENV4 |
| JQ922560 | India | 2010 | DENV4 |
| MN083244 | Sri Lanka | 2018 | DENV4 |
| JQ993252 | Thailand | 2008 | DENV4 |
| MG895298 | China-Taiwan | 2011 | DENV4 |
| JF967767 | Indonesia | 2009 | DENV4 |
| JF967779 | Indonesia | 2011 | DENV4 |
| MH174974 | India | 2018 | DENV4 |
| MK654681 | India | 2018 | DENV4 |
| MH174973 | India | 2018 | DENV4 |
| MH891769 | India | 2018 | DENV4 |
| MN239487 | India | 2019 | DENV4 |
| MN244932 | India | 2018 | DENV4 |
| MN244933 | India | 2018 | DENV4 |
| MN244929 | India | 2018 | DENV4 |
| MK858144 | India | 2017 | DENV4 |
| MK858142 | India | 2017 | DENV4 |
| MN239489 | India | 2017 | DENV4 |
| MH594955 | India | 2016 | DENV4 |
| MH594957 | India | 2016 | DENV4 |
| MG272272 | India | 2017 | DENV4 |
| MG272274 | India | 2017 | DENV4 |
| MN244927 | India | 2018 | DENV4 |
| MG272273 | India | 2017 | DENV4 |
| MH594953 | India | 2018 | DENV4 |
| MH594954 | India | 2018 | DENV4 |
| MH174975 | India | 2018 | DENV4 |
| MH010604 | China-Zhejiang | 2018 | DENV4 |
| AY618984 | Thailand | 2000 | DENV4 |
| KY586832 | Thailand | 2000 | DENV4 |
| KY586924 | Thailand | 2004 | DENV4 |
| KY586863 | Thailand | 2007 | DENV4 |
| KY586911 | Thailand | 2002 | DENV4 |
| EU448463 | Indonesia | 2008 | DENV4 |
| JF967769 | Indonesia | 2010 | DENV4 |
| KX812530 | Indonesia | 1977 | DENV4 |
| MK780893 | Thailand | 2016 | DENV4 |
| JF967773 | Pakistan | 2010 | DENV4 |
| KF041260 | Pakistan | 2010 | DENV4 |
| MH173175 | Indonesia | 2017 | DENV4 |
| MT377742 | Indonesia | 2020 | DENV4 |
| MG895376 | China-Taiwan | 2016 | DENV4 |
| MT377736 | Indonesia | 2019 | DENV4 |
| MT377746 | Indonesia | 2020 | DENV4 |
| AY618946 | Thailand | 2003 | DENV4 |
| KY586930 | Thailand | 2003 | DENV4 |
| AY618939 | Thailand | 2001 | DENV4 |
| KY586925 | Thailand | 2003 | DENV4 |
| KY586929 | Thailand | 2003 | DENV4 |
| KY586926 | Thailand | 2003 | DENV4 |
| KY586927 | Thailand | 2002 | DENV4 |
| KY586928 | Thailand | 2002 | DENV4 |
| KY586931 | Thailand | 2003 | DENV4 |
| KY586932 | Thailand | 2001 | DENV4 |
| KY586934 | Thailand | 2002 | DENV4 |
| AY618943 | Thailand | 2002 | DENV4 |
| KY586937 | Thailand | 2001 | DENV4 |
| KY586936 | Thailand | 2001 | DENV4 |
| KY586935 | Thailand | 2001 | DENV4 |
| KP100258 | Thailand | 2013 | DENV4 |
| KY586923 | Thailand | 2004 | DENV4 |
| JQ993254 | Thailand | 2007 | DENV4 |
| KY921908 | Singapore | 2015 | DENV4 |
| MK238031 | Vietnam | 2016 | DENV4 |
| MG895356 | China-Taiwan | 2015 | DENV4 |
| LC064748 | Malaysia | 2015 | DENV4 |
| KR779789 | Singapore | 2014 | DENV4 |
| KX224304 | Singapore | 2014 | DENV4 |
| KF543272 | Cambodia | 2011 | DENV4 |
| MT377740 | Indonesia | 2019 | DENV4 |
| MW301595 | China-Guangdong | 2021 | DENV4 |
| MT377749 | Indonesia | 2020 | DENV4 |
| KU529755 | Indonesia | 2015 | DENV4 |
| KU529756 | Indonesia | 2015 | DENV4 |
| EU448459 | Indonesia | 2008 | DENV4 |
| JF967775 | Indonesia | 2010 | DENV4 |
| JF967772 | Indonesia | 2010 | DENV4 |
| KX224298 | Singapore | 2014 | DENV4 |
| JF967783 | Indonesia | 2011 | DENV4 |
| KX646389 | Indonesia | 2010 | DENV4 |
| EU448455 | Cambodia | 2006 | DENV4 |
| JF967761 | Indonesia | 2009 | DENV4 |
| KX845005 | India | 2016 | DENV4 |
| KT452802 | Cambodia | 2012 | DENV4 |
| JN019829 | Singapore | 2010 | DENV4 |
| KY586860 | Thailand | 2007 | DENV4 |
| MN018392 | China-Guangdong | 2016 | DENV4 |
| MN018395 | China-Guangdong | 2016 | DENV4 |
| AB873105 | Cambodia | 2014 | DENV4 |
| JQ993262 | Thailand | 2007 | DENV4 |
| KY586862 | Thailand | 2005 | DENV4 |
| KY586861 | Thailand | 2005 | DENV4 |
| AY618945 | Thailand | 2003 | DENV4 |
| KY586905 | Thailand | 2005 | DENV4 |
| KY586906 | Thailand | 2003 | DENV4 |
| KY586898 | Thailand | 2001 | DENV4 |
| KM216740 | Indonesia | 2010 | DENV4 |
| MG895339 | China-Taiwan | 2014 | DENV4 |
| JF967774 | Indonesia | 2010 | DENV4 |
| MG933856 | China-Yunnan | 2018 | DENV4 |
| MG933866 | China-Yunnan | 2018 | DENV4 |
| LC410203 | Thailand | 2018 | DENV4 |
| KU509296 | Thailand | 2014 | DENV4 |
| JQ993287 | Thailand | 2005 | DENV4 |
| JQ993300 | Thailand | 2005 | DENV4 |
| JQ993285 | Thailand | 2005 | DENV4 |
| KY586852 | Thailand | 2007 | DENV4 |
| KY586853 | Thailand | 2007 | DENV4 |
| JQ993297 | Thailand | 2005 | DENV4 |
| KY586854 | Thailand | 2005 | DENV4 |
| KY586851 | Thailand | 2005 | DENV4 |
| JQ993299 | Thailand | 2005 | DENV4 |
| KP100259 | Thailand | 2013 | DENV4 |
| KY586849 | Thailand | 2007 | DENV4 |
| KY586847 | Thailand | 2007 | DENV4 |
| KY586848 | Thailand | 2005 | DENV4 |
| KY586850 | Thailand | 2007 | DENV4 |
| KY586842 | Thailand | 2007 | DENV4 |
| KY586845 | Thailand | 2007 | DENV4 |
| KY586846 | Thailand | 2007 | DENV4 |
| KY586840 | Thailand | 2011 | DENV4 |
| KY586841 | Thailand | 2011 | DENV4 |
| EU448454 | Thailand | 2008 | DENV4 |
| JN575594 | Thailand | 2011 | DENV4 |
| KY586858 | Thailand | 2008 | DENV4 |
| KY586859 | Thailand | 2007 | DENV4 |
| KT026310 | Thailand | 2012 | DENV4 |
| KY586857 | Thailand | 2004 | DENV4 |
| KY586855 | Thailand | 2006 | DENV4 |
| KY586856 | Thailand | 2005 | DENV4 |
| KY586864 | Thailand | 2005 | DENV4 |
| EU448456 | Thailand | 2006 | DENV4 |
| KP792536 | Singapore | 2005 | DENV4 |
| KF955531 | Thailand | 2007 | DENV4 |
| KY586867 | Thailand | 2005 | DENV4 |
| KY586865 | Thailand | 2006 | DENV4 |
| KY586866 | Thailand | 2005 | DENV4 |
| KY586868 | Thailand | 2006 | DENV4 |
| JQ993295 | Thailand | 2005 | DENV4 |
| KJ470764 | Myanmar | 2014 | DENV4 |
| KY586922 | Thailand | 1999 | DENV4 |
| KX262921 | China-Yunnan | 2016 | DENV4 |
| KY672956 | China-Yunnan | 2016 | DENV4 |
| KX262922 | China-Yunnan | 2016 | DENV4 |
| KX262926 | China-Yunnan | 2016 | DENV4 |
| MG895366 | China-Taiwan | 2016 | DENV4 |
| MH893692 | Myanmar | 2016 | DENV4 |
| MH893691 | China-Yunnan | 2016 | DENV4 |
| KR051896 | Myanmar | 2014 | DENV4 |
| KY586886 | Thailand | 2002 | DENV4 |
| KY586889 | Thailand | 2004 | DENV4 |
| KY586891 | Thailand | 2006 | DENV4 |
| KY586888 | Thailand | 2005 | DENV4 |
| KY586892 | Thailand | 2003 | DENV4 |
| KY586894 | Thailand | 2003 | DENV4 |
| KY586895 | Thailand | 2003 | DENV4 |
| KY586896 | Thailand | 2003 | DENV4 |
| KY586869 | Thailand | 2002 | DENV4 |
| JQ993237 | Thailand | 2007 | DENV4 |
| JQ993263 | Thailand | 2007 | DENV4 |
| JQ993277 | Thailand | 2006 | DENV4 |
| KY586883 | Thailand | 2007 | DENV4 |
| KY586882 | Thailand | 2005 | DENV4 |
| KY586885 | Thailand | 2005 | DENV4 |
| KY586879 | Thailand | 2005 | DENV4 |
| KY586880 | Thailand | 2006 | DENV4 |
| KY586881 | Thailand | 2005 | DENV4 |
| JQ993233 | Thailand | 2007 | DENV4 |
| KF955522 | Thailand | 2007 | DENV4 |
| MK450487 | Thailand | 2006 | DENV4 |
| JQ993250 | Thailand | 2005 | DENV4 |
| JQ993286 | Thailand | 2005 | DENV4 |
| JQ993304 | Thailand | 2005 | DENV4 |
| JQ993251 | Thailand | 2008 | DENV4 |
| KY586874 | Thailand | 2008 | DENV4 |
| KY586875 | Thailand | 2007 | DENV4 |
| KY586876 | Thailand | 2005 | DENV4 |
| KY586877 | Thailand | 2004 | DENV4 |
| KY586870 | Thailand | 2003 | DENV4 |
| KY586871 | Thailand | 2009 | DENV4 |
| KY586872 | Thailand | 2005 | DENV4 |
| KY586873 | Thailand | 2007 | DENV4 |
| KY586878 | Thailand | 2003 | DENV4 |
| KR922405 | Thailand | 2012 | DENV4 |
| KY586897 | Thailand | 2003 | DENV4 |
| KY586912 | Thailand | 2001 | DENV4 |
| AY618938 | Thailand | 2001 | DENV4 |
| KY586907 | Thailand | 2004 | DENV4 |
| KY586910 | Thailand | 2002 | DENV4 |
| KY586909 | Thailand | 2001 | DENV4 |
| KY586903 | Thailand | 2001 | DENV4 |
| KY586902 | Thailand | 2001 | DENV4 |
| AY618947 | Thailand | 2003 | DENV4 |
| AY618992 | Thailand | 2002 | DENV4 |
| MK450486 | Thailand | 2006 | DENV4 |
| KY586899 | Thailand | 2003 | DENV4 |
| AY618935 | Thailand | 2001 | DENV4 |
| KY586900 | Thailand | 2002 | DENV4 |
| KY586901 | Thailand | 2003 | DENV4 |
| AY618987 | Thailand | 2000 | DENV4 |
| AY618985 | Thailand | 2000 | DENV4 |
| JQ993302 | Thailand | 2005 | DENV4 |
| AY618969 | Thailand | 1994 | DENV4 |
| KY586939 | Thailand | 1996 | DENV4 |
| AY618977 | Thailand | 1997 | DENV4 |
| KY586940 | Thailand | 1996 | DENV4 |
| KY586938 | Thailand | 1996 | DENV4 |
| AY618968 | Thailand | 1993 | DENV4 |
| AY618973 | Thailand | 1995 | DENV4 |
| MG895363 | China-Taiwan | 2016 | DENV4 |
| KT750005 | Philippines | 2014 | DENV4 |
| KJ938506 | India | 2012 | DENV4 |
| KT239366 | Pakistan | 2015 | DENV4 |
| KJ938501 | India | 2012 | DENV4 |
| KJ938507 | India | 2012 | DENV4 |
| KT239364 | Pakistan | 2015 | DENV4 |
| KJ938505 | India | 2012 | DENV4 |
| KJ938503 | India | 2012 | DENV4 |
| KJ938504 | India | 2012 | DENV4 |
| KJ938502 | India | 2012 | DENV4 |
| MN018398 | China-Guangdong | 2017 | DENV4 |
| KY978446 | India | 2013 | DENV4 |
| MK858145 | India | 2017 | DENV4 |
| JN940921 | India | 2010 | DENV4 |
| HM237348 | India | 2008 | DENV4 |
| MN244930 | India | 2018 | DENV4 |
| MH594950 | India | 2015 | DENV4 |
| MH594951 | India | 2015 | DENV4 |
| MT182025 | India | 2018 | DENV4 |
| MH594952 | India | 2016 | DENV4 |
| MH594958 | India | 2016 | DENV4 |
| KU509287 | India | 2010 | DENV4 |
| MG895383 | China-Taiwan | 2016 | DENV4 |
| MN018390 | China-Guangdong | 2014 | DENV4 |
| MK858146 | India | 2017 | DENV4 |
| MT182024 | India | 2018 | DENV4 |
| JF967790 | India | 2011 | DENV4 |
| KY978447 | India | 2013 | DENV4 |
| HM237349 | India | 2008 | DENV4 |
| KX262924 | China-Yunnan | 2016 | DENV4 |
| KT749994 | Thailand | 2012 | DENV4 |
| KY586827 | Thailand | 2001 | DENV4 |
| KC762695 | Indonesia | 2008 | DENV4 |
| KC762697 | Indonesia | 2008 | DENV4 |
| MG933854 | China-Yunnan | 2018 | DENV4 |
| KR051892 | Myanmar | 2014 | DENV4 |
| KR051893 | Myanmar | 2014 | DENV4 |
| KX224310 | Singapore | 2014 | DENV4 |
| JN019828 | Singapore | 2011 | DENV4 |
| MG895345 | China-Taiwan | 2014 | DENV4 |
| KX224299 | Singapore | 2014 | DENV4 |
| KY427077 | Indonesia | 2017 | DENV4 |
| MG895392 | China-Taiwan | 2017 | DENV4 |
| MG895393 | China-Taiwan | 2017 | DENV4 |
| MN018396 | China-Guangdong | 2016 | DENV4 |
| MG895365 | China-Taiwan | 2016 | DENV4 |
| MG895353 | China-Taiwan | 2015 | DENV4 |
| MG933862 | China-Yunnan | 2018 | DENV4 |
| MN955689 | Thailand | 2018 | DENV4 |
| MG895352 | China-Taiwan | 2015 | DENV4 |
| MG895381 | China-Taiwan | 2017 | DENV4 |
| MG895360 | China-Taiwan | 2016 | DENV4 |
| MN027554 | Philippines | 2016 | DENV4 |
| MN027556 | Philippines | 2016 | DENV4 |
| MG895355 | China-Taiwan | 2015 | DENV4 |
| MG895374 | China-Taiwan | 2016 | DENV4 |
| MK640208 | China-Guangdong | 2019 | DENV4 |
| MH173174 | Indonesia | 2017 | DENV4 |
| MN027555 | Philippines | 2016 | DENV4 |
| MN027552 | Philippines | 2016 | DENV4 |
| MN027548 | Philippines | 2016 | DENV4 |
| MN027559 | Philippines | 2017 | DENV4 |
| MN027558 | Philippines | 2016 | DENV4 |
| KX224309 | Singapore | 2015 | DENV4 |
| JN544414 | Singapore | 2011 | DENV4 |
| MG895344 | China-Taiwan | 2014 | DENV4 |
| JN575590 | Malaysia | 2010 | DENV4 |
| MG895315 | China-Taiwan | 2013 | DENV4 |
| MG895308 | China-Taiwan | 2012 | DENV4 |
| KX224306 | Singapore | 2014 | DENV4 |
| KY921910 | Singapore | 2016 | DENV4 |
| KU523872 | Indonesia | 2015 | DENV4 |
| MG895387 | China-Taiwan | 2017 | DENV4 |
| KR779790 | Singapore | 2014 | DENV4 |
| JN575588 | Indonesia | 2011 | DENV4 |
| JN544415 | Singapore | 2011 | DENV4 |
| KM216739 | Indonesia | 2011 | DENV4 |
| KX224300 | Singapore | 2014 | DENV4 |
| JF967781 | Indonesia | 2011 | DENV4 |
| KM216744 | Indonesia | 2011 | DENV4 |
| MG895331 | China-Taiwan | 2014 | DENV4 |
| MN018397 | China-Guangdong | 2016 | DENV4 |
| KY586837 | Thailand | 1996 | DENV4 |
| KY427081 | Indonesia | 2017 | DENV4 |
| MK629501 | Indonesia | 2015 | DENV4 |
| MG895372 | China-Taiwan | 2016 | DENV4 |
| LC410201 | Thailand | 2017 | DENV4 |
| MK780892 | Thailand | 2016 | DENV4 |
| MG564133 | Thailand | 2016 | DENV4 |
| MN955687 | Thailand | 2018 | DENV4 |
| LC410200 | Thailand | 2017 | DENV4 |
| MG895369 | China-Taiwan | 2016 | DENV4 |
| MG564136 | Thailand | 2016 | DENV4 |
| KT825074 | Thailand | 2016 | DENV4 |
| MG895384 | China-Taiwan | 2017 | DENV4 |
| MK780897 | Thailand | 2017 | DENV4 |
| KX357899 | Myanmar | 2016 | DENV4 |
| MN955690 | Thailand | 2019 | DENV4 |
| MN955691 | Thailand | 2019 | DENV4 |
| MH893694 | Thailand | 2016 | DENV4 |
| MK780895 | Thailand | 2017 | DENV4 |
| MK780896 | Thailand | 2017 | DENV4 |
| MK780894 | Thailand | 2017 | DENV4 |
| MG564132 | Thailand | 2016 | DENV4 |
| MG564131 | Thailand | 2016 | DENV4 |
| KU509300 | Thailand | 2014 | DENV4 |
| KX224312 | Singapore | 2015 | DENV4 |
| KJ470765 | Myanmar | 2014 | DENV4 |
| MG933852 | China-Yunnan | 2018 | DENV4 |
| MG933853 | China-Yunnan | 2018 | DENV4 |
| MG933855 | China-Yunnan | 2018 | DENV4 |
| MG933858 | China-Yunnan | 2018 | DENV4 |
| MT835138 | China-Guangdong | 2017 | DENV4 |
| MG933851 | China-Yunnan | 2018 | DENV4 |
| MG933865 | China-Yunnan | 2018 | DENV4 |
| MK614088 | China-Guangdong | 2019 | DENV4 |
| MG933859 | China-Yunnan | 2018 | DENV4 |
| MG933861 | China-Yunnan | 2018 | DENV4 |
| MG933860 | China-Yunnan | 2018 | DENV4 |
| MG933857 | China-Yunnan | 2018 | DENV4 |
| MN955693 | Thailand | 2019 | DENV4 |
| MW295825 | China-Guangdong | 2018 | DENV4 |
| KR051897 | Myanmar | 2014 | DENV4 |
| MG895351 | China-Taiwan | 2015 | DENV4 |
| MG895333 | China-Taiwan | 2014 | DENV4 |
| KT750007 | Thailand | 2014 | DENV4 |
| MH893695 | Myanmar | 2017 | DENV4 |
| MH893699 | China-Yunnan | 2017 | DENV4 |
| KX262923 | China-Yunnan | 2016 | DENV4 |
| KX262925 | China-Yunnan | 2016 | DENV4 |
| KX262920 | China-Yunnan | 2016 | DENV4 |
| KR051894 | Myanmar | 2014 | DENV4 |
| MG895364 | China-Taiwan | 2016 | DENV4 |
| MH893693 | Myanmar | 2016 | DENV4 |
| MH893697 | China-Yunnan | 2017 | DENV4 |
| MH893698 | China-Yunnan | 2017 | DENV4 |
| MG895395 | China-Taiwan | 2017 | DENV4 |
| MG933864 | China-Yunnan | 2018 | DENV4 |
| MG933867 | China-Yunnan | 2018 | DENV4 |
| KX357897 | Myanmar | 2016 | DENV4 |
| KT749998 | Thailand | 2012 | DENV4 |
| MG564134 | Thailand | 2016 | DENV4 |
| MG895367 | China-Taiwan | 2016 | DENV4 |
| MG564138 | Thailand | 2016 | DENV4 |
| MG895391 | China-Taiwan | 2017 | DENV4 |
| LC410198 | Thailand | 2017 | DENV4 |
| MH893690 | China-Yunnan | 2014 | DENV4 |
| MG895332 | China-Taiwan | 2014 | DENV4 |
| MN955688 | Thailand | 2018 | DENV4 |
| LC410197 | Thailand | 2017 | DENV4 |
| KY451945 | Thailand | 2014 | DENV4 |
| MG895385 | China-Taiwan | 2017 | DENV4 |
| MG895390 | China-Taiwan | 2017 | DENV4 |
| MG895386 | China-Taiwan | 2017 | DENV4 |
| MN018391 | China-Guangdong | 2016 | DENV4 |
| KX357895 | Myanmar | 2016 | DENV4 |
| KX357896 | Myanmar | 2016 | DENV4 |
| MG895362 | China-Taiwan | 2016 | DENV4 |
| KT452793 | Myanmar | 2009 | DENV4 |
| JF967792 | Myanmar | 2011 | DENV4 |
| MG895300 | China-Taiwan | 2012 | DENV4 |
| KY586829 | Thailand | 2011 | DENV4 |
| KY586828 | Thailand | 2007 | DENV4 |
| MG895303 | China-Taiwan | 2012 | DENV4 |
| MG895350 | China-Taiwan | 2015 | DENV4 |
| MG564137 | Thailand | 2016 | DENV4 |
| KY586830 | Thailand | 2003 | DENV4 |
| KY586831 | Thailand | 2004 | DENV4 |
| AY618980 | Thailand | 1999 | DENV4 |
| KY586834 | Thailand | 1999 | DENV4 |
| KY586835 | Thailand | 1999 | DENV4 |
| KY038921 | China-Yunnan | 2015 | DENV4 |
| KY586836 | Thailand | 1996 | DENV4 |
| AY618971 | Thailand | 1994 | DENV4 |
| KY586838 | Thailand | 1996 | DENV4 |
| AY618976 | Thailand | 1997 | DENV4 |
| KY586839 | Thailand | 1996 | DENV4 |
| MT377743 | Indonesia | 2020 | DENV4 |
| MT377747 | Indonesia | 2020 | DENV4 |
| MG895377 | China-Taiwan | 2016 | DENV4 |
| KF052668 | Indonesia | 2013 | DENV4 |
| MK629497 | Indonesia | 2016 | DENV4 |
| MT377745 | Indonesia | 2020 | DENV4 |
| KY006156 | Indonesia | 2016 | DENV4 |
| MG895358 | China-Taiwan | 2015 | DENV4 |
| MK629502 | Indonesia | 2016 | DENV4 |
| MG895382 | China-Taiwan | 2017 | DENV4 |
| MK629495 | Indonesia | 2015 | DENV4 |
| MK629496 | Indonesia | 2015 | DENV4 |
| JF967768 | Indonesia | 2010 | DENV4 |
| MK629494 | Indonesia | 2014 | DENV4 |
| MG895312 | China-Taiwan | 2012 | DENV4 |
| MH178418 | Indonesia | 2017 | DENV4 |
| KT204464 | Indonesia | 2011 | DENV4 |
| MH178419 | Indonesia | 2017 | DENV4 |
| KU529758 | Indonesia | 2016 | DENV4 |
| MK629498 | Indonesia | 2016 | DENV4 |
| MG895309 | China-Taiwan | 2012 | DENV4 |
| MG895310 | China-Taiwan | 2012 | DENV4 |
| JF967784 | Indonesia | 2011 | DENV4 |
| KX224308 | Singapore | 2015 | DENV4 |
| JF967771 | Indonesia | 2010 | DENV4 |
| KY709196 | Indonesia | 2016 | DENV4 |
| MK629500 | Indonesia | 2016 | DENV4 |
| JQ403526 | China-Taiwan | 2011 | DENV4 |
| JF967766 | Indonesia | 2009 | DENV4 |
| MG895357 | China-Taiwan | 2015 | DENV4 |
| JF967764 | Indonesia | 2009 | DENV4 |
| KF052666 | Indonesia | 2013 | DENV4 |
| MG895349 | China-Taiwan | 2014 | DENV4 |
| KU529757 | Indonesia | 2016 | DENV4 |
| MG895379 | China-Taiwan | 2016 | DENV4 |
| KY709195 | Indonesia | 2016 | DENV4 |
| MG895311 | China-Taiwan | 2012 | DENV4 |
| MG895346 | China-Taiwan | 2014 | DENV4 |
| KX224307 | Singapore | 2014 | DENV4 |
| JN575589 | Indonesia | 2011 | DENV4 |
| MG895301 | China-Taiwan | 2012 | DENV4 |
| JN544416 | Singapore | 2011 | DENV4 |
| MG895302 | China-Taiwan | 2012 | DENV4 |
| MG895299 | China-Taiwan | 2011 | DENV4 |
| JF967789 | Indonesia | 2011 | DENV4 |
| KF052667 | Indonesia | 2013 | DENV4 |
| MG895343 | China-Taiwan | 2014 | DENV4 |
| KX646390 | Indonesia | 2010 | DENV4 |
| GQ398256 | Singapore | 2006 | DENV4 |
| MK780900 | Thailand | 2016 | DENV4 |
| MK780898 | Thailand | 2016 | DENV4 |
| MK780901 | Thailand | 2016 | DENV4 |
| EU448458 | Philippines | 2005 | DENV4 |
| KC762696 | Indonesia | 2008 | DENV4 |
| KJ545440 | China-Guangdong | 2011 | DENV4 |
| JF967759 | Indonesia | 2009 | DENV4 |
| AY618955 | Thailand | 1983 | DENV4 |
| AY618959 | Thailand | 1985 | DENV4 |
| KY670635 | China-Taiwan | 2004 | DENV4 |
| MG895304 | China-Taiwan | 2012 | DENV4 |
| MG895328 | China-Taiwan | 2013 | DENV4 |
| MG895329 | China-Taiwan | 2013 | DENV4 |
| KU523871 | Philippines | 2014 | DENV4 |
| JN019816 | Singapore | 2011 | DENV4 |
| JN019823 | Singapore | 2011 | DENV4 |
| JF967778 | Indonesia | 2010 | DENV4 |
| JF967777 | Indonesia | 2010 | DENV4 |
| AY858049 | Indonesia | 2005 | DENV4 |
| JN544417 | Singapore | 2011 | DENV4 |
| MK629499 | Indonesia | 2014 | DENV4 |
| AY618953 | Thailand | 1982 | DENV4 |
| AY618962 | Thailand | 1987 | DENV4 |
| AY618960 | Thailand | 1986 | DENV4 |
| AY618961 | Thailand | 1986 | DENV4 |
| KY586943 | Thailand | 1996 | DENV4 |
| JF967776 | Philippines | 2010 | DENV4 |
| KT750001 | Philippines | 2013 | DENV4 |
| MG895334 | China-Taiwan | 2014 | DENV4 |
| KR011349 | Philippines | 1957 | DENV4 |
| AY618952 | Thailand | 1981 | DENV4 |
| KY709136 | Philippines | 2011 | DENV4 |
| MG895327 | China-Taiwan | 2013 | DENV4 |
| MG895326 | China-Taiwan | 2013 | DENV4 |
| AY858050 | Indonesia | 2005 | DENV4 |
| MG895341 | China-Taiwan | 2014 | DENV4 |
| KT750006 | Philippines | 2014 | DENV4 |
| MN027553 | Philippines | 2016 | DENV4 |
| KU509297 | Philippines | 2016 | DENV4 |
| KU509299 | Thailand | 2014 | DENV4 |
| MG895322 | China-Taiwan | 2013 | DENV4 |
| JF967763 | Indonesia | 2009 | DENV4 |
| JN575583 | Indonesia | 2011 | DENV4 |
| EU448461 | Indonesia | 2005 | DENV4 |
| MG895319 | China-Taiwan | 2013 | DENV4 |
| MN027546 | Philippines | 2016 | DENV4 |
| KC333651 | China-Guangdong | 2013 | DENV4 |
| MN027557 | Philippines | 2016 | DENV4 |
| MG895370 | China-Taiwan | 2016 | DENV4 |
| MN027551 | Philippines | 2016 | DENV4 |
| MG895380 | China-Taiwan | 2017 | DENV4 |
| KX224303 | Singapore | 2014 | DENV4 |
| MG895396 | China-Taiwan | 2013 | DENV4 |
| MG895321 | China-Taiwan | 2013 | DENV4 |
| JF967786 | Philippines | 2011 | DENV4 |
| KX147336 | Thailand | 2015 | DENV4 |
| MG895336 | China-Taiwan | 2014 | DENV4 |
| MG895318 | China-Taiwan | 2013 | DENV4 |
| KT749999 | Philippines | 2013 | DENV4 |
| MG895306 | China-Taiwan | 2012 | DENV4 |
| JN022608 | Indonesia | 1979 | DENV4 |
| KT452801 | Indonesia | 1974 | DENV4 |
| KY586825 | Thailand | 2002 | DENV4 |
| KY586826 | Thailand | 2002 | DENV4 |
| KY586824 | Thailand | 2002 | DENV4 |
| KY586823 | Thailand | 2003 | DENV4 |
| MG895330 | China-Taiwan | 2014 | DENV4 |
| FM986664 | Malaysia | 2000 | DENV4 |
| FM986672 | Malaysia | 1998 | DENV4 |
| KT239367 | Pakistan | 2015 | DENV4 |
| AY618954 | Thailand | 1983 | DENV4 |
| AY618967 | Thailand | 1993 | DENV4 |
| AY618991 | Thailand | 1978 | DENV4 |
| AY618965 | Thailand | 1992 | DENV4 |
| AY618970 | Thailand | 1994 | DENV4 |
| AY618963 | Thailand | 1987 | DENV4 |
| AY618956 | Thailand | 1984 | DENV4 |
| AY618958 | Thailand | 1985 | DENV4 |
| AY618957 | Thailand | 1984 | DENV4 |
| AY618951 | Thailand | 1978 | DENV4 |
| AY618949 | Thailand | 1977 | DENV4 |
| JQ922559 | India | 1980 | DENV4 |

**Table S2** **Overview of the significant migration links between Yunnan and other areas.**

| From | To | BF | PP | Serotype |
| --- | --- | --- | --- | --- |
| China-other | Yunnan | *** | 1 | DENV1 |
| Myanmar | Yunnan | *** | 0.97 | DENV1 |
| Yunnan | China-other | ** | 0.75 | DENV1 |
| Yunnan | Myanmar | ** | 0.74 | DENV1 |
| Yunnan | Thailand | * | 0.47 | DENV1 |
| Yunnan | Malaysia | * | 0.28 | DENV1 |
| China-other | Yunnan | *** | 1 | DENV2 |
| Yunnan | China-other | *** | 1 | DENV2 |
| Yunnan | Malaysia | *** | 1 | DENV2 |
| Philippines | Yunnan | *** | 0.97 | DENV2 |
| Yunnan | Singapore | ** | 0.96 | DENV2 |
| Yunnan | Indonesia | ** | 0.63 | DENV2 |
| Laos | Yunnan | * | 0.19 | DENV2 |
| Laos | Yunnan | *** | 1 | DENV3 |
| China-other | Yunnan | *** | 1 | DENV3 |
| Myanmar | Yunnan | *** | 0.99 | DENV4 |
| Yunnan | China-other | *** | 0.98 | DENV4 |

*PP the posterior probability for a migration event between the involved locations.*

*BF, Bayes factor. 3 ≤ BF < 20 is considered as positive support, 20 ≤ BF < 150 as strong support and BF ≥ 150 as very strong support. Here, the strength of support for a migration link between locations is *, ** and *** for positive, strong and very strong support respectively.*

**Table S3** **Annual incidence of dengue in Laos, Myanmar, Thailand and Viet Nam.**

| Year | Laos | | | Myanmar | | | Thailand | | | Viet Nam | | |
| --- | --- | --- | --- | --- | --- | --- | --- | --- | --- | --- | --- | --- |
|  | Cases | Population | Incidence  /100,000 | Cases | Population | Incidence  /100,000 | Cases | Population | Incidence  /100,000 | Cases | Population | Incidence  /100,000 |
| 2013 | 44685 | 6541302 | 683.1209 | 20255 | 51852451 | 39.06276 | 154444 | 68144519 | 226.6419 | 52913 | 90752593 | 58.30467 |
| 2014 | 1716 | 6639763 | 25.8443 | 13806 | 52280807 | 26.4074 | 41082 | 68438748 | 60.0274 | 31848 | 91713850 | 34.7254 |
| 2015 | 1952 | 6741160 | 28.95644 | 43845 | 52680726 | 83.22778 | 144952 | 68714519 | 210.9481 | 97484 | 92677082 | 105.1867 |
| 2016 | 5617 | 6845848 | 82.04973 | 42913 | 53045226 | 80.89889 | 63931 | 68971313 | 92.69216 | 122020 | 93640435 | 130.307 |
| 2017 | 11039 | 6953031 | 158.7653 | 30000 | 53382581 | 56.19811 | 53961 | 69209817 | 77.96726 | 183287 | 94600643 | 193.7482 |
| 2018 | 6204 | 7061498 | 87.85671 | 23000 | 53708395 | 42.82385 | 86922 | 69428454 | 125.1965 | 126682 | 95545959 | 132.5875 |
| 2019 | 38753 | 7169456 | 540.5292 | 24900 | 54045420 | 46.07236 | 131157 | 69625581 | 188.3747 | 320702 | 96462108 | 332.4642 |
| 2020 | 7554 | 7275556 | 103.8271 | 4121* | 54409794 | 7.574004 | 71293 | 69799978 | 102.139 | 121398 | 97338583 | 124.7172 |

** Myanmar has reported 4, 121 dengue cases and 32 deaths as of 11 July 2020 without an update since.*

**Table S4** **Annual incidence of dengue in Yunnan.**

| Year | Imported Cases | Local Cases | Total cases |
| --- | --- | --- | --- |
| 2013 | 171 | 1432 | 1603 |
| 2014 | 194 | 139 | 333 |
| 2015 | 607 | 1509 | 2116 |
| 2016 | 308 | 217 | 525 |
| 2017 | 1704 | 1659 | 3363 |
| 2018 | 251 | 680 | 931 |
| 2019 | 1668 | 5172 | 6840 |
| 2020 | 15 | 245 | 260 |

**Table S5 Lagged correlation between Yunnan and border countries.**

| Lag | Viet Nam | | Thailand | | Laos | |
| --- | --- | --- | --- | --- | --- | --- |
|  | *P* | Pearson *r* | *P* | Pearson *r* | *P* | Pearson *r* |
| 0 | *** | **0.53** | *** | 0.27 | *** | 0.18 |
| 1 | *** | 0.43 | *** | 0.45 | *** | 0.43 |
| 2 | *** | 0.34 | *** | **0.55** | *** | **0.58** |
| 3 | *** | 0.33 | *** | 0.44 | ns | 0.49 |

****, < 0.001; **, 0.001~0.01; *, 0.01~0.05; ns, >0.05*

**Table S6 GLM of reported cases with climate factors and border restriction.**

|  | Estimate | Std. Error | *t* value | *P* |
| --- | --- | --- | --- | --- |
| Intercept | -45.42 | 7.32 | -6.20 | *** |
| Border restriction | -2.96 | 0.93 | -3.18 | ** |
| Relative humidity, 1-month lag | 0.21 | 0.06 | 3.59 | *** |
| Temperature, 1-month lag | 0.59 | 0.15 | 3.94 | *** |
| Month | 0.55 | 0.14 | 3.84 | *** |
| Year | 0.27 | 0.08 | 3.53 | *** |

****, <0.001; **, 0.001 ~0.01*

**Table S7 The correlation of climate factors between Yunnan and border countries.**

| Lag | Thailand | | | | | | Laos | | | | | | Vietnam | | | | | |
| --- | --- | --- | --- | --- | --- | --- | --- | --- | --- | --- | --- | --- | --- | --- | --- | --- | --- | --- |
|  | Temperature | | Precipitation | | Humidity | | Temperature | | Precipitation | | Humidity | | Temperature | | Precipitation | | Humidity | |
|  | *P* | *r* | *P* | *r* | *P* | *r* | *P* | *r* | *P* | *r* | *P* | *r* | *P* | *r* | *P* | *r* | *P* | *r* |
| 0 | *** | **0.83** | *** | **0.41** | *** | 0.67 | *** | **0.94** | *** | **0.80** | *** | 0.58 | *** | **0.91** | *** | **0.71** | *** | 0.66 |
| 1 | *** | 0.77 | ns | - | *** | **0.79** | *** | 0.74 | *** | 0.61 | *** | **0.71** | *** | 0.65 | *** | 0.56 | *** | **0.77** |
| 2 | *** | 0.46 | ns | - | *** | 0.70 | ** | 0.29 | * | 0.21 | *** | 0.67 | ns | - | * | 0.22 | *** | 0.66 |
| 3 | ns | - | ns | - | *** | 0.46 | ns | - | ns | - | *** | 0.49 | ns | - | ns | - | *** | 0.42 |

****, < 0.001; **, 0.001~0.01; *, 0.1~0.05; ns, > 0.05*

**Table S8 Correlation between effective reproductive number and weather variables.**

| Serotype | Lag | Average Temperature | | Suitability Score | | Relative Humidity | | Total Precipitation | |
| --- | --- | --- | --- | --- | --- | --- | --- | --- | --- |
|  |  | *P* | Pearson *r* | *P* | Pearson *r* | *P* | Pearson *r* | *P* | Pearson *r* |
| DENV1 | 0 | *** | 0.46 | *** | **0.71** | *** | **0.60** | *** | **0.68** |
|  | 1 | *** | 0.69 | *** | **0.71** | ** | 0.33 | *** | 0.67 |
|  | 2 | *** | **0.71** | *** | 0.54 | ns | / | *** | 0.49 |
|  | 3 | *** | 0.51 | * | 0.23 | ** | -0.33 | ns | / |
| DENV2 | 0 | ** | 0.33 | *** | **0.53** | ** | **0.36** | *** | **0.43** |
|  | 1 | *** | **0.45** | *** | 0.51 | ns | / | *** | 0.39 |
|  | 2 | *** | **0.45** | ** | 0.34 | ns | / | * | 0.23 |
|  | 3 | *** | 0.36 | ns | / | ** | -0.35 | ns | / |
| DENV3 | 0 | ns | / | ns | / | ** | **0.33** | ns | / |
|  | 1 | ** | 0.26 | *** | 0.37 | ** | 0.32 | ** | 0.32 |
|  | 2 | ** | **0.29** | *** | **0.47** | * | 0.27 | *** | **0.39** |
|  | 3 | * | 0.27 | *** | 0.41 | ns | / | ** | 0.31 |

****, <0.001; **, 0.001 ~0.01; *, 0.01 ~0.05;ns, > 0.05*

**Table S9 Uncertainty of correlation between effective reproductive number and weather variables (2.5% and 97.5% quartiles of Pearson *r,* at the most correlated lag).**

| Serotype | Average Temperature | | Suitability Score | | Relative Humidity | | Total Precipitation | |
| --- | --- | --- | --- | --- | --- | --- | --- | --- |
|  | 2.5% | 97.5% | 2.5% | 97.5% | 2.5% | 97.5% | 2.5% | 97.5% |
| DENV1 | 0.63 | 0.73 | 0.64 | 0.72 | 0.5 | 0.64 | 0.61 | 0.70 |
| DENV2 | 0.19 | 0.54 | 0.27 | 0.63 | 0.09 | 0.48 | 0.18 | 0.54 |
| DENV3 | -0.03 | 0.46 | 0.09 | 0.56 | -0.02 | 0.47 | 0.05 | 0.50 |

**Table S10** **Counts of transmission events between Yunnan and other areas.**

|  | Import | | Export | |
| --- | --- | --- | --- | --- |
| Serotype | Mean | 95% HPD | Mean | 95% HPD |
| DENV1 | 41.98 | [35, 49] | 12.09 | [1, 21] |
| DENV2 | 14.46 | [9, 19] | 38.17 | [28, 49] |
| DENV3 | 6.36 | [6, 7] | 0.34 | [0, 1] |
| DENV4 | 8.37 | [8, 10] | 3.03 | [1, 6] |

| Model | Variables | QAIC | Model | Variables | QAIC |
| --- | --- | --- | --- | --- | --- |
| Model 1 | Tem | 72428.41 | Model 18 | Pre.lag2 + Hum | 20726.54 |
| Model 2 | Tem.lag1 | 24555.60 | Model 19 | Pre.lag2 + Hum.lag1 | 19801.53 |
| Model 3 | Tem.lag2 | 32134.26 | Model 20 | Pre.lag2 + Hum.lag2 | 19683.85 |
| Model 4 | Tem.lag3 | 28439.78 | Model 21 | Pre.lag2 + Hum.lag3 | 21129.89 |
| Model 5 | Hum | 26994.11 | **Model 22** | **Pre.lag2 + BR** | 18036.62 |
| Model 6 | Hum.lag1 | 22437.38 | Model 23 | Pre.lag2 + BR + Tem | 19900.70 |
| Model 7 | Hum.lag2 | 22719.21 | Model 24 | Pre.lag2 + BR + Tem.lag1 | 16567.64 |
| Model 8 | Hum.lag3 | 32561.53 | Model 25 | Pre.lag2 + BR + Tem.lag2 | 18514.53 |
| Model 9 | Pre | 29398.24 | Model 26 | Pre.lag2 + BR + Tem.lag3 | 17555.15 |
| Model 10 | Pre.lag1 | 24394.54 | Model 27 | Pre.lag2 + BR + Hum | 17568.23 |
| **Model 11** | **Pre.lag2** | 20492.35 | **Model 28** | **Pre.lag2 + BR + Hum.lag1** | 15530.71 |
| Model 12 | Pre.lag3 | 28026.88 | Model 29 | Pre.lag2 + BR + Hum.lag2 | 16342.80 |
| Model 13 | BR | 28425.85 | Model 30 | Pre.lag2 + BR + Hum.lag3 | 18581.90 |
| Model 14 | Pre.lag2 + Tem | 22600.40 | Model 31 | Pre.lag2 + BR + Hum.lag1 + Tem | 15376.10 |
| Model 15 | Pre.lag2 + Tem.lag1 | 18828.46 | **Model 32** | **Pre.lag2 + BR + Hum.lag1 + Tem.lag1** | 13297.41 |
| Model 16 | Pre.lag2 + Tem.lag2 | 21053.84 | Model 33 | Pre.lag2 + BR + Hum.lag1 + Tem.lag2 | 15826.74 |
| Model 17 | Pre.lag2 + Tem.lag3 | 19870.79 | Model 34 | Pre.lag2 + BR + Hum.lag1 + Tem.lag3 | 15978.47 |

**Table S11** **QAIC results of variable selection using forward stepwise method.**

*Pre, total precipitation; Tem, average temperature; Hum, relative humidity; BR, border restriction.*

**References**

1. Kraemer MU, Sinka ME, Duda KA, et al. The global compendium of Aedes aegypti and Ae. albopictus occurrence. *Sci Data* 2015; **2**: 150035.

2. Faria NR, Quick J, Claro IM, et al. Establishment and cryptic transmission of Zika virus in Brazil and the Americas. *Nature* 2017; **546**(7658): 406-10.
