# Supplementary material for: Assessing the impact of COVID-19 border restrictions on dengue transmission in Yunnan Province, China: an observational epidemiological and phylogenetic analysis
Source: Lancet Reg Health West Pac. 2021 Aug 20;14:100259. doi: 10.1016/j.lanwpc.2021.100259 (PMC8387751; doi:10.1016/j.lanwpc.2021.100259)
Supplement: Supplementary file 2 [file mmc2.docx]

|  | Item No | Recommendation | Relevant section of the manuscript |
| --- | --- | --- | --- |
| **Title and abstract** | 1 | (*a*) Indicate the study’s design with a commonly used term in the title or the abstract | Title |
|  |  | (*b*) Provide in the abstract an informative and balanced summary of what was done and what was found | Abstract |
| Introduction |  |  |  |
| Background/rationale | 2 | Explain the scientific background and rationale for the investigation being reported | Introduction section, end of second paragraph |
| Objectives | 3 | State specific objectives, including any prespecified hypotheses | Introduction section, Fourth paragraph |
| Methods |  |  |  |
| Study design | 4 | Present key elements of study design early in the paper | Abstract  Introduction section, Fourth paragraph  Methods, fifth paragraph |
| Setting | 5 | Describe the setting, locations, and relevant dates, including periods of recruitment, exposure, follow-up, and data collection | Methods, paragraphs 1-4 |
| Participants | 6 | (*a*) Give the eligibility criteria, and the sources and methods of selection of participants | Methods, first paragraph |
| Variables | 7 | Clearly define all outcomes, exposures, predictors, potential confounders, and effect modifiers. Give diagnostic criteria, if applicable | Methods, “statistical analysis” section |
| Data sources/ measurement | 8* | For each variable of interest, give sources of data and details of methods of assessment (measurement). Describe comparability of assessment methods if there is more than one group | Methods sections “ Climatic data and Vector Suitability Scores” (climate data) and “Statistical analysis” (border restrictions [primary exposure]) |
| Bias | 9 | Describe any efforts to address potential sources of bias | Methods- experimental design approach with independent datasets (epidemiological and genetic) |
| Study size | 10 | Explain how the study size was arrived at | NA, observational study with CIs of measured effects presented throughout |
| Quantitative variables | 11 | Explain how quantitative variables were handled in the analyses. If applicable, describe which groupings were chosen and why | Methods, section “statistical analysis” |
| Statistical methods | 12 | (*a*) Describe all statistical methods, including those used to control for confounding | Methods, section “statistical analysis” |
|  |  | (*b*) Describe any methods used to examine subgroups and interactions | Methods, section “statistical analysis” last paragraph (synchrony of dengue incidence between countries) |
|  |  | (*c*) Explain how missing data were addressed | Results section, third paragraph (omission of Myan mar from the analysis) |
|  |  | (*d*) If applicable, describe analytical methods taking account of sampling strategy | NA |
|  |  | (*e*) Describe any sensitivity analyses | NA |
| Results |  |  |  |
| Participants | 13* | (a) Report numbers of individuals at each stage of study—eg numbers potentially eligible, examined for eligibility, confirmed eligible, included in the study, completing follow-up, and analysed | Results section, first paragraph |
|  |  | (b) Give reasons for non-participation at each stage | NA |
|  |  | (c) Consider use of a flow diagram | Considered not relevant for this study |
| Descriptive data | 14* | (a) Give characteristics of study participants (eg demographic, clinical, social) and information on exposures and potential confounders | Figure 1 and 2 |
|  |  | (b) Indicate number of participants with missing data for each variable of interest | NA |
| Outcome data | 15* | Report numbers of outcome events or summary measures | Results section, first paragraph |
| Main results | 16 | (*a*) Give unadjusted estimates and, if applicable, confounder-adjusted estimates and their precision (eg, 95% confidence interval). Make clear which confounders were adjusted for and why they were included | Results section, end of second paragraph |
|  |  | (*b*) Report category boundaries when continuous variables were categorized | NA |
|  |  | (*c*) If relevant, consider translating estimates of relative risk into absolute risk for a meaningful time period | Results section, end of second paragraph |
| Other analyses | 17 | Report other analyses done—eg analyses of subgroups and interactions, and sensitivity analyses | Results section, third paragraph |
| Discussion |  |  |  |
| Key results | 18 | Summarise key results with reference to study objectives | Discussion section, first paragraph |
| Limitations | 19 | Discuss limitations of the study, taking into account sources of potential bias or imprecision. Discuss both direction and magnitude of any potential bias | Discussion section, paragraphs 5-7 |
| Interpretation | 20 | Give a cautious overall interpretation of results considering objectives, limitations, multiplicity of analyses, results from similar studies, and other relevant evidence | Discussion section, end of first paragraph |
| Generalisability | 21 | Discuss the generalisability (external validity) of the study results | Discussion section, second paragraph |
| Other information |  |  |  |
| Funding | 22 | Give the source of funding and the role of the funders for the present study and, if applicable, for the original study on which the present article is based | Acknowledgements section |
