# Supplementary material for: Assessing the impact of COVID-19 border restrictions on dengue transmission in Yunnan Province, China: an observational epidemiological and phylogenetic analysis
Source: Lancet Reg Health West Pac. 2021 Aug 20;14:100259. doi: 10.1016/j.lanwpc.2021.100259 (PMC8387751; doi:10.1016/j.lanwpc.2021.100259)
Supplement: Supplementary file 3 [file mmc3.docx]

*Disclaimer: This translation in Chinese was submitted by the authors and we reproduce it as supplied. It has not been peer reviewed. Our editorial processes have only been applied to the original abstract in English, which should serve as reference for this manuscript.*

**摘要**

**背景**

为应对COVID-19在世界范围内的大流行，中国对跨境旅行实施了严格的管控措施，以防止境外疫情输入。云南位于中国西南边陲，毗邻登革热疫情严重的东南亚国家，其登革热疫情在2020年明显缓解，病例数由2019年的6840例降至260例。

**方法**

我们综合使用2013-2020年云南及周边国家的流行病学和病毒基因组数据，描述输入病例在推动云南登革热动态中的作用，并评估近期施行的国际旅行限制措施与2020年云南登革热病例数下降之间的关联。

**发现**

足够证据表明，云南2013-2019年的登革热疫情与境外输入病例密切相关。我们发现（1）云南登革热流行相较于周边地区有0至2个月的滞后，且这种滞后并非气候差异所致；（2）云南在冬季并无登革热的本地流行；（3）使用病毒基因组序列估算的有效再生数和登革病毒系统发育树同样表明云南并非登革热的流行地区。多变量回归模型结果显示，在纳入了环境因素的影响后，云南2020年登革热病例显著下降仍与国际旅行限制具有明显的关联。

**解释**

我们认为云南目前仍是登革热传播的输入地，2020年执行的关口管控措施可能避免了上千例登革热感染，大大降低了疾病负担。对从高风险地区返回的旅行者进行有针对性的病原检测和疾病监测，将有助于制定公共卫生战略，也可最大限度地减少甚至消除登革热在非地方流行性地区的爆发，如中国南方。

**基金**

本研究得到以下基金的支持：北京市科技计划课题(Z201100005420010)；北京市自然科学基金（JQ18025）；北京市地表科学创新工程；国家自然科学基金（82073616）；中国科协青年人才托举工程(2018QNRC001)；H.T., O.P.G.和M.U.G.K.致谢牛津马丁学院的支持；O.J.B.得到了Wellcome Trust Sir Henry Wellcome Fellowship (206471/Z/17/Z) 资助。基金资助者未参与研究设计、数据收集与分析、决定出版或撰写文章等工作。

**关键词** COVID-19关口管控措施；登革热扩张；蚊媒病毒；疫情封锁措施
